# Dysregulation of the microbiota-gut-brain axis induced by chronic pancreatitis mediates anxiety- and depression-like behaviors in mice

**DOI:** 10.3389/fimmu.2025.1753424

**Published:** 2026-01-14

**Authors:** Leheng Liu, Xi Zhang, Chuanyang Wang, Kena Zhou, Jingpiao Bao, Zhiyuan Cheng, Haoran Sun, Chengying Zhu, Ge Yu, Wenqin Xiao, Rong Wan

**Affiliations:** 1 Department of Gastroenterology, Shanghai General Hospital, Shanghai Jiao Tong University School of Medicine, Shanghai, China; 2 Shanghai Key Laboratory of Pancreatic Diseases, Shanghai Jiao Tong University School of Medicine, Shanghai, China; 3 School of Basic Medical Science, Center for Big Data and Population Health of Institute of Health and Medicine (IHM), Anhui Medical University, Hefei, China; 4 Institute of Health and Medicine, Hefei Comprehensive National Science Center, Hefei, China

**Keywords:** anxiety, chronic pancreatitis, depression, gut microbiota, MGB axis

## Abstract

**Background:**

Anxiety and depression are common psychological complications in patients with chronic pancreatitis (CP). This study aims to investigate the dysregulation of microbiota-gut-brain axis induced by CP in driving anxiety- and depression-like behaviors in mice.

**Methods:**

C57BL/6J mice injected with caerulein (Cae) were used to establish an experimental CP model. Behavioral tests were performed to assess anxiety- and depression-like behaviors. The gut microbiota composition and serum metabolites were analyzed via 16S rRNA sequencing and liquid chromatography–mass spectrometry, respectively. Antibiotic cocktail (ABX) and fecal microbiota transplantation (FMT) models were employed to validate the direct role of the gut microbiota, while mixed probiotic gavage was used for intervention evaluation.

**Results:**

Cae-injected mice presented typical CP symptoms and significant anxiety- and depression-like behaviors. 16S rRNA sequencing revealed altered gut microbiota composition in CP mice, with a reduced abundance of *Lactobacillus* and enrichment of *Helicobacter*. ABX and FMT experiments confirmed that the CP-derived gut microbiota can independently induce anxiety/depression-like behaviors. In CP mice, Serum corticosterone and its metabolite levels were significantly increased in CP mice. CP-induced microbiota dysbiosis can induce the downregulation of intestinal barrier and blood–brain barrier functions, increase inflammatory levels, and extensively dysregulate neurotransmitter transmission in the brain. Treatment with mixed probiotics improved anxiety- and depression-like behaviors in CP mice.

**Conclusions:**

CP promotes anxiety- and depression-like behaviors in mice by inducing gut microbiota dysbiosis. This study provides experimental evidence for the use of microbiota-targeted interventions for treating the psychological complications of CP.

## 1 Introduction

Chronic pancreatitis (CP) is a progressive inflammatory and fibrotic disorder often accompanied by severe complications, including persistent pain, exocrine and endocrine dysfunction, and neuropsychiatric manifestations such as anxiety and depression (1). As prevalent comorbidities in CP patients, anxiety and depression not only intensify the clinical burden but also correlate with accelerated disease progression and poorer treatment outcomes (2, 3). Despite their considerable clinical impact, the exact mechanisms linking CP to anxiety and depression remain poorly understood.

The gut microbiota is dynamically regulated by a variety of host and environmental factors. Emerging evidence has confirmed that pancreatic exocrine dysfunction significantly influences the intestinal microecology (4). Consistently, intestinal microbiota dysbiosis has been observed in both CP patients and experimental CP models (5–8). Intestinal microbiota dysbiosis not only exacerbates intestinal barrier dysfunction but also regulates central nervous system (CNS) activity via the microbiota–gut–brain (MGB) axis (9). Gut microbiota dysbiosis can affect immune responses, promoting the entry of lipopolysaccharide (LPS) and cytokines into the blood, which further impacts the integrity of the blood–brain barrier (BBB), promote the activation of the (hypothalamic-pituitary-adrenal) HPA axis and triggers CNS inflammation (10). Additionally, microbiota-derived metabolites such as short-chain fatty acids (SCFAs) (11), tryptophan (12), and the neurotransmitter precursor γ-aminobutyric acid (GABA) (13) act as key mediators of neuroendocrine signaling and emotional regulation, exerting regulatory functions in the crosstalk of the MGB axis.

While alterations in the gut microbiota and symptoms of anxiety/depression in individuals with CP have been reported independently, the intricate relationships among pancreatic inflammation, microbial ecology, and neuropsychiatric manifestations require systematic investigation. Therefore, this study established an experimental CP mouse model to evaluate alterations in the MGB axis and its functional impacts on anxiety- and depression-like behaviors.

## 2 Materials and methods

### 2.1 Establishment of an experimental CP mouse model and probiotic administration

Eight-week-old male C57BL/6 mice were purchased from the Animal Center of Shanghai General Hospital and randomly assigned to different groups. After a one-week adaptation period, the mice received intraperitoneal injections of caerulein (Cae; 100 μg/kg) for 5 consecutive weeks to establish the experimental CP model, with 3 days per week, 6 injections per day, and an interval of 1h between each injection. Normal control (NC) mice were given simultaneous intraperitoneal injections of normal saline at the same volume.

In a separate experimental set, 8-week-old male C57BL/6 mice were randomly assigned to three groups: (i) CP + Mixed Probiotics (MP) Group: *Lactobacillus rhamnosus* (BNCC187896), *Lactobacillus helveticus* (BNCC187926), *Lactobacillus reuteri* (BNCC192190), and *Lactobacillus acidophilus* (BNCC336636) were mixed at equal ratios. Each mouse received an oral gavage of 1×10^9^ CFU of the probiotic mixture dissolved in 0.2 mL of normal saline per day. One week later, the mice were administered Cae injections at the same dosage and frequency as previously described. (ii) The CP group and (iii) NC group were subjected to identical treatment protocols as above, with the same frequency and volume of normal saline being administered via oral gavage as those in group (i).

All intraperitoneal injections and gavage operations were performed during the light cycle. After model establishment, the mice were fasted for 12h and then euthanized using CO_2_ at a flow rate of 50% of the chamber volume per minute. All experimental procedures were approved by the Institutional Animal Care and Use Committee of Shanghai General Hospital.

### 2.2 Antibiotic-mediated gut microbiota depletion and FMT

Eight-week-old male C57BL/6 mice were subjected to daily oral gavage with 200 μL of an antibiotic cocktail (ABX; 1 g/L ampicillin, neomycin and metronidazole, and 0.5 g/L vancomycin) for 2 consecutive weeks to deplete the gut microbiota. Fresh fecal samples from NC and CP mice were collected and resuspended at a concentration of 0.1 g/mL in PBS containing 20% glycerol. After centrifugation at 1500 rpm for 3 mins, larger particles were precipitated. Normalize each group’s bacterial solution to a concentration of 1×10^9^ CFU/mL. Following the 2-week ABX treatment, the recipient mice were gavaged daily with 200 μL of the fecal suspension for an additional 2 weeks to establish a fecal microbiota transplantation (FMT) model.

### 2.3 Behavioral tests

Behavioral tests were conducted in the following sequence: the open field test (OFT), elevated plus maze test (EPMT), tail suspension test (TST), forced swim test (FST) and sucrose preference test (SPT), with each test separated by a 24h interval.

OFT: Mice were placed in the central zone of a 40×40×40 cm square open area, and their movement trajectories were recorded for 10 mins via a camera.

EPMT: Mice were tested on a 40-cm-high cross-shaped maze consisting of two open arms (30×5 cm), two closed arms (30×5 cm with 15-cm-high walls) and a central zone (5×5 cm). Each mouse was placed at the center, and their movement trajectories were recorded for 6 mins via a camera.

Movement trajectories in the OFT and EPMT were annotated via the CVAT labeling tool (https://www.cvat.ai/). Python (version 3.9.7) was used to calculate the time spent in the central zone and the number of central entries in the OFT and the time spent in the open arms and the number of open-arm entries in the EPMT.

TST: Mice were suspended by the tail with medical tape (1 cm from the tip), and a plastic cylinder was placed over the tail to prevent climbing. Behavioral responses were recorded for 6 mins, and the immobility time during the last 4 mins was quantified.

FST: Mice were placed in a transparent beaker containing 800 mL of water. Swimming behavior was recorded for 6 min, and the immobility time during the final 4 min was analyzed.

SPT: One day before testing, the mice were provided with identical bottles containing purified water or 2% (w/v) sucrose solution. The positions of the two bottles were swapped after 12h. On the test day, the bottles were returned to their initial positions, and fluid consumption was recorded over 24h. Sucrose preference was calculated as the ratio of sucrose solution intake to total fluid intake.

### 2.4 H&E and Masson staining

Tissues were fixed in 4% paraformaldehyde for 24 h, then embedded in paraffin and cut into 4μm sections. After a series of dewaxing and hydration process, Tissue sections were stained with H&E (G1076, Servicebio, China) and Masson (G1340, Solarbio, China) staining. Images were captured by Leica DMI8. The data of H&E and Masson staining was analyzed by ImageJ software (version 1.54f). Colonic villus atrophy was scored by two pathologists in a blinded manner based on the following criteria: 0 = normal villus morphology with uniform length, no atrophy or shortening; 1 = mild atrophy (length shortened by ≤25%) with intact overall morphology; 2 = moderate atrophy (length shortened by 26%–50%) with partial morphological disorder; 3 = severe atrophy (length shortened by >50%) or complete villus disappearance.

### 2.5 Immunofluorescence

Paraffin embedding, sectioning, and dewaxing-hydration procedures were performed as described above. After antigen retrieval processes, the tissue sections were blocked with blocking buffer (Beyotime, Nanjing, China) for 1h. Primary antibodies against ZO-1 (1:100, 21773-1-AP, Proteintech, China), Claudin-1 (1:1000, GB112543, Servicebio, China), IBA1 (1:250, 81728-1-RR, Proteintech, China), and α-SMA (1:500, sc-32251, Santa Cruz, TX, USA) were respectively incubated overnight at 4°C. After rewarming at room temperature for 1 h, the primary antibodies were washed off with PBS, and then the sections were incubated with YSFluor™488 Goat Anti-Rabbit antibody (1:200, 33106ES60, Yeasen, China) or YSFluor™488 Goat Anti-Mouse antibody (1:200, 33206ES60, Yeasen, China) in the dark for 1 h. Subsequently, the nuclei were stained with DAPI (1:1000, 40728ES, Yeasen, China). Immunofluoresce images of the colon and pancreas were captured using Leica DMI8, and Z-stack imaging of IBA1 in the brain was performed using a Zeiss LSM980. Images were analyzed by ImageJ software (version 1.54f).

### 2.6 Fecal 16S rRNA sequencing

Total microbial genomic DNA was extracted from mouse fecal pellets using HiPure Stool DNA Kits (Magen, Guangzhou, China). The quality of genomic DNA was detected by NanoDrop 2000 (Thermo Fisher) and agarose gel electrophoresis.

DNA was amplified by PCR using specific primers (341F: CCTACGGGNGGCWGCAG; 806R: GGACTACHVGGGTATCTAAT) targeting the V3-V4 region of 16S rRNA with barcode and Q5^®^ High-Fidelity DNA Polymerase (New England Biolabs, MA, USA). The amplification system was as follows: 5×Q5^®^ Reaction Buffer, 10 μL; 5×Q5^®^ High GC Enhancer, 10 μL; 2.5 mM dNTPs, 1.5 μL; 10 μM Primer F, 1.5 μL; 10 μM Primer R, 1.5 μL; Q5^®^ High-Fidelity DNA Polymerase, 0.2 μL; Template, 50 ng; Nuclease-free Water, Up to 50 μL. The amplification procedure was as follows: pre-denaturation at 95°C for 5 min; denaturation-annealing-extension cycle at 95°C for 1 min/60°C for 1 min/72°C for 1 min, 30 cycles; extension at 72°C for 7 min. The PCR products were purified using AMPure XP Beads (Beckman, CA, USA) and then quantified by Qubit3.0. The sequencing library was constructed using Illumina DNA Prep Kit (Illumina, CA, USA). Library quality was detected by ABI StepOnePlus Real-Time PCR System (Life Technologies, CA, USA). Qualified libraries were sequenced on Novaseq 6000 in PE250 mode with pooling.

### 2.7 RNA extraction and qPCR detection

Total RNA was extracted from colon and brain tissues using TRIZOL (Takara, Japan). Following the manufacturers’ instructions, RNA was reverse-transcribed into cDNA using the HyperScript III RT SuperMix kit (EnzyArtisan, Shanghai, China), and real-time qPCR was performed using the Universal SYBR qPCR Mix kit (EnzyArtisan, Shanghai, China). The primers used are as follows:IL-6 Forward, CTCCCAACAGACCTGTCTATAC; IL-6 Reverse, CCATTGCACAACTCTTTTCTCA; TNF-α Forward, TCTCTTCAAGGGACAAGGCTG; TNF-α Reverse, ATAGCAAATCGGCTGACGGT; GAPDH Forward, CATCACTGCCACCCAGAAGACTG; GAPDH Reverse, ATGCCAGTGAGCTTCCCGTTCAG.

The mRNA expression levels of the target genes were normalized to those of GAPDH and quantified using the 2^-ΔΔ^Ct method.

### 2.8 Protein extraction and western blot

Total protein was extracted from tissues using RIPA lysis buffer (EpiZyme, Shanghai, China) supplemented with a protease inhibitor (EpiZyme, Shanghai, China). Equal amounts of protein samples were loaded onto 10% SDS-PAGE gels for electrophoresis, followed by transfer to polyvinylidene difluoride membranes (Millipore, MA, US). The membranes were blocked with 5% nonfat milk to prevent nonspecific binding, then incubated overnight at 4°C with primary antibodies against ZO-1 (1:5000, 21773-1-AP, Proteintech, China), Claudin-1 (1:500, GB112543, Servicebio, China), and β-actin (1:20000, 66009-1-Ig, Proteintech, China). On the following day, the membranes were incubated with HRP-conjugated goat anti-rabbit secondary antibody (1:100,000, Jackson) or HRP-conjugated goat anti-mouse secondary antibody (1:5000, Proteintech, China) for 1h at room temperature. Target protein bands were visualized using the ECL chemiluminescence system, and band intensities were quantified using ImageJ software (version 1.54f).

### 2.9 Serum LPS and corticosterone detection

Serum samples were stored at -80°C. For detection, the serum was diluted at an appropriate ratio and assayed for LPS using the LPS ELISA Kit (RK04263, Abclonal, China), and for corticosterone using the corticosterone ELISA Kit (PC100, Beyotime, China) following the manufacturer’s instructions.

### 2.10 Serum untargeted metabolomics sequencing

Serum samples were stored in a -80°C refrigerator. For detection, the samples were placed on dry ice, 100 μL of each sample was pipetted, and 400 μL of extraction solution (methanol:acetonitrile = 3:1, pre-cooled at -40°C) was added. The mixture was vortexed for 5 min, sonicated for 15 min, and then left to stand at 4°C for 1 h. The samples were taken out and centrifuged at 12,000 rpm at 4°C for 15 min. An equal volume of supernatant was collected and concentrated to dryness under vacuum. 50 μL of 50% methanol aqueous solution (methanol:water = 1:1, v/v) was added for reconstitution, followed by vortexing for 3 min (4°C, 2000 rpm) and centrifugation at 12,000 rpm at 4°C for 15 min. The supernatant was taken for injection analysis.

For Liquid Chromatography-Mass Spectrometry (LC-MS) analysis, samples were placed in an 8°C autosampler and separated using an ultra-high performance liquid chromatography system with an HSS T3 column. The injection volume was 2 μL, column temperature was maintained at 40°C, and flow rate was 0.3 mL/min. The chromatographic mobile phases consisted of: A (0.1% formic acid in water), B (0.1% formic acid in methanol), C (0.05% acetic acid in water), and D (0.05% acetic acid in methanol).

Each sample was detected in positive and negative ion modes using electrospray ionization. After UHPLC separation, samples were analyzed by a Thermo QE HF-X mass spectrometer. The mass spectrometry conditions were as follows: sheath gas flow rate: 50; auxiliary gas: 13; spray voltage: 2.5 KV (+)/2.5 KV (-); S-Lens RF: 50; capillary temperature: 325°C; auxiliary gas temperature: 300°C; normalized collision energy: 30; Top N = 10. The scanning range was 70–1050 m/z, and the scanning mode was separate scanning for positive and negative ions. Xcalibur (version 4.1) was used for analysis.

Raw data were processed using Compound Discovery software for peak alignment, retention time correction, and peak area extraction. For metabolite structure identification, Compound Discovery was used to search databases through accurate mass matching (<10 ppm) and secondary spectrum matching. The downstream data processing was performed based on the Linux operating system and using R and Python software. All experimental procedures were performed by APExBIO Technology.

### 2.11 Transcriptome sequencing

Total RNA was extracted from tissues using TRIZOL (Takara, Japan). The quality of RNA was detected by NanoDrop 2000, Agilent 2100 and agarose gel electrophoresis. mRNA transcriptome library construction was performed using the Hieff NGS^®^ Ultima Dual-mode mRNA Library Prep Kit (12309ES, Yeasen, China). In short, mRNA was enriched with mRNA Capture Beads, and after bead purification, mRNA was fragmented at high temperature. Using the fragmented mRNA as a template, the first strand of cDNA was synthesized in a reverse transcriptase mixture system. While synthesizing the second strand of cDNA, end repair and A-tailing were completed. Subsequently, adapters were ligated, and target fragments were purified and selected using Hieff NGS^®^ DNA Selection Beads. Then, PCR library amplification was performed, and finally, detection was carried out using the Illumina Novaseq X Plus (Illumina, USA).

### 2.12 Statistical analysis

The data are presented as the means ± SEM. Statistical significance was determined via GraphPad Prism 9.0.2 and R software 4.3.2. For parametric tests, unpaired t tests or one-way ANOVA were used. Two-way ANOVA was applied to analyze repeated-measures data. Spearman correlation coefficients were used to analyze the relationships between different variables. A p value less than 0.05 was considered statistically significant.

## 3 Results

### 3.1 CP induces anxiety- and depression-like behaviors in mice

**Figure 1:**
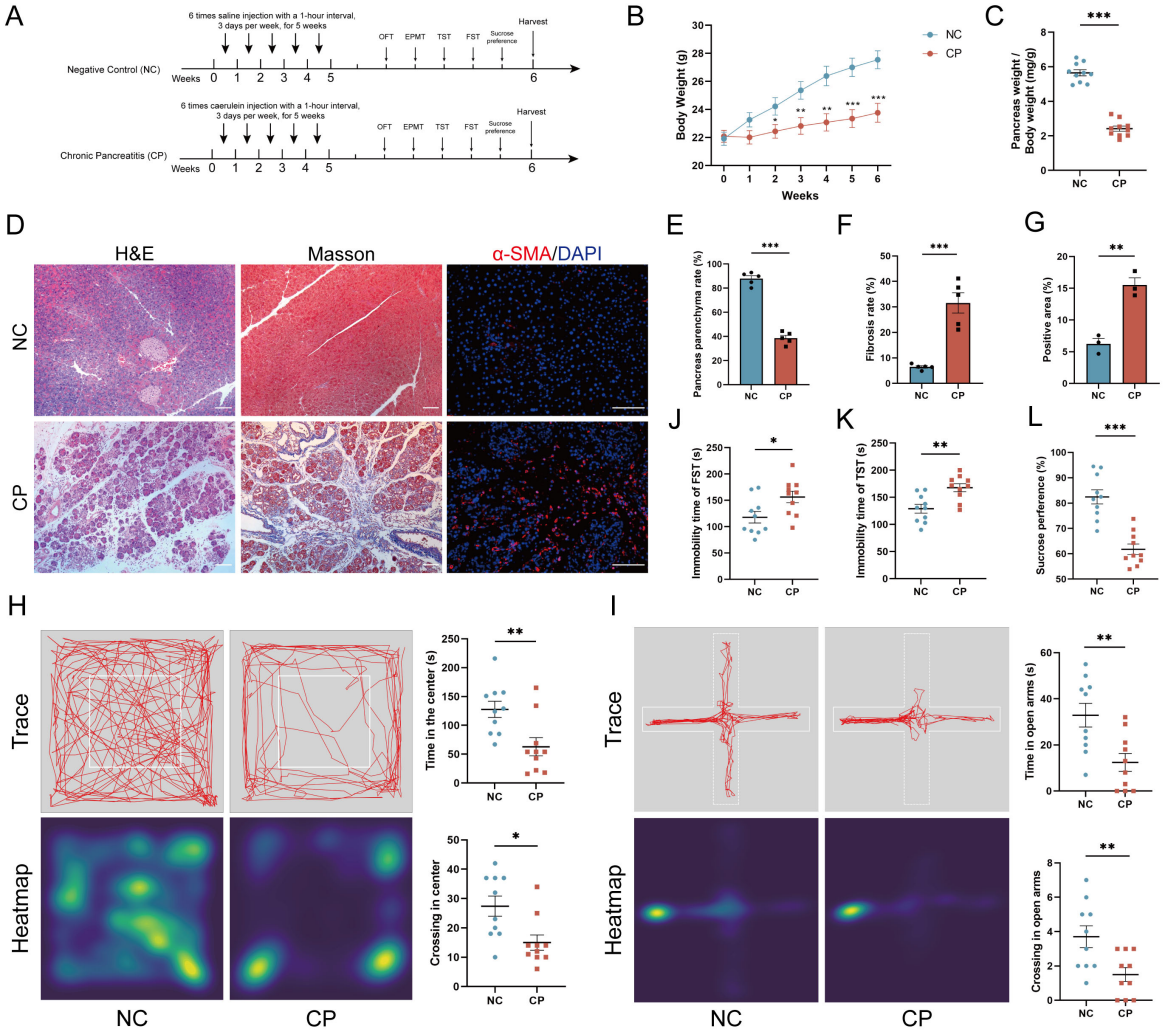
Cae-induced experimental CP mice exhibit anxiety- and depression-like behaviors. **(A)** Experimental design for establishing a Cae-induced CP mouse model. **(B, C)** Body weight changes **(B)** and pancreas weight ratio **(C)** of CP and NC mice. **(D–G)** Representative H&E, Masson (200× magnification), and α-SMA immunofluorescence staining (400× magnification) images of pancreatic body tissues from CP and NC mice **(D)**, along with statistical analysis results **(E–G)**. Scale bar, 100 μm. **(H, I)** Representative trajectory traces, heatmap, and statistical analysis for CP and NC mice in OFT **(H)** and EPMT **(I)** (n=10). **(J–L)** Statistical analysis of immobility time for CP and NC mice in FST **(J)** and TST **(K)**, and sucrose consumption ratio **(L)** (n=10). Cae, caerulein; CP, chronic pancreatitis; NC, normal control; OFT, open field test; EPMT, elevated plus maze test; TST, tail suspension test; FST, forced swim test. *p<0.05; **p<0.01; ***p<0.001.

To investigate whether CP mice exhibit anxiety- and depression-like behaviors, 8-week-old male C57BL/6 mice were injected with Cae continuously for 5 weeks. Five behavioral tests were conducted, and the mice were euthanized one day after the last test was completed (**Figure 1A**). Compared with those of NC mice, the body weights and pancreatic weights of CP mice were significantly lower (P < 0.001; **Figures 1B, C**). H&E staining showed that CP mice presented widespread pancreatic acinar atrophy, a significantly decreased parenchyma ratio (P<0.001; **Figures 1D, E**), excessive fibrous connective tissue hyperplasia, and elevated inflammatory cell infiltration. Masson staining further identified a notable enlargement of pancreatic fibrotic areas (P<0.01; **Figures 1D, F**), accompanied by abundant reticular collagen fiber proliferation and prominent periductal collagen deposition. Moreover, immunofluorescence indicated a marked expansion of α-SMA-positive regions (P<0.01; **Figures 1D, G**), confirming the presence of typical chronic pancreatitis (CP) pathological features in the model mice.

Compared with NC mice, CP mice in the OFT presented significantly shorter times (127.70 ± 14.04 vs 62.90 ± 15.65, P = 0.0064) and fewer entries (27.40 ± 3.44 vs 15.00 ± 2.65, P = 0.0103) into the center area (**Figure 1H**). Similarly, in the EPMT, CP mice spent less time (32.90 ± 5.13 vs 12.40 ± 3.85, P = 0.0050) and made fewer entries (3.70 ± 0.63 vs 1.50 ± 0.40, P = 0.0089) into the open arms (**Figure 1I**), collectively indicating the presence of anxiety-like behaviors in CP mice. Moreover, in the depression-related behavioral tests, CP mice had longer immobility times in the FST (117.9 ± 10.93 vs 156.4 ± 10.93, P = 0.0227) (**Figure 1J**) and TST (129.0 ± 8.09 vs 167.8 ± 7.25, P = 0.0022) (**Figure 1K**) and a decreased preference for sucrose solution (82.44 ± 2.78 vs 61.77 ± 2.07, P < 0.0001) (**Figure 1L**). These results indicate that CP mice exhibit significant anxiety- and depression-like behaviors.

### 3.2 CP induces intestinal microbiota dysbiosis in mice and leads to a reduction in *Lactobacillus*

**Figure 2:**
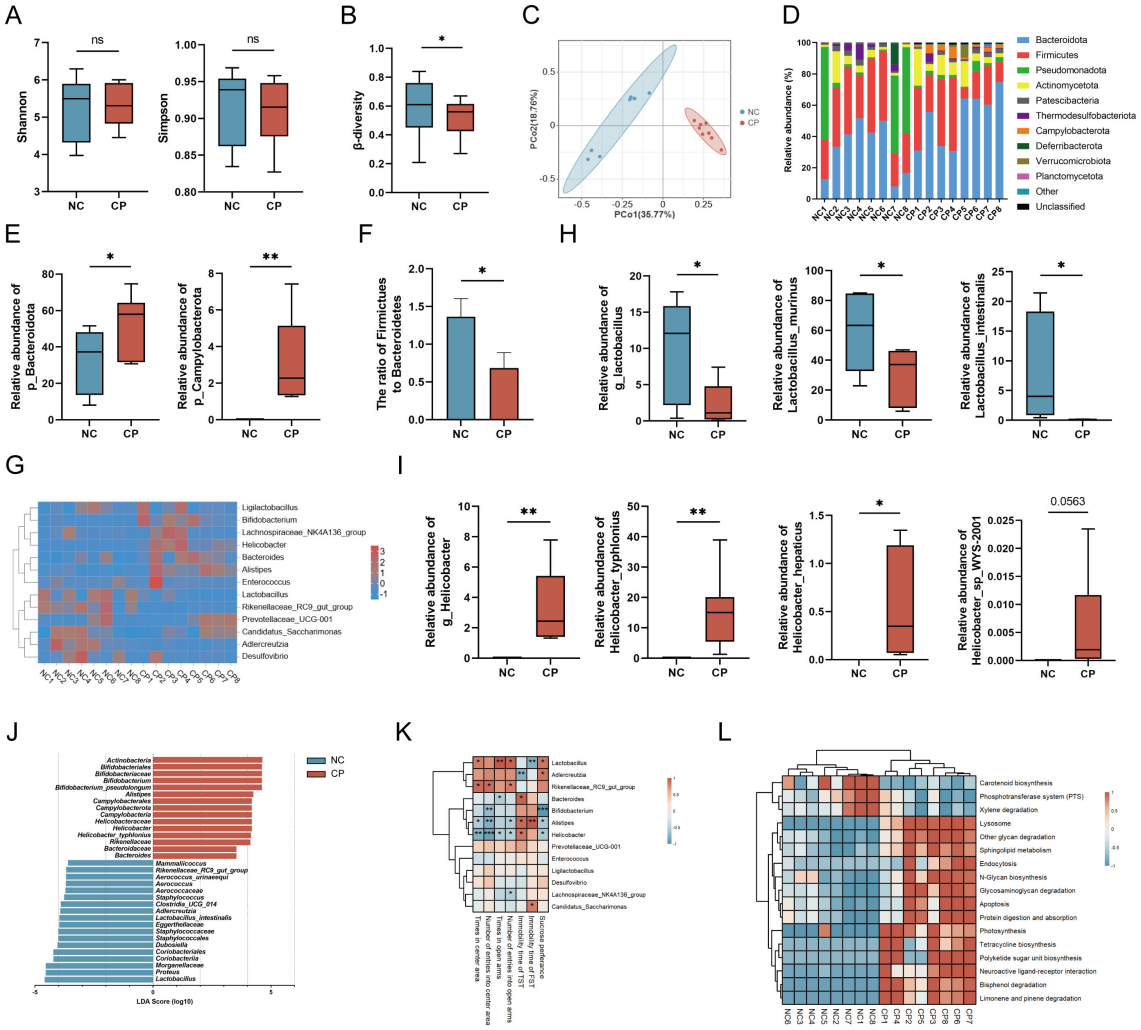
CP induces a reduction of *Lactobacillus* in mice. **(A–C)** Assessment of difference in fecal microbiota between CP and NC mice via the Shannon and Simpson index **(A)**, β-diversity **(B)** and PCoA **(C)**. **(D)** Phylum-level composition of fecal microbiota in CP and NC mice. **(E)** Relative abundances of *Bacteroidota* and *Campylobacterota* in CP and NC mice. **(F)***Firmicutes*/*Bacteroidota* ratio in CP and NC mice. **(G)** Genus-level composition of fecal microbiota in CP and NC mice. **(H)** Relative abundances of *Lactobacillus*, *Lactobacillus murinus*, and *Lactobacillus intestinalis* in fecal microbiota of CP and NC mice. **(I)** Relative abundances of *Helicobacter*, *Helicobacter typhlonius*, *Helicobacter hepaticus* and *Helicobacter* sp.*WYS-2001* in fecal microbiota of CP and NC mice. **(J)** LEfSe analysis of differentially abundant microbiota from domain to species levels in fecal samples of CP and NC mice. Thresholds: LDA score>3.5 and P<0.05. **(K)** Spearman correlation analysis between relative abundances of differentially abundant genus and results of behavioral tests. **(L)** Heatmap of PICRUSt2 functional enrichment analysis for different bacteria of CP and NC mice. PCoA, principal coordinate analysis; LEfSe, linear discriminant analysis effect size; LDA, linear discriminant analysis. PICRUSt2, phylogenetic investigation of communities by reconstruction of unobserved states. *p<0.05; **p<0.01.

To explore the potential involvement of intestinal microbial dysbiosis in the development of CP-related anxiety- and depression-like behaviors, we first performed 16S rRNA sequencing on fecal samples. Compared with that in NC mice, the α-diversity of the fecal microbiota in CP mice was not significantly different (**Figure 2A**). However, the β-diversity was reduced (P<0.05, **Figure 2B**), and the clustering patterns of the two groups were significantly different (**Figure 2C**). At the phylum level, CP mice presented significantly increased abundances of *Bacteroidota* and *Campylobacterota* (P < 0.05; **Figures 2D, E**), accompanied by a decreased *Firmicutes*/*Bacteroidota* ratio (P < 0.05; **Figure 2F**). At the genus level, a marked downregulation of the probiotic genus *Lactobacillus* was observed in CP mice, with *Lactobacillus murinus* (P < 0.05) and *Lactobacillus intestinalis* (P < 0.05) being the most significantly affected species (**Figures 2G, H**). Notably, the pathogens *Helicobacter*, particularly *Helicobacter typhlonius* (14) (P < 0.01), *Helicobacter hepaticus* (15, 16) (P < 0.05), and *Helicobacter* sp. *WYS-2001* (P = 0.0563), were enriched in CP mice (**Figures 2G, I**). LEfSe analysis (LDA threshold set at 3.5 and P<0.05) showed that there were significantly different taxa in the gut microbiota between the CP group and the NC group. Among them, *Lactobacillus* was the key biomarkers driving the microbial community differences between the two groups (**Figure 2J**, **Supplementary Table S1**). Notably, multiple detected differential genera—especially *Lactobacillus*—were correlated with behavioral test outcomes, supporting their potential involvement in CP-associated psychological symptoms (**Figure 2K**). Functional prediction indicated that the dominant microbiota in CP mice was significantly enriched in pathways such as neuroactive ligand–receptor interaction, apoptosis, endocytosis, and glycosaminoglycan degradation (**Figure 2L**). These results indicate that the structure of the gut microbiota in CP mice is altered and may be involved in the occurrence of anxiety- and depression-like behaviors.

### 3.3 CP-induced gut microbiota dysbiosis promotes the occurrence of anxiety- and depression-like behaviors in mice

**Figure 3:**
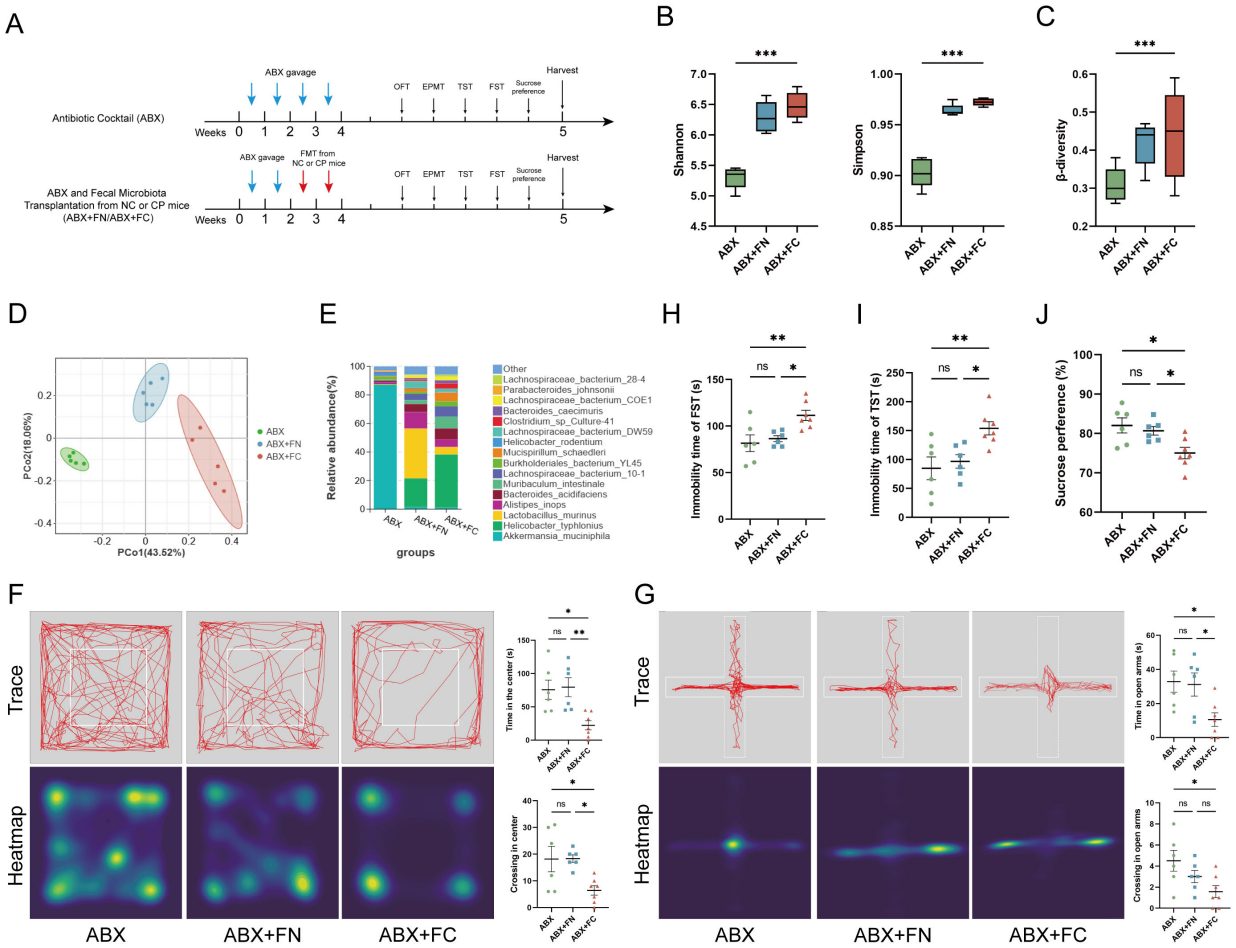
CP-induced gut microbiota dysbiosis promotes the occurrence of anxiety- and depression-like behaviors in mice. **(A)** Experimental design for establishing ABX and FMT treatment mouse model. **(B–D)** Assessment of fecal microbiota by Shannon and Simpson index **(B)**, β-diversity **(C)** and PCoA **(D)** for ABX, ABX+FN and ABX+FC mice. **(E)** Relative abundances of different species in fecal microbiota of mice in three groups. **(F, G)** Representative trajectory traces, heatmap, and statistical analysis of mice in three groups in OFT **(F)** and EPMT **(G)** (n=6-7). **(H–J)** Statistical analysis of immobility time of mice in three groups in FST **(H)** and TST **(I)**, and sucrose consumption ratio **(J)** (n=6-7). ABX, antibiotic cocktail; ABX+FN, antibiotic cocktail and fecal microbiota transplantation from control mice; ABX+FC, antibiotic cocktail and fecal microbiota transplantation from CP mice. *p<0.05; **p<0.01; ***p<0.001.

To further clarify the role of gut microbiota dysbiosis in the development of CP-related anxiety- and depression-like behavior, we pretreated male C57BL/6 mice with ABX and transplanted the fecal microbiota from CP (ABX+FC) or NC (ABX+FN) mice (**Figure 3A**). Compared with mice receiving only ABX treatment, those that underwent FMT presented a decrease in body weight (P < 0.01; **Supplementary Figure S1A**). Nevertheless, both pancreatic weight assessments (**Supplementary Figure S1B**) and histological staining results confirmed that neither ABX intervention nor FMT manipulation triggered CP-associated fibrosis and inflammation alterations in the pancreas of mice (**Supplementary Figures S1C–F**).

16S rRNA sequencing revealed that ABX treatment led to significant depletion of the intestinal microbiota (P<0.001; **Figures 3B, C**), with marked separation in microbiota composition among these three groups (**Figure 3D**). Consistent with the donors, *Lactobacillus murinus* and *Helicobacter typhlonius* were the most abundant bacteria in ABX+FN and ABX+FC mice, respectively (P<0.05; **Figure 3E**; **Supplementary Figures S1G, H**; **Supplementary Table S2**), which indicated that the recipient mice inherited the major microbial characteristics of the donor mice.

Compared with ABX or ABX+FN mice, ABX+FC mice exhibited behavioral abnormalities consistent with those of CP mice. OFT results showed that ABX+FC mice had a significantly shorter center area residence time (22.29 ± 6.90 vs 75.67 ± 14.55 for ABX, P = 0.0147; vs 79.50 ± 14.22 for ABX+FN, P = 0.0092) and fewer center area entries (6.43 ± 1.74 vs 18.17 ± 4.74 for ABX, P = 0.0279; vs 18.33 ± 1.41 for ABX+FN, P = 0.0257) (**Figure 3F**). In EPMT, ABX+FC mice exhibited reduced open arm residence time (10.57 ± 3.99 vs 32.83 ± 6.23 for ABX, P = 0.0309; vs 31.17 ± 6.78 for ABX+FN, P = 0.0468) and fewer open arm entries (1.57 ± 0.57 vs 4.50 ± 0.99 for ABX, P = 0.0274; vs 3.00 ± 0.58 for ABX+FN,P=0.3604) (**Figure 3G**), demonstrating obvious anxiety-like phenotypes in this group.

In addition to anxiety, ABX+FC mice presented significant depression-like behaviors: FST immobility duration was longer in ABX+FC mice (111.66 ± 5.50 vs 81.67 ± 8.92 for ABX, P = 0.0095; vs 86.50 ± 3.24 for ABX+FN, P = 0.0290) (**Figure 3H**); TST immobility time was markedly extended (154.0 ± 11.48 vs 84.83 ± 19.71 for ABX, P = 0.0095; vs 96.83 ± 11.63 for ABX+FN, P = 0.0313) (**Figure 3I**); and sucrose preference was decreased (75.04 ± 1.46 vs 82.06 ± 1.91 for ABX, P = 0.0118; vs 80.71 ± 1.08 for ABX+FN, P = 0.0442) (**Figure 3J**). These results suggest that CP-induced intestinal microbiota dysbiosis is at least partially involved in the occurrence of anxiety- and depression-like behaviors in mice.

### 3.4 CP-induced gut microbiota dysbiosis leads to impaired intestinal barrier function

**Figure 4:**
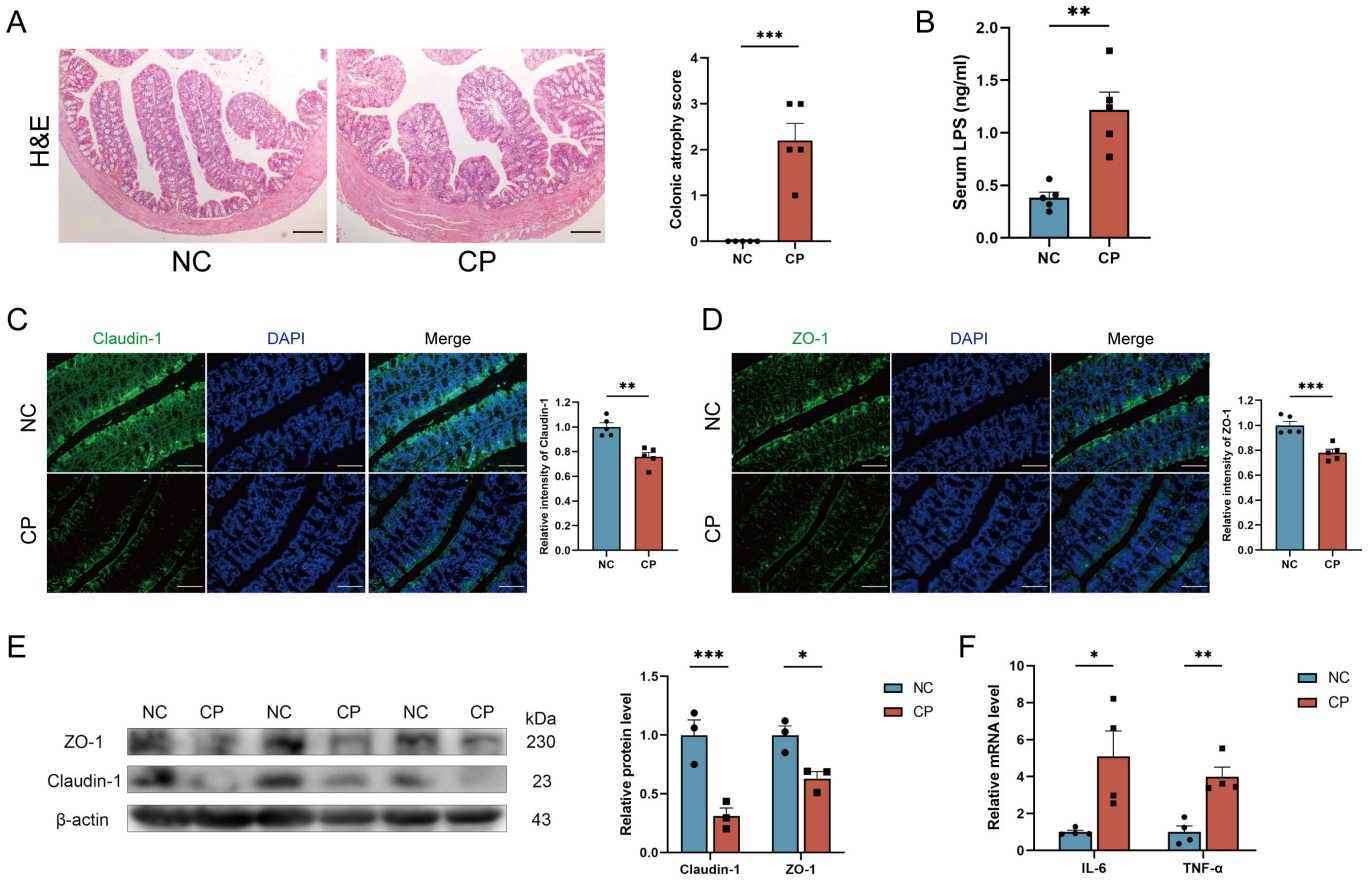
CP-induced gut microbiota dysbiosis leads to impariment of intestinal barrier function. **(A)** Representative H&E staining images of colon and statistic analysis in NC and CP mice. Scale bar, 100 μm. **(B)** Securm LPS concentration of mice in NC and CP mice. **(C, D)** Representative immunofluorescence staining images and statistic analysis of Claudin-1 **(C)** and ZO-1 **(D)** of colon tissue in NC and CP mice. Scale bar, 100 μm. **(E)** Western blot of the Expression of Claudin-1 and ZO-1 in colon tissue in NC and CP mice. **(F)** Relative expression of inflammatory factor IL-6 and TNF-α in colon tissue in NC and CP mice by RT–qPCR. LPS, lipopolysaccharide; RT–qPCR, reverse transcription quantitative polymerase chain reaction. *p<0.05; **p<0.01; ***p<0.001.

To investigate the systemic effects caused by CP-induced gut microbiota dysbiosis, we examined intestinal barrier function. Compared with those of NC mice, the cecum weights of CP mice were significantly greater (P < 0.001; **Supplementary Figure S2A**). Interestingly, this effect was reversed in the ABX+FMT model (P < 0.05; **Supplementary Figure S2B**), which might be attributed to exocrine dysfunction masking the changes in cecum weight induced by the composition of the gut microbiota. However, the intestinal structures of mice in both the CP group (P < 0.001; **Figure 4A**) and the antibiotic-treated plus CP-induced (ABX+FC) group (P < 0.001; **Supplementary Figure S2C**) exhibited significant atrophy, with decreased villus height and partial morphological disorder.

The serum LPS levels in both CP mice (P < 0.01; **Figure 4B**) and ABX+FC mice were significantly increased (P < 0.001; **Supplementary Figure S2D**), suggesting that CP-induced intestinal microbiota dysbiosis could lead to impaired intestinal barrier function. The transmembrane protein claudin-1 and the intracellular adaptor protein ZO-1 are key components of tight junctions and play a central role in maintaining the structure and function of the intestinal barrier and BBB. Immunofluorescence and western blotting confirmed that the levels of Claudin-1 and ZO-1 in the colons of CP mice (P< 0.05; **Figures 3C–E**) and ABX+FC mice (P< 0.05; **Supplementary Figures S2E–G**) were decreased. In addition, the expression of the inflammatory factors IL-6 and TNF-α was upregulated in the colon of CP mice (P< 0.05; **Figure 4F**) and in ABX+FC mice (P< 0.05; **Supplementary Figure S2H**). Together, these results suggest that dysbiosis of the gut microbiota in CP mice serves as a core factor driving intestinal barrier damage and inflammatory responses.

### 3.5 CP-induced gut microbiota dysbiosis leads to HPA axis activation in mice

**Figure 5:**
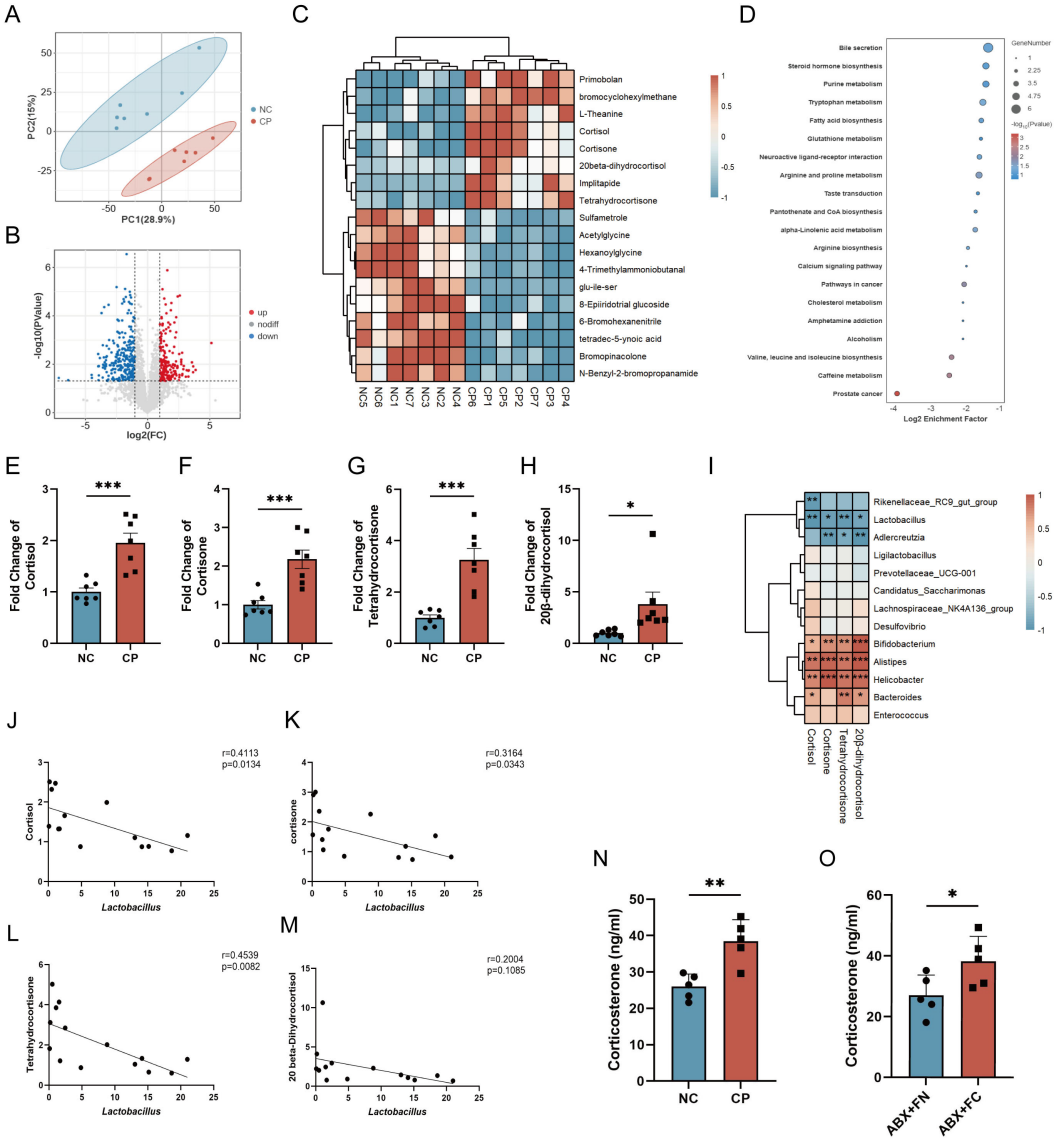
CP leads to differences in serum metabolites. **(A)** PCA showed significant differences in the serum metabolic profiles between CP and NC mice. **(B, C)** Volcano plot **(B)** and heatmap **(C)** showed the differential serum metabolites between CP and NC mice. **(D)** Enrichment analysis of differentially expressed serum metabolites between CP and NC mice. **(E–H)** Relative expression level of cortisol **(E)**, cortisone **(F)**, tetrahydrocortisone **(G)** and 20β-dihydrocortisol **(H)** in CP and NC mice. **(I)** Spearman correlation analysis showing the association between bacteria and the aforementioned serum metabolites. **(J–M)** Linear regression analysis of *Lactobacillus* with cortisol **(J)**, cortisone **(K)**, tetrahydrocortisone **(L)** and 20β-dihydrocortisol **(M)** in the serum of CP and NC mice. **(N, O)** Securm corticosterone concentration of mice in NC and CP mice **(N)**, and in ABX+FN and ABX+FC mice **(O)**. PCA, principal component analysis. *p<0.05; **p<0.01; ***p<0.001.

Gut microbiota dysbiosis and impaired intestinal barrier function may further lead to differences in serum metabolites. We performed untargeted metabolomics in the serum of a CP mouse model. Principal component analysis (PCA) revealed that the serum metabolites of the two groups of mice were significantly different (**Figure 5A**). A total of 448 differentially abundant metabolites were identified (P < 0.05, log_2_FC> 1 or log_2_FC<-1), among which 263 metabolites were downregulated and 185 metabolites were upregulated (**Figure 5B**, **Supplementary Table S3**).

In the CP group, the levels of corticosterone metabolites—including cortisol, cortisone, tetrahydrocortisol, and 20β-dihydrocortisol—were significantly elevated (P<0.05; **Figures 5C, E–H**), accompanied by enrichment of the steroid hormone biosynthesis pathway (**Figure 5D**). In addition, a variety of differential bacteria are correlated with these metabolites, among which *Lactobacillus* shows particularly significant correlations with these metabolites (**Figures 5I–M**). These results strongly suggest that abnormal activation of the HPA axis occurs in CP mice. Therefore, we further detected the serum level of corticosterone—the primary end product of the HPA axis. The results indicated that the serum corticosterone level was significantly increased in both CP and ABX+FC mice (**Figures 5N, O**). These results indicate that CP-induced gut microbiota dysbiosis triggers abnormal activation of the HPA axis, which may lead to the occurrence of anxiety- and depression-like behaviors.

### 3.6 CP-induced gut microbiota dysbiosis induces neuroinflammation and neurotransmitter disorders

**Figure 6:**
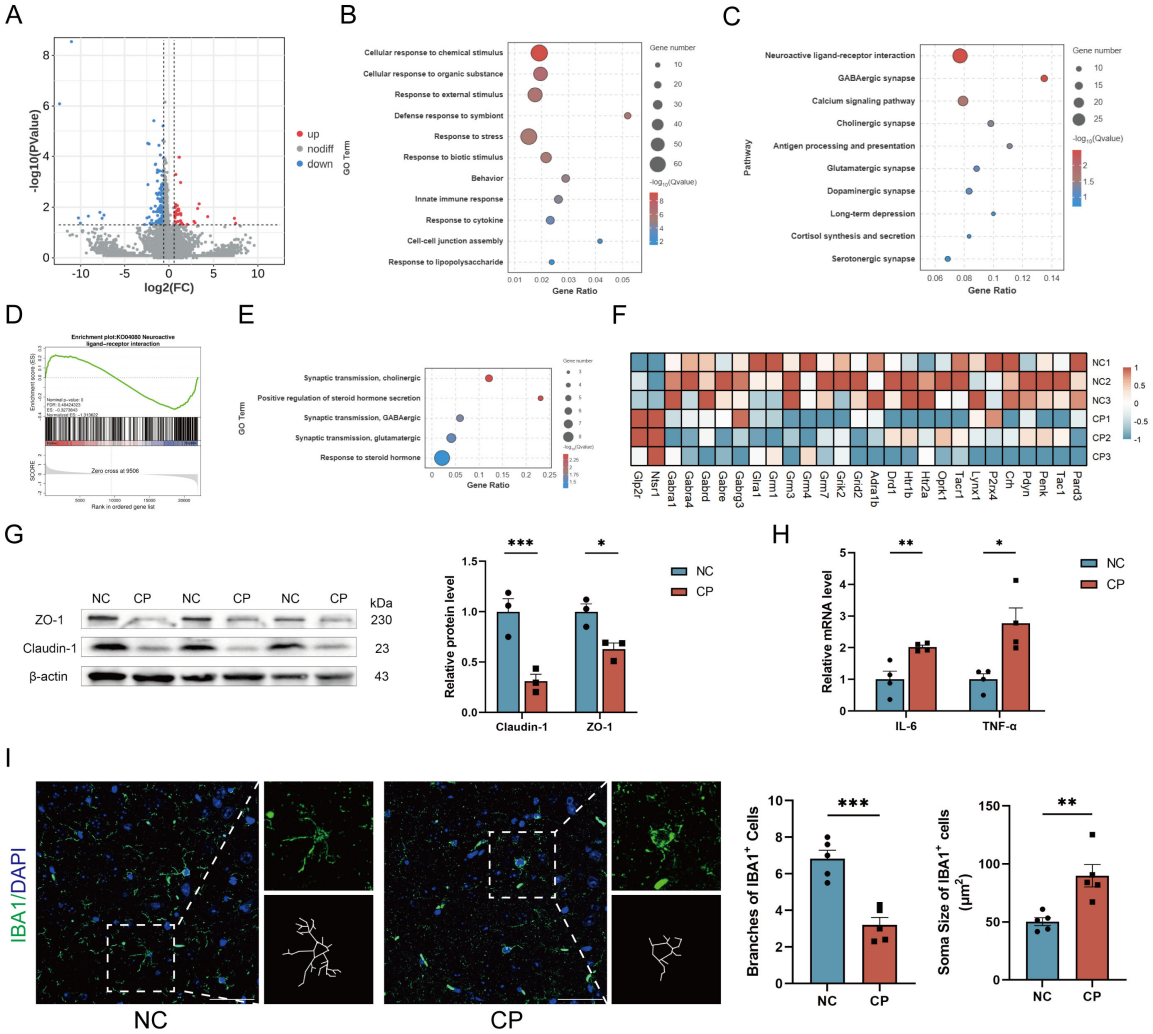
CP induced gut microbiota dysbiosis leads to CNS disorder. **(A)** Volcano plot depicting differentially expressed genes in the cerebral cortex of CP and NC mice. **(B, C)** GO **(B)** and KEGG **(C)** enrichment analysis of differentially expressed genes. **(D)** GSEA of neuroactive ligand–receptor interaction pathways. **(E)** GO enrichment analysis of signal transduction regulation in the nervous and endocrine systems. **(F)** Heatmap of neuroactivate receptor and ligand genes. **(G)** Western blot analysis of Claudin-1 and ZO-1 expression in cerebral cortex of NC and CP mice. **(H)** RT–qPCR quantification of IL-6, TNF-α in brain tissues of NC and CP mice. **(I)** Representative immunofluorescence images of IBA1^+^ cells, with quantification of branch numbers and soma sizes in cerebral cortex of NC and CP mice. Scale bar, 50 μm. BBB, Blood–brain barrier. *p<0.05; **p<0.01; ***p<0.001.

To further investigate how CP promotes the occurrence of anxiety- and depression-like behaviors in mice, we performed transcriptome sequencing on the cerebral cortex of CP and NC mice. Compared with those in the control group, a total of 168 genes were significantly altered in CP mice, including 39 upregulated genes and 129 downregulated genes (P<0.05, FC> 1.5 or FC<-1.5; **Figure 6A**; **Supplementary Table S4**). The GO enrichment of these genes revealed that they are involved in the response to stress, behavior, the immune response, and the response to symbionts and LPS (**Figure 6B**), which suggested that gut microbiota dysbiosis may contribute to the observed behavioral differences in CP mice. GO and KEGG enrichment analyses revealed that CP mice exhibit extensive dysregulation of neuroactive ligand–receptor interactions (**Figures 6C, D**), involving dysregulation of GABA-ergic, glutamatergic, cholinergic, dopaminergic, and serotonergic neurotransmission (**Figures 6C, E, F**). Specifically, the expression of multiple neurotransmitter receptors, particularly the GABAergic receptor subunits *Gabra1*, *Gabra4*, *Gabrg3*, *Gabrd*, and *Gabre* and the metabotropic glutamate receptors *Grm1*, *Grm3*, *Grm4*, and *Grm7*, is downregulated (**Figure 6F**). Additionally, enrichment analysis revealed significant enrichment in the steroid hormone secretion pathway (**Figures 6C, E**), which is consistent with previous results (**Figures 5C–H**).

Indeed, both CP mice and ABX+FC mice exhibited a reduction in BBB function. Specifically, the expression levels of Claudin-1 and ZO-1 were downregulated (P<0.05; **Figure 6G**; **S3A**). Moreover, the levels of the inflammatory factors IL-6 and TNF-α were increased (P<0.05; **Figure 6H**; **S3B**). In addition, we observed morphological changes in the IBA1^+^ microglia of CP mice and mice transplanted with gut microbiota from CP mice, characterized by a decrease in the number of branches and increased soma size (P<0.05; **Figure 6I**; **S3C**), which suggests that the cells are in an activated state. These results indicate that the dysbiosis of the gut microbiota induced by CP could impact the CNS through multiple pathways, including the induction of neurotransmitter disorders and the promotion of neuroinflammation.

### 3.7 Probiotic treatment restores behavioral dysregulation caused by CP

**Figure 7:**
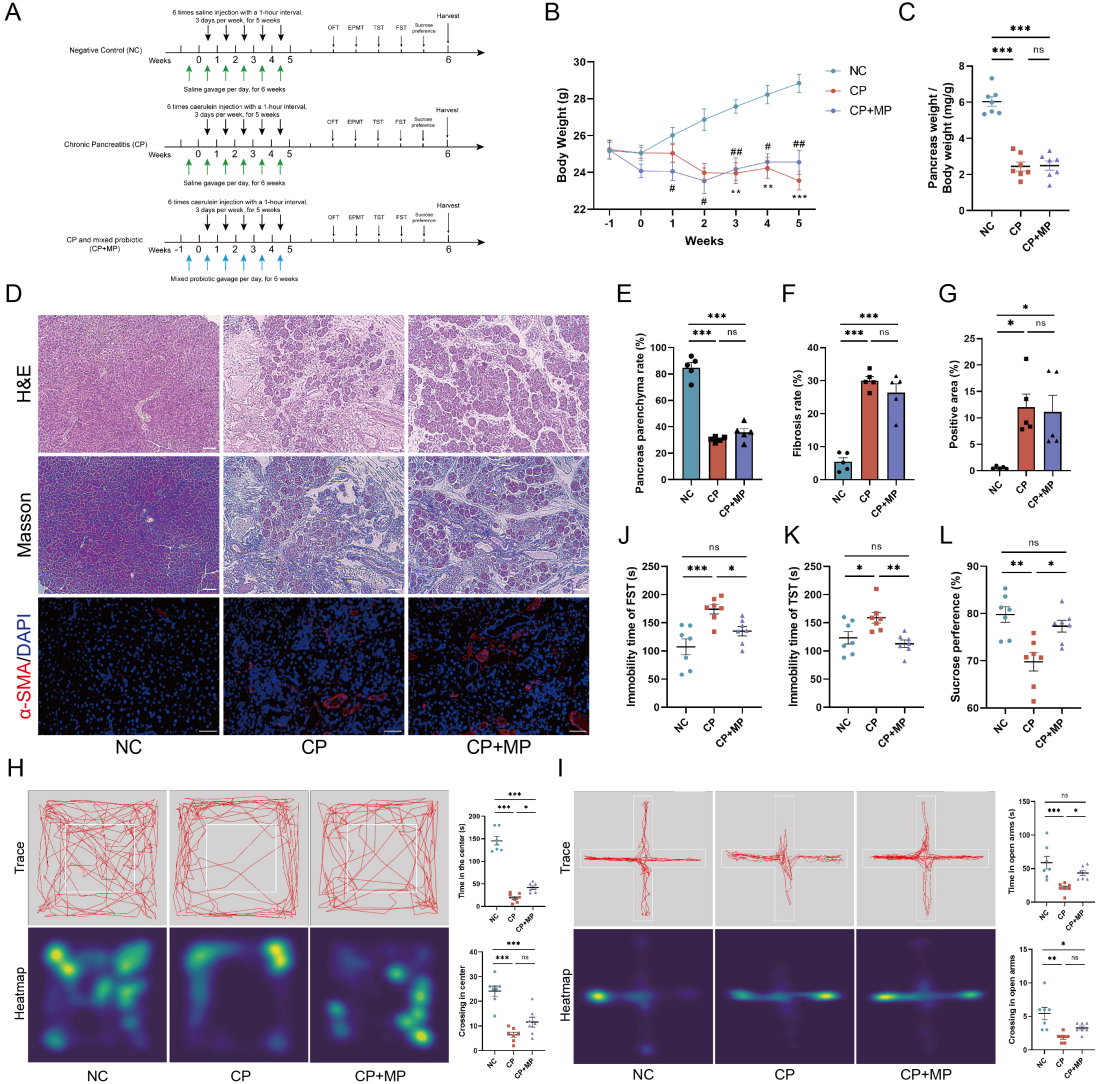
Treatment of MP improves anxiety- and depression-like behaviors in CP mice. **(A)** Experimental design for establishing CP and MP treatment mouse model. **(B, C)** Body weight changes **(B)** and pancreas weight ratio **(C)** of control, CP and CP+MP mice. **(D–G)** Representative H&E, Masson, and α-SMA immunofluorescence staining images of pancreatic tissues from NC, CP and CP+MP mice **(D)**, along with statistical analysis results **(E–G)**. Scale bar, 100 μm. **(H, I)** Representative trajectory traces, heatmap, and statistical analysis of mice in three groups in OFT **(H)** and EPMT **(I)** (n=7). **(J–L)** Statistical analysis of immobility time of mice in three groups in FST **(J)** and TST **(K)**, and sucrose consumption ratio **(L)** (n=7). MP, mixex probiotic. *p<0.05; **p<0.01; ***p<0.001.

Given the pronounced reduction of *Lactobacillus* observed in CP mice, we implemented a therapeutic strategy involving the intragastric administration of an MP consortium composed of multiple strains: *Lactobacillus rhamnosus*, *Lactobacillus helveticus*, *Lactobacillus reuteri*, and *Lactobacillus acidophilus* (**Figure 7A**). Compared with untreated CP mice, CP mice gavaged with MP presented no significant differences in body weight or pancreatic weight (**Figures 7B, C**), nor did MP treatment improve pancreatic fibrosis in these mice (**Figures 7D–G**). However, compared with that of CP mice, the cecum weight of MP-treated mice was significantly lower, suggesting a potential improvement in the intestinal microecology (P<0.01; **Supplementary Figure S4A**). Moreover, MP treatment partially reversed CP-induced colonic villus atrophy (**Supplementary Figure S4B**) and decreased intestinal barrier function (**Supplementary Figures S4C–F**), and reduced the levels of inflammatory factors in the colon (**Supplementary Figure S4G**).

Compared with CP mice, MP-treated mice spent more time (41.86 ± 3.85 vs 19.14 ± 3.76, P = 0.0495) in the central area in the OFT (**Figure 7H**), and in the open arms (43.43 ± 3.62 vs 21.00 ± 2.81, P = 0.0450) of the EPMT (**Figure 7I**). MP treatment also significantly reduced the immobility time of CP mice in the FST (135.1 ± 8.39 vs 174.0 ± 8.10, P = 0.0445) (**Figure 7J**) and TST (112.9 ± 6.54 vs 159.0 ± 9.61, P = 0.0066) (**Figure 7K**) and increased their preference for sucrose solution (77.34 ± 1.28 vs 69.77 ± 1.93, P = 0.0112) (**Figure 7L**). In addition, MP treatment reversed the decrease in BBB function induced by CP (P < 0.05; **Supplementary Figure S5A**) and improved brain inflammation levels, including by reducing the expression of IL-6 and TNF-α (P < 0.05; **Supplementary Figure S5B**) and attenuating the activation of IBA1^+^ microglia (P < 0.01; **Supplementary Figure S5C**). These results indicate that supplementation with MP can effectively reverse CP-induced anxiety- and depression-like behaviors in mice.

## 4 Discussion

In this study, we confirmed that gut microbiota dysbiosis induced by CP leads to anxiety- and depression-like behaviors in mice, which may be mediated by activating the HPA axis, promoting neuroinflammation, and disrupting neurotransmitter transmission in the brain. Through FMT experiments, we verified that gut microbiota dysbiosis acts as an independent driver of these behavioral abnormalities. Additionally, the combined application of multiple probiotics can ameliorate CP-induced anxiety- and depression-like behaviors in mice. These findings underscore the critical role of the gut microbiota in mediating the link between CP and CP-related anxiety and depression via the MGB axis, providing novel mechanistic insights and potential therapeutic targets for addressing psychological comorbidities in patients with CP.

As common psychological comorbidities in CP patients, the incidence rates of anxiety and depression reach 18.2%-46.8% and 23.89%-46.8%, respectively (2, 17–19). These psychological issues not only severely reduce patients’ quality of life but also significantly burden the healthcare system (3, 18). Consistently, we confirmed through the OFT and EPMT that Cae-induced experimental CP mice exhibit anxiety-like behaviors and that, through the FST, TST and SPT, CP mice display depression-like behaviors. These results indicate that the pathological changes induced by CP are not limited to alterations in the digestive system; rather, they can exert a profound reshaping effect on the body’s behavioral patterns.

The MGB axis, as a critical pathway linking the intestinal microbiota and the CNS, mediates the regulation of behavior by the microbiota and has become an important entry point for revealing the mechanism of mind-body interactions (9). Through 16S rRNA sequencing, we observed that CP significantly reshaped the gut microbiota, characterized by an increased abundance of *Bacteroidetes* and *Campylobacterota*. At the genus level, we observed a significant reduction in the abundance of the probiotic *Lactobacillus*, with a notable expansion of the opportunistic pathogen *Helicobacter*. These changes are highly similar to gut microbiota dysbiosis in patients or mice with anxiety or depression (20–24). *Lactobacillus*, a major group of beneficial commensal bacteria, exerts neuroprotective effects by producing GABA and SCFAs, which modulate synaptic plasticity and reduce microglial activation (25–27). Their depletion, as observed in CP (8, 28), may thus weaken this protective buffer, further predisposing individuals to anxiety and depressive symptoms via the MGB axis. Conversely, the overgrowth of *Helicobacter* can damage the integrity of the intestinal barrier, trigger systemic low-grade inflammation, further exacerbate neuroinflammation and disrupt the balance of neurotransmitters in the brain (29–34). Furthermore, through ABX and FMT experiments, we found that recipient mice transplanted with the gut microbiota of CP mice exhibited significant anxiety- and depression-like behaviors without pancreatic pathological changes. These results indicate that the distinctive gut microbiota composition of CP mice is an independent factor contributing to the development of anxiety- and depression-like behaviors via MGB axis.

Impairment of intestinal barrier function not only drives the progression of CP but also serves as a critical link in the systemic effects induced by gut microbiota dysbiosis (28). PICRUSt2 functional prediction analysis of differential bacteria between CP mice and control mice revealed that the differential bacteria in CP mice were significantly enriched in functional pathways such as apoptosis, endocytosis, and glycosaminoglycan degradation, suggesting that such microbial imbalance may directly cause intestinal barrier dysfunction through these functional abnormalities. This conclusion was also verified by experimental results: the expression levels of intestinal barrier proteins ZO-1 and Claudin-1 in the colon of mice in the CP group and ABX+FC group were both significantly downregulated, accompanied by an increase in serum LPS levels.

Intestinal barrier impairment promotes LPS translocation across the damaged epithelium, exacerbating systemic inflammatory responses—a key link in how gut microbiota dysbiosis contributes to anxiety and depression via pathways including immune–inflammatory responses, HPA axis overactivation, and neurotransmitter imbalance (35). Specifically, such LPS translocation induced by microbiota dysbiosis activates the TLR4 signaling pathway, triggers the release of proinflammatory cytokines from microglia and other cells in the brain, and thereby leads to overactivation of the HPA axis (36). Consistent with this, the final metabolic product of the HPA axis corticosterone and its multiple metabolites in the serum of CP mice were significantly increased, and these indicators were strongly negatively correlated with differential genus, especially Lactobacillus. In addition, transcriptome analysis of the brain of CP mice showed that differentially expressed genes were significantly enriched in pathways related to inflammation, the response to LPS and steroid hormone secretion. Furthermore, we also found that both CP mice and mice transplanted with the gut microbiota of CP mice exhibited BBB impairment, increased inflammatory factors, and microglial activation. These results further confirm that gut microbiota dysbiosis plays an important role in inducing inflammatory responses in the CNS and activation of the HPA axis, which may be one of the reasons for the occurrence of anxiety and depression (36, 37).

Under the influence of HPA axis overactivation, dysfunction of the brain’s neurotransmitter system becomes another core link in the pathogenesis of anxiety and depression, and the imbalance between inhibitory pathways dominated by GABA-ergic signals and excitatory circuits centered on glutamatergic signals is precisely the key mechanism by which such dysfunction triggers these disorders (38, 39). Moreover, disruptions in serotonergic, dopaminergic, cholinergic, and noradrenergic neurotransmission collectively exacerbate neurobehavioral abnormalities (40). Consistently, transcriptome sequencing revealed significant dysfunctions in synaptic pathways, such as GABAergic and glutamatergic pathways, in the cerebral cortex, and the expression of related receptors was downregulated. In addition, CP-treated mice also presented significant downregulation of dopamine receptors and 5-HT receptors. These changes suggest that CP may interfere with the signal transmission of neurotransmitters, disrupt the central excitatory–inhibitory balance, and then induce anxiety- and depression-like behaviors.

Finally, we investigated whether supplementation with a mixed probiotic could reverse CP-induced anxiety- and depression-like behaviors in mice. Multiple studies have conclusively demonstrated that bacteria of the genus *Lactobacillus* exert protective effects on intestinal barrier function, inflammatory levels, and HPA axis activation while also inhibiting opportunistic pathogens such as *Helicobacter* (25–27, 29–32). Therefore, we administered a mixture containing four different *Lactobacillus* species via gavage. Although MP treatment did not improve the severity of pancreatic fibrosis, it significantly alleviated anxiety- and depression-like behaviors in mice while reversing intestinal and brain barrier function as well as inflammatory levels in CP mice.

We hypothesize that the restorative effect of MP on the function of the MGB axis in CP mice is attributed to its capacity to modulate the metabolic activity of the gut microbiota, thereby elevating the levels of beneficial metabolites such as SCFAs and GABA, which in turn reconstructs the normal bidirectional regulatory pathway of the MGB axis. The functional *Lactobacillus* strains can promote the fermentation of substrates such as dietary fiber to produce increased amounts of SCFAs. These SCFAs not only enhance intestinal barrier integrity by upregulating the expression of tight junction proteins, thereby reducing the translocation of pro-inflammatory substances like LPS to alleviate peripheral and central neuroinflammation (41–44), but also inhibit the activation of microglia in the brain, reduce the levels of inflammatory factors (45), promote the expression of brain-derived neurotrophic factor (BDNF), and enhance synaptic plasticity (46). Furthermore, *Lactobacillus* can not only synthesize GABA on their own but also secrete signaling molecules to promote GABA synthesis in intestinal mucosal cells. Gut-derived GABA can modulate central GABA levels via the systemic circulation or gut-brain axis pathways (47), thereby affecting the firing frequency of GABAergic neurons in the brain and regulating GABA signaling transduction in key brain regions such as the prefrontal cortex and hippocampus (25, 48).

In conclusion, this study is the first to confirm that CP-induced gut dysbiosis can promote the occurrence of anxiety- and depression-like behaviors in mice by regulating the MGB axis. CP causes alterations in the composition of the intestinal microbiota, impairs intestinal barrier function, and activates the HPA axis via the MGB axis, thereby promoting neuroinflammation and disrupting neurotransmitter transmission in the brain, ultimately inducing anxiety and depressive behaviors. This study not only clarifies the novel mechanism by which CP mediates the development of anxiety and depression through the MGB axis but also provides a potential therapeutic strategy for the clinical application of probiotics to target and regulate the MGB axis in the intervention of such psychological comorbidities.

## Data Availability

The datasets presented in this study can be found in online repositories. The names of the repository/repositories and accession number(s) can be found in the article/**Supplementary Material**.
