# Supplementary material for: Dysregulation of the microbiota-gut-brain axis induced by chronic pancreatitis mediates anxiety- and depression-like behaviors in mice
Source: Front Immunol. 2026 Jan 14;16:1753424. doi: 10.3389/fimmu.2025.1753424 (PMC12847446; doi:10.3389/fimmu.2025.1753424)
Supplement: Supplementary file 1 [file DataSheet1.docx]

Supplementary Material

# Supplementary Figures

## Supplementary Figure 1

**
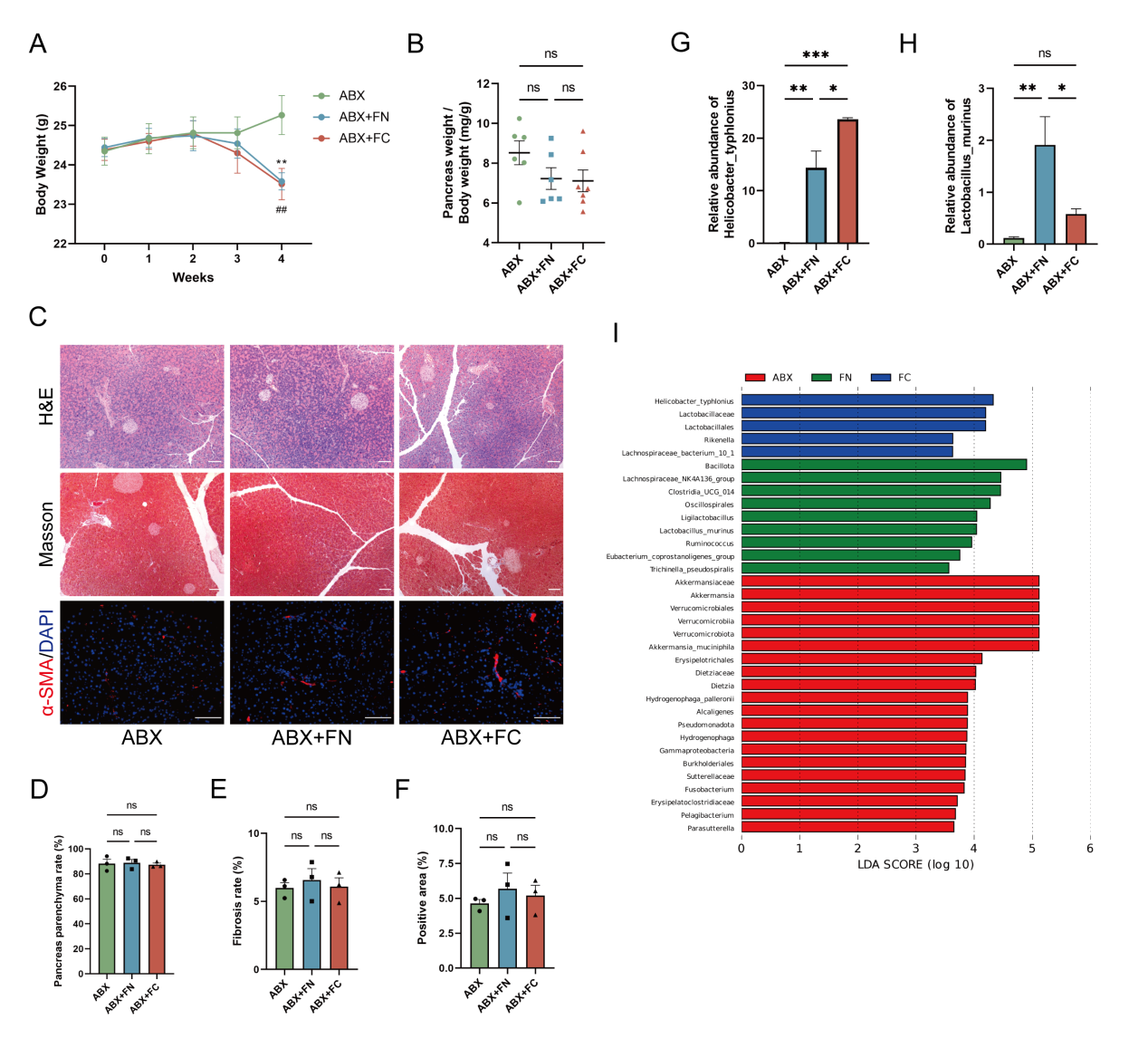
**

**Supplementary Figure 1.** (A-B) Body weight changes (A) and pancreas weight ratio (B) of ABX, ABX+FN and ABX+FC mice. (C-F) Representative H&E, Masson, and α-SMA immunofluorescence staining images of pancreatic tissues from ABX, ABX+FN and ABX+FC mice (C), along with statistical analysis results (D-F). (G-H) Relative abundances of Helicobacter typhlonius (G) and Lactobacillus murinus (H) in fecal microbiota of mice in three groups. (I) LEfSe analysis of differentially abundant microbiota from domain to species levels in fecal samples of mice in three groups. Thresholds: LDA score > 3.5 and P < 0.05. *, p＜0.05; ** and ##, p＜0.01;***, p＜0.001.

## Supplementary Figure 2

**
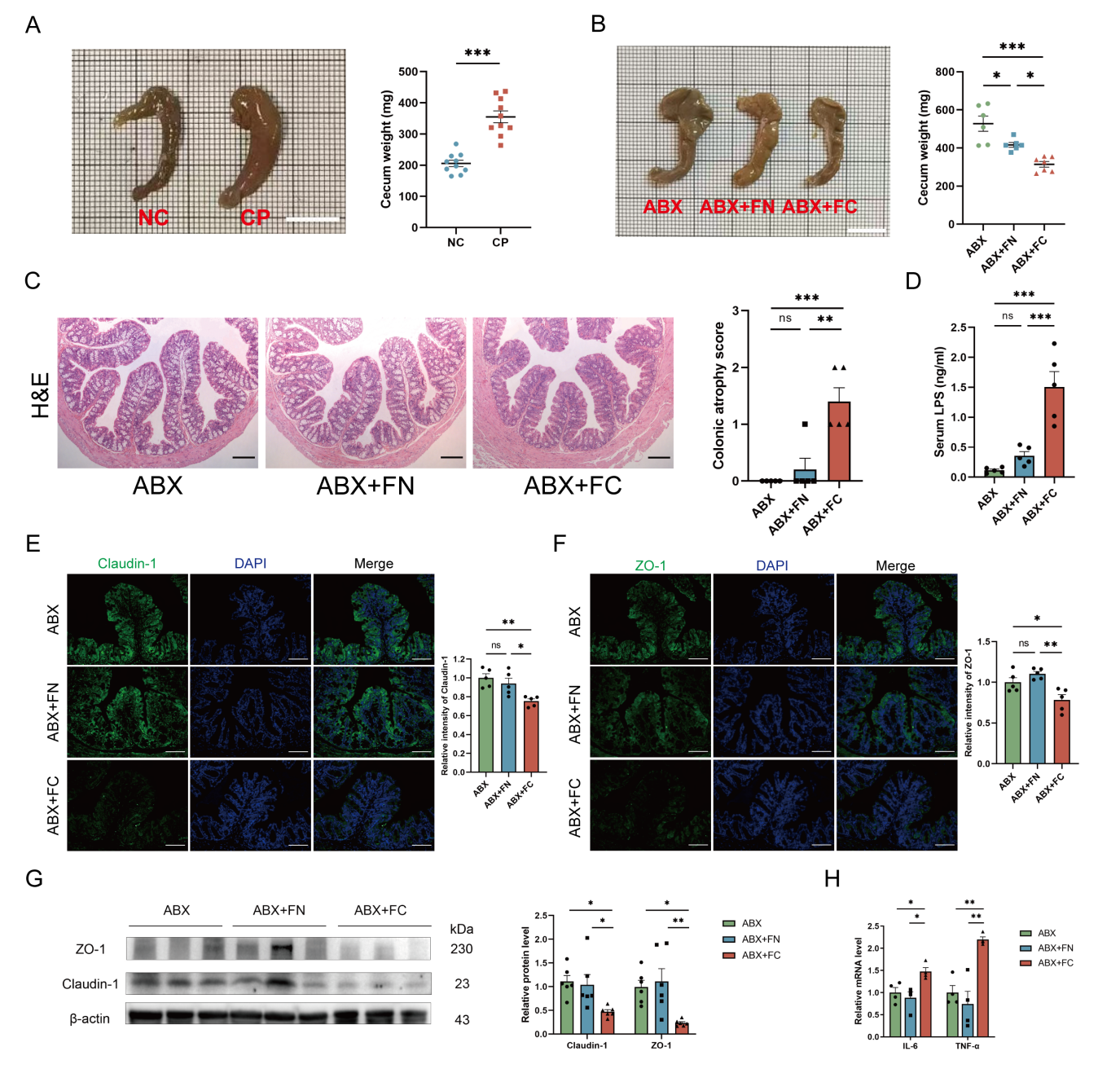
**

**Supplementary Figure 2.** (A) Representative images of cecum in control and CP mice, and the statistic analysis of cecum weight. Scale bar, 1 cm. (B) Representative images of cecum of mice in ABX, ABX+FN and ABX+FC group, and the statistic analysis of cecum weight. Scale bar, 1 cm. (C) Representative H&E staining images of colon and statistic analysis of mice in three group. Scale bar, 100 μm. (D) Securm LPS concentration of mice of mice in three group. (E-F) Representative immunofluorescence staining images and statistic analysis of Claudin-1 (E) and ZO-1 (F) of colon tissue of mice in three group. Scale bar, 100 μm. (G) Western blot of the Expression of Claudin-1 and ZO-1 in colon tissue of mice in three group. (H) Relative expression of inflammatory factor IL-6 and TNF-α in colon tissue of mice in three group by RT-qPCR. *p＜0.05; **p＜0.01; ***p＜0.001.

## Supplementary Figure 3

**
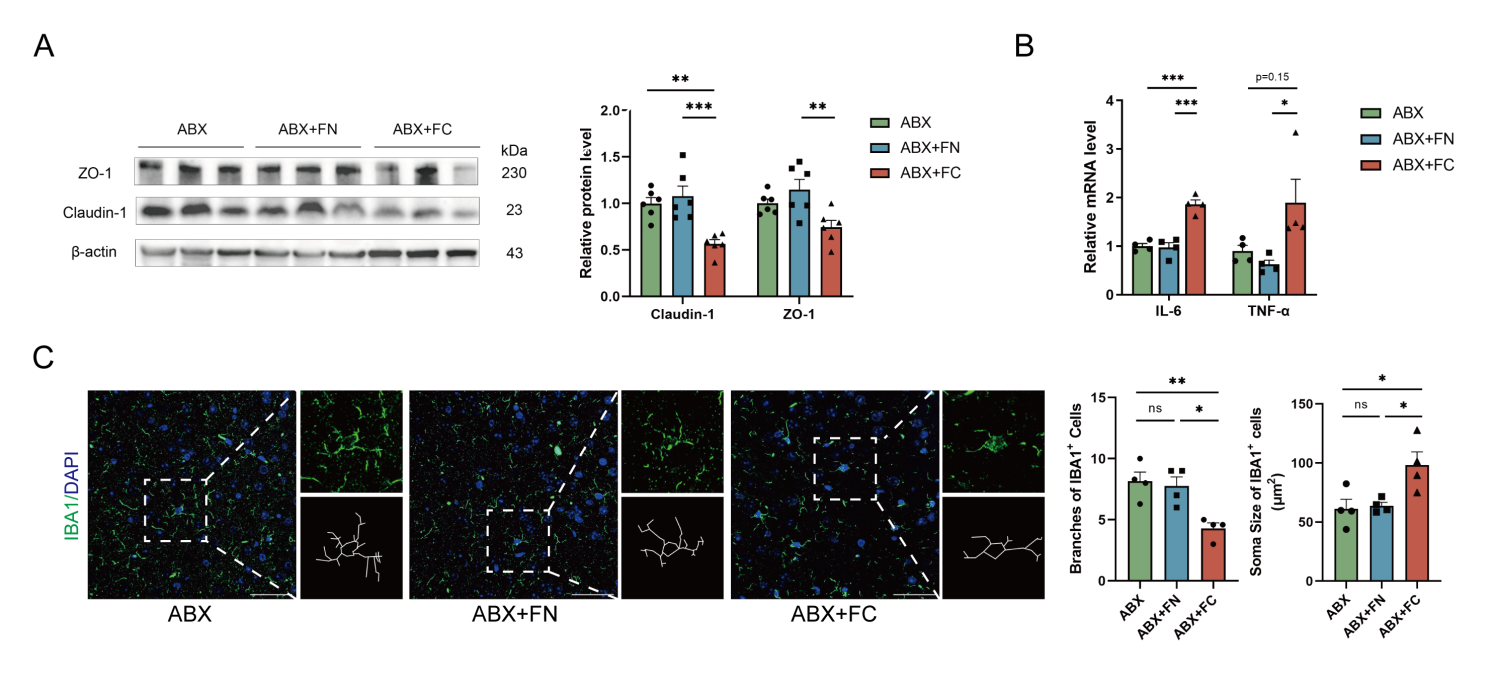
**

**Supplementary Figure 3.** (A) Western blot analysis of Claudin-1 and ZO-1 expression in brain tissues of ABX, ABX+FN and ABX+FC mice. (B) RT-qPCR quantification of IL-6, TNF-α in brain tissues of ABX, ABX+FN and ABX+FC mice. (C) Representative immunofluorescence images of IBA1+ cells, with quantification of branch numbers and soma sizes in brain tissues of ABX, ABX+FN and ABX+FC mice. Scale bar, 50 μm. *p＜0.05; **p＜0.01; ***p＜0.001.

## Supplementary Figure 4

**Supplementary Figure 4.** (A) Representative images of cecum in control, CP and CP+MP mice, and the statistic analysis of cecum weight. (B) Representative H&E staining images of colon and statistic analysis of atrophy score of mice in three group. Scale bar, 50 μm. (C) Securm LPS concentration of mice of mice in three group. (D-E) Representative immunofluorescence staining images and statistic analysis of Claudin-1 (D) and ZO-1 (E) of colon tissue of mice in three group. Scale bar, 100 μm. (F) Western blot of the expression of Claudin-1 and ZO-1 in colon tissue of mice in three group. (G) Relative expression of inflammatory factor IL-6 and TNF-α in colon tissue of mice in three group by RT-qPCR. *p＜0.05; **p＜0.01; ***p＜0.001.

## Supplementary Figure 5


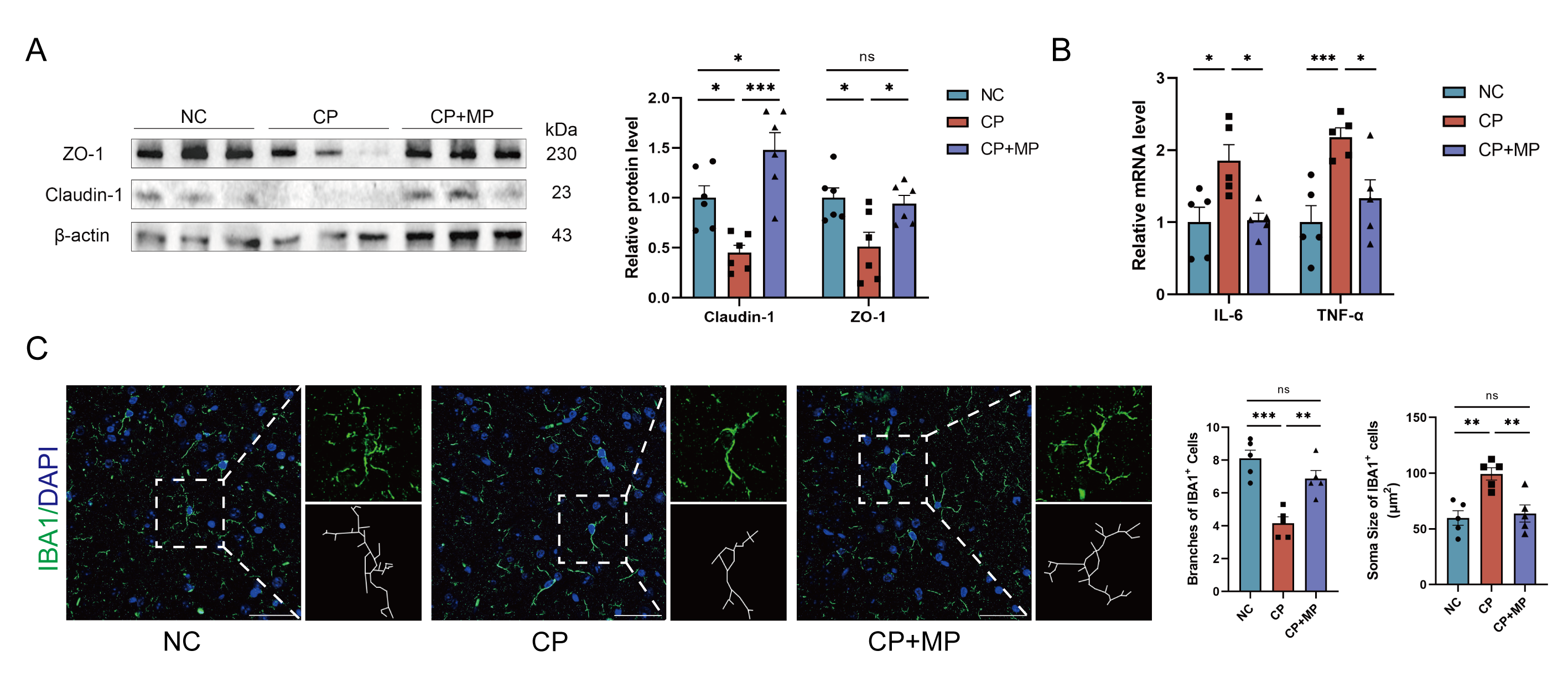


**Supplementary Figure 5.** (A) Western blot analysis of Claudin-1 and ZO-1 expression in brain tissues of control, CP and CP+MP mice. (B) RT-qPCR quantification of IL-6, TNF-α in brain tissues of control, CP and CP+MP mice. (C) Representative immunofluorescence images of IBA1+ cells, with quantification of branch numbers and soma sizes in brain tissues of control, CP and CP+MP mice. Scale bar, 50 μm. *p＜0.05; **p＜0.01; ***p＜0.001.

# Supplementary Figures

## **Supplmentary Table 1**

| **Feature** | **Level** | **Log_hm** | **Class_hm** | **LDA score** | **p value** |
| --- | --- | --- | --- | --- | --- |
| Bacteria.Actinomycetota.Coriobacteriia.Coriobacteriales.Eggerthellaceae.Adlercreutzia.Adlercreutzia_caecicola | Species | 2.963727 | NC | 2.587827 | 0.004574 |
| Bacteria.Verrucomicrobiota.Verrucomicrobiia.Verrucomicrobiales.Verrucomicrobiaceae.Roseimicrobium.Roseimicrobium_gellanilyticum | Species | 1.127675 | CP | 2.375208 | 0.027279 |
| Bacteria.Bacillota.Bacilli.Lactobacillales.Aerococcaceae.Facklamia.Facklamia_tabacinasalis | Species | 2.603569 | NC | 2.35813 | 0.003776 |
| Bacteria.Bacillota.Clostridia.Lachnospirales.Lachnospiraceae.Lachnospiraceae_NK4A136_group.Lachnospiraceae | Species | 1.8004 | CP | 2.00613 | 0.001476 |
| Bacteria.Bacillota.Clostridia.Clostridiales.Clostridiaceae.Candidatus_Arthromitus.Candidatus_Arthromitus_sp_SFB_mouse_Japan | Species | 1.641276 | NC | 2.487733 | 0.010656 |
| Bacteria.Bacillota.Clostridia.Peptostreptococcales_Tissierellales.Family_XI.Anaerococcus.Anaerococcus_prevotii_DSM_20548 | Species | 2.040455 | CP | 2.061925 | 0.000331 |
| Bacteria.Pseudomonadota.Alphaproteobacteria.Rickettsiales.Mitochondria.Incertae_Sedis.Oryza_meyeriana | Species | 1.302524 | CP | 2.304801 | 0.010656 |
| Bacteria.Actinomycetota.Coriobacteriia.Coriobacteriales.Eggerthellaceae.Adlercreutzia.Adlercreutzia_equolifaciens_DSM_19450 | Species | 3.099845 | NC | 2.735309 | 0.003253 |
| Bacteria.Bacteroidota.Bacteroidia.Bacteroidales.Prevotellaceae.Xylanibacter.Prevotella_sp_BP1_145 | Species | 1.391222 | CP | 2.184919 | 0.037243 |
| Bacteria.Bacillota.Clostridia.Lachnospirales.Lachnospiraceae.Ruminococcus_gnavus_group.Ruminococcus_gnavus | Species | 1.096481 | CP | 2.177242 | 0.027279 |
| Bacteria.Bacillota.Bacilli.Lactobacillales.Lactobacillaceae.Weissella.Weissella_paramesenteroides | Species | 3.205654 | CP | 2.957938 | 0.003776 |
| Bacteria.Bacillota.Negativicutes.Acidaminococcales.Acidaminococcaceae.Phascolarctobacterium.Phascolarctobacterium_faecium | Species | 2.403662 | CP | 2.129454 | 0.000331 |
| Bacteria.Bacillota.Bacilli.Exiguobacterales.Exiguobacteraceae.Exiguobacterium.Exiguobacterium_sp_AT1b | Species | 2.154326 | CP | 2.103023 | 0.003776 |
| Bacteria.Pseudomonadota.Gammaproteobacteria.Lysobacterales.Lysobacteraceae.Lysobacter.Lysobacter_soli | Species | 1.127023 | CP | 2.447343 | 0.010656 |
| Bacteria.Bacillota.Clostridia.Lachnospirales.Lachnospiraceae.Roseburia.Roseburia_inulinivorans | Species | 2.402479 | CP | 2.277412 | 0.027279 |
| Bacteria.Actinomycetota.Actinobacteria.Mycobacteriales.Corynebacteriaceae.Corynebacterium.Corynebacterium_tuberculostearicum | Species | 2.718804 | CP | 2.437332 | 0.000451 |
| Bacteria.Bacteroidota.Bacteroidia.Bacteroidales.Rikenellaceae.Alistipes.Alistipes_sp_cv1 | Species | 2.517013 | NC | 2.298717 | 0.042826 |
| Bacteria.Bacillota.Bacilli.Erysipelotrichales.Erysipelotrichaceae.Ileibacterium.Ileibacterium_valens | Species | 3.538139 | CP | 3.245371 | 0.004574 |
| Bacteria.Bacillota.Clostridia.Lachnospirales.Lachnospiraceae.Marvinbryantia.Clostridiales_bacterium_CIEAF_012 | Species | 2.300027 | NC | 2.145124 | 0.022922 |
| Bacteria.Pseudomonadota.Gammaproteobacteria.Pseudomonadales.Pseudomonadaceae.Pseudomonas.Pseudomonas_flexibilis | Species | 2.029655 | CP | 2.10115 | 0.000677 |
| Bacteria.Bacillota.Negativicutes.Veillonellales_Selenomonadales.Veillonellaceae.Veillonella.Veillonella_parvula | Species | 2.947054 | CP | 2.701302 | 0.000331 |
| Bacteria.Bacillota.Bacilli.Erysipelotrichales.Erysipelatoclostridiaceae.Thomasclavelia.Clostridium_spiroforme | Species | 1.392347 | CP | 2.218025 | 0.001194 |
| Bacteria.Bacillota.Bacilli.Erysipelotrichales.Erysipelatoclostridiaceae.Thomasclavelia.Erysipelatoclostridium_ramosum | Species | 2.33809 | CP | 2.095407 | 0.000331 |
| Bacteria.Actinomycetota.Actinobacteria.Actinomycetales.Actinomycetaceae.Schaalia.Schaalia_odontolytica | Species | 3.118959 | CP | 2.946334 | 0.010656 |
| Bacteria.Bacillota.Clostridia.Peptostreptococcales_Tissierellales.Peptostreptococcaceae.Romboutsia.Romboutsia_ilealis | Species | 3.133118 | CP | 2.934857 | 0.007022 |
| Bacteria.Bacillota.Negativicutes.Acidaminococcales.Acidaminococcaceae.Succiniclasticum.Succiniclasticum_ruminis | Species | 2.137311 | CP | 2.212649 | 0.001765 |
| Bacteria.Bacteroidota.Bacteroidia.Bacteroidales.Tannerellaceae.Parabacteroides.Parabacteroides_distasonis | Species | 3.054052 | CP | 2.708436 | 0.00071 |
| Bacteria.Actinomycetota.Actinobacteria.Bifidobacteriales.Bifidobacteriaceae.Bifidobacterium.Bifidobacterium_pseudolongum | Species | 4.910126 | CP | 4.61502 | 0.001131 |
| Bacteria.Campylobacterota.Campylobacteria.Campylobacterales.Helicobacteraceae.Helicobacter.Helicobacter_hepaticus | Species | 3.255099 | CP | 3.009556 | 0.000331 |
| Bacteria.Bacillota.Bacilli.Lactobacillales.Lactobacillaceae.Lactobacillus.Lactobacillus_intestinalis | Species | 4.220129 | NC | 3.970689 | 0.00071 |
| Bacteria.Bacillota.Bacilli.Lactobacillales.Aerococcaceae.Aerococcus.Aerococcus_urinaeequi | Species | 3.978207 | NC | 3.686998 | 0.002838 |
| Bacteria.Bacteroidota.Bacteroidia.Bacteroidales.Bacteroidaceae.Bacteroides.Bacteroides_stercoris_ATCC_43183 | Species | 2.902443 | CP | 2.593719 | 0.027279 |
| Bacteria.Bacillota.Clostridia.Oscillospirales.Oscillospiraceae.Saccharofermentans.Clostridiales_bacterium_Firm_14 | Species | 1.718763 | CP | 2.15755 | 0.007774 |
| Bacteria.Pseudomonadota.Gammaproteobacteria.Pseudomonadales.Moraxellaceae.Acinetobacter.Acinetobacter_sp_ACNIH1 | Species | 2.665646 | CP | 2.338585 | 0.00071 |
| Bacteria.Bacillota.Clostridia.Oscillospirales.Ruminococcaceae.Faecalibacterium.Faecalibacterium_prausnitzii | Species | 2.892277 | CP | 2.592531 | 0.000331 |
| Bacteria.Bacteroidota.Bacteroidia.Bacteroidales.Rikenellaceae.Rikenellaceae_RC9_gut_group.Bacteroidales_bacterium_RM8 | Species | 2.432989 | CP | 2.232586 | 0.026294 |
| Bacteria.Bacillota.Clostridia.Lachnospirales.Lachnospiraceae.Mediterraneibacter.Ruminococcus_torques_ATCC_27756 | Species | 2.503748 | CP | 2.283026 | 0.025579 |
| Bacteria.Campylobacterota.Campylobacteria.Campylobacterales.Helicobacteraceae.Helicobacter.Helicobacter_typhlonius | Species | 4.469835 | CP | 4.152636 | 0.000647 |
| Bacteria.Bacteroidota.Bacteroidia.Bacteroidales.Bacteroidaceae.Bacteroides.Bacteroides_acidifaciens | Species | 3.887434 | CP | 3.391735 | 0.020863 |
| Bacteria.Pseudomonadota.Gammaproteobacteria.Burkholderiales.Sutterellaceae.Parasutterella.Parasutterella_excrementihominis | Species | 2.229062 | CP | 2.059718 | 0.001039 |
| Bacteria.Pseudomonadota.Gammaproteobacteria.Burkholderiales.Comamonadaceae.Comamonas.Comamonas_testosteroni | Species | 3.645475 | NC | 3.329744 | 0.000778 |
| Bacteria.Bacillota.Clostridia.Clostridiales.Clost | Species | 2.782142 | CP | 2.36697 | 0.011657 |
| Bacteria.Planctomycetota | Phylum | 3.209091 | CP | 2.86891 | 0.001503 |
| Bacteria.Myxococcota | Phylum | 2.305229 | CP | 2.052409 | 0.000331 |
| Bacteria.Bdellovibrionota | Phylum | 1.338187 | CP | 2.158194 | 0.027279 |
| Bacteria.Gemmatimonadota | Phylum | 2.452619 | CP | 2.169221 | 0.000331 |
| Bacteria.Acidobacteriota | Phylum | 3.051149 | CP | 2.749302 | 0.000559 |
| Bacteria.Chloroflexota | Phylum | 2.593618 | CP | 2.304325 | 0.000559 |
| Bacteria.Campylobacterota | Phylum | 4.499107 | CP | 4.186276 | 0.000647 |
| Bacteria.Bacillota.Bacilli.Exiguobacterales | Order | 2.154326 | CP | 2.103165 | 0.003776 |
| Bacteria.Planctomycetota.Phycisphaerae.Phycisphaerales | Order | 2.379148 | CP | 2.097186 | 0.000331 |
| Bacteria.Bacillota.Negativicutes.Veillonellales_Selenomonadales | Order | 3.715681 | CP | 3.377333 | 0.008603 |
| Bacteria.Acidobacteriota.Vicinamibacteria.Vicinamibacterales | Order | 2.787784 | CP | 2.493287 | 0.000451 |
| Bacteria.Pseudomonadota.Alphaproteobacteria.Sphingomonadales | Order | 2.369714 | CP | 2.125377 | 0.001194 |
| Bacteria.Actinomycetota.Actinobacteria.Bifidobacteriales | Order | 4.914135 | CP | 4.619283 | 0.001131 |
| Bacteria.Actinomycetota.Coriobacteriia.Coriobacteriales | Order | 4.667965 | NC | 4.219747 | 0.011719 |
| Bacteria.Bacteroidota.Bacteroidia.Cytophagales | Order | 2.462195 | CP | 2.17611 | 0.000331 |
| Bacteria.Pseudomonadota.Alphaproteobacteria.Rickettsiales | Order | 1.302524 | CP | 2.303757 | 0.010656 |
| Bacteria.Bacillota.Clostridia.Clostridia_UCG_014 | Order | 4.350137 | NC | 3.90978 | 0.045999 |
| Bacteria.Planctomycetota.OM190.Incertae_Sedis | Order | 1.497655 | CP | 2.182322 | 0.010656 |
| Bacteria.Bacillota.Clostridia.Clostridiales | Order | 3.161698 | CP | 2.733009 | 0.008652 |
| Bacteria.Actinomycetota.Acidimicrobiia.Microtrichales | Order | 2.261299 | CP | 2.018378 | 0.000331 |
| Bacteria.Campylobacterota.Campylobacteria.Campylobacterales | Order | 4.499107 | CP | 4.186278 | 0.000647 |
| Bacteria.Bacteroidota.Bacteroidia.Chitinophagales | Order | 2.411589 | CP | 2.110613 | 0.005944 |
| Bacteria.Verrucomicrobiota.Verrucomicrobiia.Pedosphaerales | Order | 2.633418 | CP | 2.338242 | 0.000331 |
| Bacteria.Bacillota.Clostridia.Eubacteriales | Order | 1.94207 | NC | 2.08922 | 0.010896 |
| Bacteria.Bacillota.Bacilli.Staphylococcales | Order | 4.359848 | NC | 4.015606 | 0.002322 |
| Bacteria.Bacillota.Clostridia.Monoglobales | Order | 3.120238 | NC | 2.795242 | 0.001131 |
| Bacteria.Planctomycetota.Planctomycetes.Gemmatales | Order | 2.508966 | CP | 2.233419 | 0.000331 |
| Bacteria.Chloroflexota.Dehalococcoidia.S085 | Order | 1.188933 | CP | 2.257245 | 0.010656 |
| Bacteria.Planctomycetota.Phycisphaerae.Tepidisphaerales | Order | 2.555091 | CP | 2.268803 | 0.001194 |
| Bacteria.Actinomycetota.Actinobacteria.Actinomycetales | Order | 3.149285 | CP | 2.976092 | 0.007774 |
| Bacteria.Bacillota.Bacilli.Lactobacillales.Lactobacillaceae.Leuconostoc | Genus | 2.71383 | CP | 2.554219 | 0.010656 |
| Bacteria.Pseudomonadota.Gammaproteobacteria.Enterobacterales.Morganellaceae.Proteus | Genus | 4.856815 | NC | 4.546763 | 0.000778 |
| Bacteria.Bacillota.Bacilli.Bacillales.Planococcaceae.Solibacillus | Genus | 3.170169 | NC | 2.856915 | 0.04584 |
| Bacteria.Bacillota.Bacilli.Erysipelotrichales.Erysipelotrichaceae.Ileibacterium | Genus | 3.538139 | CP | 3.245371 | 0.004574 |
| Bacteria.Verrucomicrobiota.Verrucomicrobiia.Pedosphaerales.Pedosphaeraceae.RS25G | Genus | 1.22965 | CP | 2.188182 | 0.027279 |
| Bacteria.Actinomycetota.Actinobacteria.Bifidobacteriales.Bifidobacteriaceae.Bifidobacterium | Genus | 4.912774 | CP | 4.617783 | 0.001131 |
| Bacteria.Bacillota.Clostridia.Lachnospirales.Lachnospiraceae.Mediterraneibacter | Genus | 2.790922 | CP | 2.49307 | 0.007774 |
| Bacteria.Bacillota.Clostridia.Lachnospirales.Lachnospiraceae.Blautia | Genus | 3.037077 | CP | 2.696734 | 0.001039 |
| Bacteria.Actinomycetota.Coriobacteriia.Coriobacteriales.Eggerthellaceae.Adlercreutzia | Genus | 4.358547 | NC | 3.927342 | 0.002322 |
| Bacteria.Bacillota.Bacilli.Erysipelotrichales.Erysipelotrichaceae.Turicibacter | Genus | 2.857477 | CP | 2.625234 | 0.010656 |
| Bacteria.Acidobacteriota.Vicinamibacteria.Vicinamibacterales.Vicinamibacteraceae.Incertae_Sedis | Genus | 2.581531 | CP | 2.299586 | 0.000451 |
| Bacteria.Pseudomonadota.Gammaproteobacteria.Enterobacterales.Alteromonadaceae.Alteromonas | Genus | 2.77092 | CP | 2.527524 | 0.000331 |
| Bacteria.Pseudomonadota.Alphaproteobacteria.Rhodospirillales.Rhodospirillaceae.Incertae_Sedis | Genus | 1.048927 | CP | 2.184501 | 0.027279 |
| Bacteria.Chloroflexota.Anaerolineae.Anaerolineales.Anaerolineaceae.Flexilinea | Genus | 1.677564 | CP | 2.016358 | 0.011684 |
| Bacteria.Bacillota.Clostridia.Lachnospirales.Lachnospiraceae.Roseburia | Genus | 2.529666 | CP | 2.224 | 0.046461 |
| Bacteria.Pseudomonadota.Gammaproteobacteria.Enterobacterales.Pseudoalteromonadaceae.Pseudoalteromonas | Genus | 2.37376 | CP | 2.149349 | 0.034698 |
| Bacteria.Chloroflexota.Dehalococcoidia.S085.Incertae_Sedis.Incertae_Sedis | Genus | 1.188933 | CP | 2.255268 | 0.010656 |
| Bacteria.Actinomycetota.Actinobacteria.Pseudonocardiales.Pseudonocardiaceae.Saccharothrix | Genus | 1.508749 | CP | 2.033888 | 0.003776 |
| Bacteria.Bacteroidota.Bacteroidia.Bacteroidales.F082.Incertae_Sedis | Genus | 2.891795 | CP | 2.496083 | 0.001616 |
| Bacteria.Bacillota.Bacilli.Staphylococcales.Staphylococcaceae.Mammaliicoccus | Genus | 3.969745 | NC | 3.601638 | 0.011657 |
| Bacteria.Fusobacteriota.Fusobacteriia.Fusobacteriales.Fusobacteriaceae.Cetobacterium | Genus | 1.935671 | NC | 2.283948 | 0.046461 |
| Bacteria.Bacillota.Bacilli.Exiguobacterales.Exiguobacteraceae.Exiguobacterium | Genus | 2.154326 | CP | 2.103281 | 0.003776 |
| Bacteria.Bacillota.Negativicutes.Veillonellales_Selenomonadales.Selenomonadaceae.Megamonas | Genus | 3.538949 | CP | 3.226799 | 0.003776 |
| Bacteria.Bacillota.Clostridia.Lachnospirales.Lachnospiraceae.A2 | Genus | 1.922719 | NC | 2.316894 | 0.001194 |
| Bacteria.Bacillota.Bacilli.Erysipelotrichales.Erysipelotrichaceae.Incertae_Sedis | Genus | 2.755143 | NC | 2.120345 | 0.027423 |
| Bacteria.Bacillota.Bacilli.Lactobacillales.Aerococcaceae.Facklamia | Genus | 2.603569 | NC | 2.358128 | 0.003776 |
| Bacteria.Bacillota.Clostridia.Oscillospirales.Oscillospiraceae.Ruminiclostridium | Genus | 1.715669 | NC | 2.251813 | 0.018486 |
| Bacteria.Bacillota.Negativicutes.Veillonellales_Selenomonadales.Veillonellaceae.Veillonella | Genus | 3.076273 | CP | 2.834098 | 0.000331 |
| Bacteria.Campylobacterota.Campylobacteria.Campylobacterales.Helicobacteraceae.Helicobacter | Genus | 4.498875 | CP | 4.186015 | 0.000647 |
| Bacteria.Verrucomicrobiota.Verrucomicrobiia.Pedosphaerales.Pedosphaeraceae.Incertae_Sedis | Genus | 2.396936 | CP | 2.110232 | 0.000331 |
| Bacteria.Bacillota.Clostridia.Oscillospirales.Butyricicoccaceae.Agathobaculum | Genus | 2.234472 | CP | 2.015045 | 0.001194 |
| Bacteria.Bacillota.Bacilli.Erysipelotrichales.Erysipelatoclostridiaceae.Massiliomicrobiota | Genus | 2.524615 | NC | 2.279322 | 0.000331 |
| Bacteria.Pseudomonadota.Gammaproteobacteria.Burkholderiales.SC_I_84.Incertae_Sedis | Genus | 1.177323 | CP | 2.341755 | 0.027279 |
| Bacteria.Bacillota.Clostridia.Clostridia_UCG_014.Incertae_Sedis.Incertae_Sedis | Genus | 4.350137 | NC | 3.90978 | 0.045999 |
| Bacteria.Bacteroidota.Bacteroidia.Sphingobacteriales.envOPS_17.Incertae_Sedis | Genus | 1.334933 | CP | 2.226679 | 0.010656 |
| Bacteria.Bacillota.Bacilli.Lactobacillales.Lactobacillaceae.Limosilactobacillus | Genus | 3.615172 | NC | 3.215933 | 0.011719 |
| Bacteria.Chloroflexota.Chloroflexia.Thermomicrobiales.AKYG1722.Incertae_Sedis | Genus | 1.307551 | CP | 2.184376 | 0.003776 |
| Bacteria.Verrucomicrobiota.Verrucomicrobiia.Verrucomicrobiales.Verrucomicrobiaceae.Roseimicrobium | Genus | 1.127675 | CP | 2.37802 | 0.027279 |
| Bacteria.Bacillota.Bacilli.Lactobacillales.Lactobacillaceae.Lactobacillus | Genus | 4.990576 | NC | 4.591109 | 0.027423 |
| Bacteria.Bacillota.Clostridia.Oscillospirales.Eubacterium_coprostanoligenes_group.Incertae_Sedis | Genus | 3.229988 | CP | 2.888825 | 0.045999 |
| Bacteria.Bacteroidota.Bacteroidia.Bacteroidales.Tannerellaceae.Parabacteroides | Genus | 3.433323 | CP | 3.065059 | 0.001122 |
| Bacteria.Bacillota.Clostridia.Peptostreptococcales_Tissierellales.Peptostreptococcaceae.Romboutsia | Genus | 3.13474 | CP | 2.935974 | 0.005053 |
| Bacteria.Pseudomonadota.Gammaproteobacteria.Enterobacterales.Enterobacteriaceae.Enterobacter | Genus | 3.551886 | CP | 3.250713 | 0.001131 |
| Bacteria.Bacillota.Clostridia.Clostridiales.Clostridiaceae.Clostridium | Genus | 3.161698 | CP | 2.750235 | 0.008603 |
| Bacteria.Bacillota.Bacilli.Staphylococcales.Staphylococcaceae.Jeotgalicoccus | Genus | 2.867257 | NC | 2.561576 | 0.005578 |
| Bacteria.Actinomycetota.Actinobacteria.Bifidobacteriales.Bifidobacteriaceae.Incertae_Sedis | Genus | 2.205085 | CP | 2.05002 | 0.000559 |
| Bacteria.Bacillota.Bacilli.Erysipelotrichales.Erysipelotrichaceae.Allobaculum | Genus | 3.390905 | NC | 3.076392 | 0.000331 |
| Bacteria.Pseudomonadota.Gammaproteobacteria.Burkholderiales.Sutterellaceae.Parasutterella | Genus | 3.841 | CP | 3.35526 | 0.045999 |
| Bacteria.Bacillota.Clostridia.Eubacteriales.Anaerofustaceae.Anaerofustis | Genus | 1.94207 | NC | 2.098962 | 0.003776 |
| Bacteria.Bacillota.Bacilli.Staphylococcales.Staphylococcaceae.Staphylococcus | Genus | 4.095255 | NC | 3.767056 | 0.002322 |
| Bacteria.Bacillota.Clostridia.Monoglobales.Monoglobaceae.Monoglobus | Genus | 3.120238 | NC | 2.795242 | 0.001131 |
| Bacteria.Pseudomonadota.Gammaproteobacteria.Burkholderiales.Comamonadaceae.Ramlibacter | Genus | 1.163832 | CP | 2.206947 | 0.027279 |
| Bacteria.Bacillota.Negativicutes.Acidaminococcales.Acidaminococcaceae.Phascolarctobacterium | Genus | 2.442893 | CP | 2.169189 | 0.000331 |
| Bacteria.Acidobacteriota.Blastocatellia.Blastocatellales.Blastocatellaceae.Stenotrophobacter | Genus | 1.490508 | CP | 2.08929 | 0.010656 |
| Bacteria.Bacillota.Clostridia.Lachnospirales.Lachnospiraceae.Ruminococcus_gnavus_group | Genus | 3.687555 | CP | 3.356935 | 0.002254 |
| Bacteria.Bacillota.Clostridia.Peptostreptococcales_Tissierellales.Family_XI.Anaerococcus | Genus | 2.120402 | CP | 2.063847 | 0.000331 |
| Bacteria.Bacteroidota.Bacteroidia.Chitinophagales.Chitinophagaceae.Chitinophaga | Genus | 1.416163 | CP | 2.148096 | 0.001194 |
| Bacteria.Bacillota.Clostridia.Lachnospirales.Lachnospiraceae.Lachnospiraceae_XPB1014_group | Genus | 1.405991 | CP | 2.114304 | 0.043466 |
| Bacteria.Bacillota.Clostridia.Clostridiales.Clostridiaceae.Candidatus_Arthromitus | Genus | 1.641276 | NC | 2.487733 | 0.010656 |
| Bacteria.Cyanobacteriota.Vampirivibrionia.Gastranaerophilales.Gastranaerophilaceae.Incertae_Sedis | Genus | 2.933348 | NC | 2.563194 | 0.003276 |
| Bacteria.Bacteroidota.Bacteroidia.Bacteroidales.Rikenellaceae.Rikenellaceae_RC9_gut_group | Genus | 4.099185 | NC | 3.680094 | 0.011719 |
| Bacteria.Bacillota.Clostridia.Oscillospirales.Oscillospiraceae.Oscillibacter | Genus | 3.223147 | CP | 2.822774 | 0.045999 |
| Bacteria.Planctomycetota.OM190.Incertae_Sedis.Incertae_Sedis.Incertae_Sedis | Genus | 1.497655 | CP | 2.182172 | 0.010656 |
| Bacteria.Bacillota.Clostridia.Oscillospirales.Ruminococcaceae.Faecalibacterium | Genus | 2.970055 | CP | 2.667956 | 0.000331 |
| Bacteria.Myxococcota.Polyangiia.Polyangiales.BIrii41.Incertae_Sedis | Genus | 1.492309 | CP | 2.291145 | 0.003776 |
| Bacteria.Bacillota.Clostridia.Oscillospirales.Ruminococcaceae.Acutalibacter | Genus | 3.583374 | CP | 3.14434 | 0.045999 |
| Bacteria.Bacillota.Clostridia.Oscillospirales.Oscillospiraceae.Saccharofermentans | Genus | 2.174709 | CP | 2.31749 | 0.044406 |
| Bacteria.Pseudomonadota.Alphaproteobacteria.Rickettsiales.Mitochondria.Incertae_Sedis | Genus | 1.302524 | CP | 2.304067 | 0.010656 |
| Bacteria.Campylobacterota.Campylobacteria.Campylobacterales.Campylobacteraceae.Campylobacter | Genus | 1.227186 | CP | 2.138354 | 0.027279 |
| Bacteria.Planctomycetota.Planctomycetes.Gemmatales.Gemmataceae.Incertae_Sedis | Genus | 2.316482 | CP | 2.080613 | 0.003776 |
| Bacteria.Actinomycetota.Actinobacteria.Actinomycetales.Actinomycetaceae.Schaalia | Genus | 3.121707 | CP | 2.94816 | 0.010656 |
| Bacteria.Bacteroidota.Bacteroidia.Bacteroidales.Incertae_Sedis.Incertae_Sedis | Genus | 1.682664 | CP | 2.048857 | 0.002143 |
| Bacteria.Pseudomonadota.Alphaproteobacteria.Caulobacterales.Hyphomonadaceae.Hirschia | Genus | 1.169602 | CP | 2.042077 | 0.027279 |
| Bacteria.Bacteroidota.Bacteroidia.Cytophagales.Microscillaceae.Ohtaekwangia | Genus | 1.195489 | CP | 2.313034 | 0.027279 |
| Bacteria.Planctomycetota.Phycisphaerae.Phycisphaerales.Phycisphaeraceae.I_8 | Genus | 0.999619 | CP | 2.495355 | 0.027279 |
| Bacteria.Acidobacteriota.Vicinamibacteria.Vicinamibacterales.Incertae_Sedis.Incertae_Sedis | Genus | 2.356065 | CP | 2.110736 | 0.001194 |
| Bacteria.Bacillota.Clostridia.Oscillospirales.Oscillospiraceae.UCG_005 | Genus | 2.895071 | CP | 2.549282 | 0.004545 |
| Bacteria.Pseudomonadota.Gammaproteobacteria.Pseudomonadales.Halomonadaceae.Cobetia | Genus | 1.552912 | CP | 2.096875 | 0.010656 |
| Bacteria.Bacillota.Clostridia.Oscillospirales.Ruminococcaceae.Eubacterium_siraeum_group | Genus | 2.596688 | NC | 2.315496 | 0.008456 |
| Bacteria.Bacillota.Bacilli.Lactobacillales.Carnobacteriaceae.Atopostipes | Genus | 2.583787 | NC | 2.319744 | 0.019016 |
| Bacteria.Bacteroidota.Bacteroidia.Bacteroidales.Prevotellaceae.Incertae_Sedis | Genus | 3.889294 | CP | 3.461156 | 0.045999 |
| Bacteria.Planctomycetota.Phycisphaerae.Tepidisphaerales.WD2101_soil_group.Incertae_Sedis | Genus | 2.51647 | CP | 2.234298 | 0.001194 |
| Bacteria.Verrucomicrobiota.Verrucomicrobiia.Pedosphaerales.Pedosphaeraceae.Ellin516 | Genus | 1.156325 | CP | 2.443038 | 0.003776 |
| Bacteria.Bacillota.Bacilli.Lactobacillales.Aerococcaceae.Aerococcus | Genus | 3.986275 | NC | 3.695248 | 0.002838 |
| Bacteria.Bacteroidota.Bacteroidia.Bacteroidales.Bacteroidaceae.Bacteroides | Genus | 4.077034 | CP | 3.538127 | 0.020863 |
| Bacteria.Bacteroidota.Bacteroidia.Bacteroidales.Rikenellaceae.Alistipes | Genus | 4.745532 | CP | 4.257364 | 0.003276 |
| Bacteria.Pseudomonadota.Gammaproteobacteria.Burkholderiales.Comamonadaceae.Comamonas | Genus | 3.648154 | NC | 3.33215 | 0.000778 |
| Bacteria.Bacillota.Bacilli.Erysipelotrichales.Erysipelatoclostridiaceae.Thomasclavelia | Genus | 2.519379 | CP | 2.251886 | 0.000331 |
| Bacteria.Bacteroidota.Bacteroidia.Bacteroidales.Marinifilaceae.Butyricimonas | Genus | 2.441839 | CP | 2.128914 | 0.011109 |
| Bacteria.Bacillota.Clostridia.Lachnospirales.Lachnospiraceae.Howardella | Genus | 1.661129 | CP | 2.241054 | 0.004366 |
| Bacteria.Bacillota.Negativicutes.Veillonellales_Selenomonadales.Veillonellaceae.Negativicoccus | Genus | 1.417063 | CP | 2.146935 | 0.001194 |
| Bacteria.Bacillota.Clostridia.Lachnospirales.Lachnospiraceae.GCA_900066575 | Genus | 2.424684 | NC | 2.258042 | 0.006129 |
| Bacteria.Bacillota.Clostridia.Oscillospirales.Ruminococcaceae.DTU089 | Genus | 1.350759 | CP | 2.505585 | 0.003776 |
| Bacteria.Pseudomonadota.Alphaproteobacteria.Hyphomicrobiales.Xanthobacteraceae.Bradyrhizobium | Genus | 1.170592 | CP | 2.065258 | 0.027279 |
| Bacteria.Bacillota.Clostridia.Oscillospirales.Oscillospiraceae.Incertae_Sedis | Genus | 2.823819 | CP | 2.389841 | 0.045999 |
| Bacteria.Actinomycetota.Acidimicrobiia.Acidimicrobiales.Acidimicrobiaceae.Incertae_Sedis | Genus | 1.687486 | CP | 2.270437 | 0.011603 |
| Bacteria.Bacillota.Bacilli.Erysipelotrichales.Erysipelotrichaceae.Dubosiella | Genus | 4.430089 | NC | 4.049108 | 0.003276 |
| Bacteria.Bacillota.Bacilli.Lactobacillales.Lactobacillaceae.Weissella | Genus | 3.246861 | CP | 3.001936 | 0.001194 |
| Bacteria.Pseudomonadota.Gammaproteobacteria.Pseudomonadales.Pseudomonadaceae.Pseudomonas | Genus | 2.133876 | CP | 2.158103 | 0.000647 |
| Bacteria.Acidobacteriota.Blastocatellia.Pyrinomonadales.Pyrinomonadaceae.Arenimicrobium | Genus | 1.152179 | CP | 2.271655 | 0.027279 |
| Bacteria.Bacteroidota.Bacteroidia.Bacteroidales.Tannerellaceae | Family | 3.434125 | CP | 3.065901 | 0.001122 |
| Bacteria.Pseudomonadota.Alphaproteobacteria.Sphingomonadales.Sphingomonadaceae | Family | 2.369714 | CP | 2.125397 | 0.001194 |
| Bacteria.Bacteroidota.Bacteroidia.Bacteroidales.Bacteroidaceae | Family | 4.077034 | CP | 3.538127 | 0.020863 |
| Bacteria.Bacillota.Clostridia.Monoglobales.Monoglobaceae | Family | 3.120238 | NC | 2.795242 | 0.001131 |
| Bacteria.Planctomycetota.Planctomycetes.Gemmatales.Gemmataceae | Family | 2.508966 | CP | 2.233398 | 0.000331 |
| Bacteria.Pseudomonadota.Alphaproteobacteria.Rhodospirillales.Rhodospirillaceae | Family | 1.048927 | CP | 2.184282 | 0.027279 |
| Bacteria.Bacteroidota.Bacteroidia.Sphingobacteriales.envOPS_17 | Family | 1.334933 | CP | 2.226705 | 0.010656 |
| Bacteria.Bacillota.Clostridia.Oscillospirales.Butyricicoccaceae | Family | 2.478712 | CP | 2.166402 | 0.001503 |
| Bacteria.Bacillota.Clostridia.Clostridia_UCG_014.Incertae_Sedis | Family | 4.350137 | NC | 3.90978 | 0.045999 |
| Bacteria.Actinomycetota.Coriobacteriia.Coriobacteriales.Eggerthellaceae | Family | 4.412936 | NC | 3.975387 | 0.002322 |
| Bacteria.Bacillota.Clostridia.Peptostreptococcales_Tissierellales.Family_XI | Family | 2.360445 | CP | 2.160145 | 0.000331 |
| Bacteria.Acidobacteriota.Vicinamibacteria.Vicinamibacterales.Vicinamibacteraceae | Family | 2.587078 | CP | 2.304819 | 0.000451 |
| Bacteria.Planctomycetota.Phycisphaerae.Tepidisphaerales.WD2101_soil_group | Family | 2.51647 | CP | 2.234296 | 0.001194 |
| Bacteria.Pseudomonadota.Alphaproteobacteria.Caulobacterales.Hyphomonadaceae | Family | 1.352569 | CP | 2.082945 | 0.010656 |
| Bacteria.Bacillota.Clostridia.Oscillospirales.Eubacterium_coprostanoligenes_group | Family | 3.229988 | CP | 2.888825 | 0.045999 |
| Bacteria.Pseudomonadota.Gammaproteobacteria.Pseudomonadales.Pseudomonadaceae | Family | 2.133876 | CP | 2.153499 | 0.000647 |
| Bacteria.Bacteroidota.Bacteroidia.Cytophagales.Microscillaceae | Family | 2.296641 | CP | 2.019803 | 0.000331 |
| Bacteria.Chloroflexota.Chloroflexia.Thermomicrobiales.AKYG1722 | Family | 1.307551 | CP | 2.184322 | 0.003776 |
| Bacteria.Pseudomonadota.Gammaproteobacteria.Enterobacterales.Morganellaceae | Family | 4.856921 | NC | 4.544485 | 0.003276 |
| Bacteria.Pseudomonadota.Gammaproteobacteria.Enterobacterales.Alteromonadaceae | Family | 2.783932 | CP | 2.537657 | 0.000331 |
| Bacteria.Bacillota.Clostridia.Peptostreptococcales_Tissierellales.Peptostreptococcaceae | Family | 3.139918 | CP | 2.938956 | 0.003597 |
| Bacteria.Bacillota.Clostridia.Clostridiales.Clostridiaceae | Family | 3.161698 | CP | 2.733009 | 0.008652 |
| Bacteria.Bacteroidota.Bacteroidia.Chitinophagales.Chitinophagaceae | Family | 2.362752 | CP | 2.064478 | 0.005944 |
| Bacteria.Actinomycetota.Actinobacteria.Bifidobacteriales.Bifidobacteriaceae | Family | 4.914135 | CP | 4.619283 | 0.001131 |
| Bacteria.Actinomycetota.Actinobacteria.Micrococcales.Microbacteriaceae | Family | 1.275746 | CP | 2.264125 | 0.027279 |
| Bacteria.Pseudomonadota.Alphaproteobacteria.Rickettsiales.Mitochondria | Family | 1.302524 | CP | 2.304136 | 0.010656 |
| Bacteria.Verrucomicrobiota.Verrucomicrobiia.Pedosphaerales.Pedosphaeraceae | Family | 2.633418 | CP | 2.338241 | 0.000331 |
| Bacteria.Bacillota.Clostridia.Eubacteriales.Anaerofustaceae | Family | 1.94207 | NC | 2.098961 | 0.003776 |
| Bacteria.Planctomycetota.Phycisphaerae.Phycisphaerales.Phycisphaeraceae | Family | 2.379148 | CP | 2.09718 | 0.000331 |
| Bacteria.Actinomycetota.Actinobacteria.Actinomycetales.Actinomycetaceae | Family | 3.149285 | CP | 2.976082 | 0.007774 |
| Bacteria.Bacillota.Bacilli.Staphylococcales.Staphylococcaceae | Family | 4.359848 | NC | 4.015606 | 0.002322 |
| Bacteria.Bacillota.Negativicutes.Veillonellales_Selenomonadales.Veillonellaceae | Family | 3.097518 | CP | 2.855272 | 0.000331 |
| Bacteria.Chloroflexota.Dehalococcoidia.S085.Incertae_Sedis | Family | 1.188933 | CP | 2.256444 | 0.010656 |
| Bacteria.Myxococcota.Polyangiia.Polyangiales.BIrii41 | Family | 1.492309 | CP | 2.291079 | 0.003776 |
| Bacteria.Bacillota.Bacilli.Lactobacillales.Aerococcaceae | Family | 4.004324 | NC | 3.713026 | 0.003052 |
| Bacteria.Planctomycetota.OM190.Incertae_Sedis.Incertae_Sedis | Family | 1.497655 | CP | 2.182126 | 0.010656 |
| Bacteria.Pseudomonadota.Gammaproteobacteria.Burkholderiales.Burkholderiaceae | Family | 1.666003 | NC | 2.375767 | 0.025579 |
| Bacteria.Pseudomonadota.Gammaproteobacteria.Burkholderiales.Comamonadaceae | Family | 3.661749 | NC | 3.338196 | 0.001629 |
| Bacteria.Campylobacterota.Campylobacteria.Campylobacterales.Campylobacteraceae | Family | 1.227186 | CP | 2.138556 | 0.027279 |
| Bacteria.Bacillota.Bacilli.Exiguobacterales.Exiguobacteraceae | Family | 2.154326 | CP | 2.1029 | 0.003776 |
| Bacteria.Campylobacterota.Campylobacteria.Campylobacterales.Helicobacteraceae | Family | 4.498875 | CP | 4.186017 | 0.000647 |
| Bacteria.Bacteroidota.Bacteroidia.Bacteroidales.Rikenellaceae | Family | 4.772889 | CP | 4.128893 | 0.008652 |
| Bacteria.Bacteroidota.Bacteroidia.Bacteroidales.Incertae_Sedis | Family | 2.076641 | CP | 2.189159 | 0.002838 |
| Bacteria.Pseudomonadota.Gammaproteobacteria.Enterobacterales.Pseudoalteromonadaceae | Family | 2.394244 | CP | 2.166832 | 0.034698 |
| Bacteria.Pseudomonadota.Gammaproteobacteria.Burkholderiales.SC_I_84 | Family | 1.177323 | CP | 2.342093 | 0.027279 |
| Bacteria.Acidobacteriota.Vicinamibacteria.Vicinamibacterales.Incertae_Sedis | Family | 2.356065 | CP | 2.110757 | 0.001194 |
| Bacteria.Bacteroidota.Bacteroidia.Bacteroidales.F082 | Family | 2.891795 | CP | 2.496083 | 0.001616 |
| Bacteria.Pseudomonadota.Alphaproteobacteria.Caulobacterales.Caulobacteraceae | Family | 2.108519 | NC | 2.033687 | 0.0391 |
| Bacteria.Chloroflexota.Dehalococcoidia | Class | 1.188933 | CP | 2.256456 | 0.010656 |
| Bacteria.Actinomycetota.Actinobacteria | Class | 4.934526 | CP | 4.633206 | 0.001131 |
| Bacteria.Campylobacterota.Campylobacteria | Class | 4.499107 | CP | 4.186273 | 0.000647 |
| Bacteria.Acidobacteriota.Blastocatellia | Class | 2.507292 | CP | 2.232718 | 0.000331 |
| Bacteria.Actinomycetota.Acidimicrobiia | Class | 2.490067 | CP | 2.220617 | 0.000451 |
| Bacteria.Actinomycetota.Coriobacteriia | Class | 4.667965 | NC | 4.219747 | 0.011719 |
| Bacteria.Planctomycetota.OM190 | Class | 1.497655 | CP | 2.182322 | 0.010656 |
| Bacteria.Acidobacteriota.Vicinamibacteria | Class | 2.791242 | CP | 2.497291 | 0.000451 |
| Bacteria.Planctomycetota.Phycisphaerae | Class | 2.805331 | CP | 2.509982 | 0.000331 |
| Bacteria.Planctomycetota.Planctomycetes | Class | 2.946463 | CP | 2.575019 | 0.014956 |

**Supplementary Table 1.** Linear Discriminant Analysis Effect Size (LEfSe)-based analysis of fecal gut microbiota differences between chronic pancreatitis (CP) and negative control (NC) mice.

## Supplementary Table 2

| Feature | Level | Log_hm | Class_hm | LDA score | p value |
| --- | --- | --- | --- | --- | --- |
| Bacteria.Bacillota.Bacilli.Erysipelotrichales.Erysipelatoclostridiaceae.Coprobacillus.Coprobacillus_cateniformis | Species | 2.523645 | ABX | 2.768682 | 0.0013 |
| Bacteria.Bacillota.Clostridia.Lachnospirales.Lachnospiraceae.Lachnospiraceae_NK4A136_group.Lachnospiraceae_bacterium_10_1 | Species | 3.9219 | ABX+FC | 3.628594 | 0.007521 |
| Bacteria.Pseudomonadota.Gammaproteobacteria.Burkholderiales.Sutterellaceae.Turicimonas.Turicimonas_muris | Species | 2.151137 | ABX | 2.782533 | 0.021988 |
| Bacteria.Bacillota.Clostridia.Lachnospirales.Lachnospiraceae.Roseburia.Eubacterium_sp_14_2 | Species | 2.898885 | ABX+FC | 2.721583 | 0.011425 |
| Bacteria.Verrucomicrobiota.Verrucomicrobiia.Verrucomicrobiales.Akkermansiaceae.Akkermansia.Akkermansia_muciniphila_ATCC_BAA_835 | Species | 3.03657 | ABX | 2.847885 | 0.008266 |
| Bacteria.Bacteroidota.Bacteroidia.Bacteroidales.Rikenellaceae.Alistipes.Alistipes_inops | Species | 3.933953 | ABX+FN | 3.330092 | 0.046421 |
| Bacteria.Bacteroidota.Bacteroidia.Bacteroidales.Bacteroidaceae.Bacteroides.Bacteroides_caecimuris | Species | 3.37445 | ABX+FC | 2.923723 | 0.004472 |
| Bacteria.Bacteroidota.Bacteroidia.Bacteroidales.Marinifilaceae.Butyricimonas.Butyricimonas_virosa | Species | 3.031057 | ABX+FC | 2.768413 | 0.005248 |
| Bacteria.Bacillota.Clostridia.Lachnospirales.Lachnospiraceae.Lachnospiraceae_NK4A136_group.Lachnospiraceae_bacterium_COE1 | Species | 3.475464 | ABX+FC | 3.151945 | 0.007456 |
| Bacteria.Campylobacterota.Campylobacteria.Campylobacterales.Helicobacteraceae.Helicobacter.Helicobacter_rodentium | Species | 3.835662 | ABX | 3.456312 | 0.012778 |
| Bacteria.Bacillota.Clostridia.Oscillospirales.Ruminococcaceae.Acutalibacter.Acutalibacter_muris | Species | 2.241088 | ABX+FC | 2.56604 | 0.017647 |
| Bacteria.Bacillota.Clostridia.Oscillospirales.Ruminococcaceae.Anaerotruncus.Anaerotruncus_colihominis_DSM_17241 | Species | 1.732056 | ABX+FC | 3.066431 | 0.022621 |
| Bacteria.Bacillota.Bacilli.Erysipelotrichales.Erysipelatoclostridiaceae.Thomasclavelia.Erysipelatoclostridium_ramosum | Species | 2.841897 | ABX | 2.850777 | 0.005336 |
| Bacteria.Campylobacterota.Campylobacteria.Campylobacterales.Helicobacteraceae.Helicobacter.Helicobacter_sp_WYS_2001 | Species | 2.013823 | ABX+FC | 2.784953 | 0.007202 |
| Bacteria.Pseudomonadota.Gammaproteobacteria.Burkholderiales.Sutterellaceae.Turicimonas.Burkholderiales_bacterium_YL45 | Species | 3.774562 | ABX | 3.390162 | 0.023993 |
| Bacteria.Bacillota.Clostridia.Lachnospirales.Lachnospiraceae.Lachnospiraceae_Unclassified.Clostridium_sp_Culture_41 | Species | 3.64966 | ABX+FC | 3.295847 | 0.011333 |
| Bacteria.Verrucomicrobiota.Verrucomicrobiia.Verrucomicrobiales.Akkermansiaceae.Akkermansia.Akkermansia_muciniphila | Species | 5.422264 | ABX | 5.112864 | 0.008148 |
| Bacteria.Bacillota.Bacilli.Lactobacillales.Lactobacillaceae.Limosilactobacillus.Lactobacillus_sp_C30An8 | Species | 1.546864 | ABX+FC | 2.896961 | 0.03178 |
| Bacteria.Bacillota.Clostridia.Oscillospirales.Ruminococcaceae.Candidatus_Soleaferrea.Ruminococcaceae_bacterium_GD6 | Species | 2.002278 | ABX+FN | 2.96219 | 0.014996 |
| Bacteria.Bacteroidota.Bacteroidia.Bacteroidales.Bacteroidaceae.Bacteroides.Bacteroides_acidifaciens | Species | 3.957005 | ABX+FC | 3.471013 | 0.049292 |
| Bacteria.Bacillota.Clostridia.Lachnospirales.Lachnospiraceae.Blautia.Lachnospiraceae_bacterium_DW59 | Species | 3.546603 | ABX+FN | 3.289998 | 0.013168 |
| Bacteria.Verrucomicrobiota.Verrucomicrobiia.Verrucomicrobiales.Akkermansiaceae.Akkermansia.Akkermansia_sp_KLE1798 | Species | 1.653579 | ABX | 3.24501 | 0.007146 |
| Bacteria.Pseudomonadota.Gammaproteobacteria.Burkholderiales.Comamonadaceae.Hydrogenophaga.Hydrogenophaga_palleronii | Species | 1.11694 | ABX | 3.885527 | 0.03178 |
| Bacteria.Campylobacterota.Campylobacteria.Campylobacterales.Helicobacteraceae.Helicobacter.Helicobacter_typhlonius | Species | 4.635337 | ABX+FC | 4.326492 | 0.007521 |
| Bacteria.Bacillota.Clostridia.Lachnospirales.Lachnospiraceae.Lachnospiraceae_NK4A136_group.Clostridiales_bacterium_CIEAF_020 | Species | 1.517645 | ABX+FC | 3.384254 | 0.0013 |
| Bacteria.Bacteroidota.Bacteroidia.Bacteroidales.Rikenellaceae.Alistipes.Alistipes_shahii | Species | 2.675314 | ABX | 2.645469 | 0.032387 |
| Bacteria.Bacillota.Bacilli.Lactobacillales.Lactobacillaceae.Ligilactobacillus.Lactobacillus_murinus | Species | 4.416239 | ABX+FN | 4.040501 | 0.0185 |
| Bacteria.Bacteroidota.Bacteroidia.Bacteroidales.Rikenellaceae.Rikenella.Rikenella_microfusus_DSM_15922 | Species | 2.79996 | ABX | 2.602687 | 0.020286 |
| Bacteria.Bacteroidota.Bacteroidia.Bacteroidales.Tannerellaceae.Parabacteroides.Parabacteroides_johnsonii | Species | 3.436834 | ABX | 3.053423 | 0.00778 |
| Bacteria.Bacillota.Clostridia.Lachnospirales.Lachnospiraceae.Blautia.Blautia_sp_YL58 | Species | 2.691313 | ABX | 2.692813 | 0.01483 |
| Bacteria.Bacillota.Clostridia.Lachnospirales.Lachnospiraceae.Lachnospiraceae_NK4A136_group.Trichinella_pseudospiralis | Species | 1.607412 | ABX+FN | 3.563701 | 0.007146 |
| Bacteria.Verrucomicrobiota | Phylum | 5.424358 | ABX | 5.115103 | 0.008652 |
| Bacteria.Pseudomonadota | Phylum | 4.258491 | ABX | 3.882649 | 0.005042 |
| Bacteria.Bacillota | Phylum | 5.482664 | ABX+FN | 4.901855 | 0.022148 |
| Bacteria.Verrucomicrobiota.Verrucomicrobiia.Verrucomicrobiales | Order | 5.424358 | ABX | 5.115103 | 0.008652 |
| Bacteria.Bacillota.Bacilli.Erysipelotrichales | Order | 4.455625 | ABX | 4.135154 | 0.018873 |
| Bacteria.Actinomycetota.Actinobacteria.Bifidobacteriales | Order | 2.894959 | ABX+FC | 2.66608 | 0.003037 |
| Bacteria.Bacillota.Clostridia.Peptococcales | Order | 3.410475 | ABX+FC | 3.069861 | 0.008929 |
| Bacteria.Actinomycetota.Coriobacteriia.Coriobacteriales | Order | 3.400009 | ABX+FN | 3.012908 | 0.015452 |
| Bacteria.Bacillota.Bacilli.Lactobacillales | Order | 4.557325 | ABX+FC | 4.196226 | 0.008148 |
| Bacteria.Pseudomonadota.Gammaproteobacteria.Burkholderiales | Order | 4.210601 | ABX | 3.851953 | 0.010781 |
| Bacteria.Bacillota.Clostridia.Clostridia_UCG_014 | Order | 4.837071 | ABX+FN | 4.452451 | 0.017774 |
| Bacteria.Bacteroidota.Bacteroidia.Sphingobacteriales | Order | 1.741813 | ABX | 3.05831 | 0.022941 |
| Bacteria.Bacillota.Clostridia.Oscillospirales | Order | 4.797156 | ABX+FN | 4.275028 | 0.030501 |
| Bacteria.Bacillota.Bacilli.RF39 | Order | 2.802314 | ABX+FN | 2.632332 | 0.007409 |
| Bacteria.Bacillota.Clostridia.Oscillospirales.Ruminococcaceae.Acutalibacter | Genus | 3.48088 | ABX+FN | 3.165349 | 0.012155 |
| Bacteria.Bacillota.Clostridia.Christensenellales.Christensenellaceae.Christensenellaceae_R_7_group | Genus | 3.075046 | ABX+FC | 2.717118 | 0.025476 |
| Bacteria.Bacillota.Clostridia.Oscillospirales.Ruminococcaceae.Ruthenibacterium | Genus | 2.464871 | ABX | 2.884997 | 0.017205 |
| Bacteria.Bacillota.Clostridia.Lachnospirales.Lachnospiraceae.Lachnospiraceae_FCS020_group | Genus | 2.584874 | ABX | 2.694134 | 0.012401 |
| Bacteria.Bacillota.Clostridia.Lachnospirales.Lachnospiraceae.ASF356 | Genus | 2.900626 | ABX+FC | 2.636309 | 0.00477 |
| Bacteria.Bacillota.Clostridia.Oscillospirales.Ruminococcaceae.Anaerotruncus | Genus | 3.134435 | ABX+FC | 2.790171 | 0.009005 |
| Bacteria.Bacillota.Clostridia.Oscillospirales.Ruminococcaceae.Ruminococcus | Genus | 4.241783 | ABX+FN | 3.957099 | 0.023993 |
| Bacteria.Bacillota.Bacilli.Lactobacillales.Carnobacteriaceae.Atopostipes | Genus | 1.543422 | ABX | 3.296899 | 0.022621 |
| Bacteria.Bacillota.Clostridia.Oscillospirales.Oscillospiraceae.Ruminiclostridium | Genus | 2.589717 | ABX+FN | 2.662555 | 0.002468 |
| Bacteria.Pseudomonadota.Gammaproteobacteria.Enterobacterales.Vibrionaceae.Photobacterium | Genus | 2.294819 | ABX+FC | 2.686523 | 0.013241 |
| Bacteria.Bacillota.Clostridia.Oscillospirales.Butyricicoccaceae.UCG_009 | Genus | 2.540385 | ABX+FN | 2.740175 | 0.020651 |
| Bacteria.Actinomycetota.Actinobacteria.Bifidobacteriales.Bifidobacteriaceae.Bifidobacterium | Genus | 2.894959 | ABX+FC | 2.666273 | 0.003037 |
| Bacteria.Bacillota.Bacilli.Erysipelotrichales.Erysipelotrichaceae.Turicibacter | Genus | 1.993551 | ABX+FC | 3.0321 | 0.044804 |
| Bacteria.Actinomycetota.Coriobacteriia.Coriobacteriales.Eggerthellaceae.Adlercreutzia | Genus | 3.200753 | ABX+FC | 2.827182 | 0.035793 |
| Bacteria.Bacillota.Clostridia.Lachnospirales.Lachnospiraceae.Lachnospiraceae_UCG_006 | Genus | 3.634531 | ABX | 3.263671 | 0.022596 |
| Bacteria.Pseudomonadota.Gammaproteobacteria.Burkholderiales.Sutterellaceae.Turicimonas | Genus | 3.785512 | ABX | 3.401528 | 0.023993 |
| Bacteria.Bacillota.Clostridia.Lachnospirales.Lachnospiraceae.Roseburia | Genus | 3.193169 | ABX+FC | 2.844864 | 0.032387 |
| Bacteria.Actinomycetota.Actinobacteria.Mycobacteriales.Dietziaceae.Dietzia | Genus | 1.12827 | ABX | 4.02025 | 0.03178 |
| Bacteria.Verrucomicrobiota.Verrucomicrobiia.Verrucomicrobiales.Akkermansiaceae.Akkermansia | Genus | 5.424358 | ABX | 5.115103 | 0.008652 |
| Bacteria.Bacillota.Bacilli.Erysipelotrichales.Erysipelatoclostridiaceae.Coprobacillus | Genus | 2.533632 | ABX | 2.76006 | 0.0013 |
| Bacteria.Bacillota.Clostridia.Lachnospirales.Defluviitaleaceae.Defluviitaleaceae_UCG_011 | Genus | 2.158815 | ABX+FN | 2.814907 | 0.010518 |
| Bacteria.Actinomycetota.Actinobacteria.Micrococcales.Brevibacteriaceae.Brevibacterium | Genus | 1.76607 | ABX | 2.858376 | 0.044551 |
| Bacteria.Fusobacteriota.Fusobacteriia.Fusobacteriales.Fusobacteriaceae.Fusobacterium | Genus | 1.548782 | ABX | 3.826496 | 0.040285 |
| Bacteria.Bacillota.Bacilli.Erysipelotrichales.Erysipelotrichaceae.Clostridium_innocuum_group | Genus | 3.162231 | ABX | 2.978504 | 0.007256 |
| Bacteria.Bacillota.Bacilli.Lactobacillales.Lactobacillaceae.Ligilactobacillus | Genus | 4.420496 | ABX+FN | 4.043513 | 0.0185 |
| Bacteria.Bacillota.Clostridia.Lachnospirales.Lachnospiraceae.Lachnospiraceae_NK4A136_group | Genus | 4.773924 | ABX+FN | 4.458297 | 0.012778 |
| Bacteria.Bacillota.Clostridia.Lachnospirales.Lachnospiraceae.Blautia | Genus | 3.648118 | ABX+FC | 3.314411 | 0.047835 |
| Bacteria.Bacillota.Bacilli.Erysipelotrichales.Erysipelatoclostridiaceae.Thomasclavelia | Genus | 3.495715 | ABX | 3.221448 | 0.009005 |
| Bacteria.Bacillota.Clostridia.Oscillospirales.Ruminococcaceae.Harryflintia | Genus | 2.346631 | ABX+FC | 2.365808 | 0.014996 |
| Bacteria.Bacteroidota.Bacteroidia.Sphingobacteriales.Sphingobacteriaceae.Sphingobacterium | Genus | 1.594989 | ABX | 3.429157 | 0.028699 |
| Bacteria.Bacillota.Bacilli.Erysipelotrichales.Erysipelotrichaceae.Allobaculum | Genus | 2.772924 | ABX+FN | 2.950049 | 0.002369 |
| Bacteria.Bacillota.Bacilli.Lactobacillales.Streptococcaceae.Streptococcus | Genus | 1.583811 | ABX+FC | 3.054663 | 0.04695 |
| Bacteria.Bacillota.Clostridia.Peptococcales.Peptococcaceae.Peptococcus | Genus | 1.991465 | ABX+FC | 2.564361 | 0.048155 |
| Bacteria.Pseudomonadota.Gammaproteobacteria.Burkholderiales.Alcaligenaceae.Alcaligenes | Genus | 1.522939 | ABX | 3.885042 | 0.03178 |
| Bacteria.Pseudomonadota.Gammaproteobacteria.Burkholderiales.Sutterellaceae.Parasutterella | Genus | 3.988313 | ABX | 3.650371 | 0.006036 |
| Bacteria.Bacillota.Clostridia.Oscillospirales.Ruminococcaceae.Candidatus_Soleaferrea | Genus | 2.002278 | ABX+FN | 2.962221 | 0.014996 |
| Bacteria.Bacillota.Bacilli.Erysipelotrichales.Erysipelotrichaceae.Holdemania | Genus | 2.346775 | ABX | 3.227422 | 0.002369 |
| Bacteria.Actinomycetota.Coriobacteriia.Coriobacteriales.Eggerthellaceae.Gordonibacter | Genus | 2.381584 | ABX+FN | 3.014686 | 0.004015 |
| Bacteria.Bacillota.Clostridia.Lachnospirales.Lachnospiraceae.Eubacterium_ventriosum_group | Genus | 2.44253 | ABX+FN | 2.656614 | 0.008436 |
| Bacteria.Bacillota.Clostridia.Lachnospirales.Lachnospiraceae.Marvinbryantia | Genus | 3.041428 | ABX+FC | 2.779744 | 0.038774 |
| Bacteria.Pseudomonadota.Gammaproteobacteria.Burkholderiales.Comamonadaceae.Hydrogenophaga | Genus | 1.11694 | ABX | 3.874282 | 0.03178 |
| Bacteria.Bacteroidota.Bacteroidia.Bacteroidales.Rikenellaceae.Rikenella | Genus | 4.032587 | ABX+FC | 3.631134 | 0.032387 |
| Bacteria.Bacillota.Clostridia.Lachnospirales.Lachnospiraceae.Faecalimonas | Genus | 1.517908 | ABX+FN | 3.470659 | 0.03178 |
| Bacteria.Pseudomonadota.Alphaproteobacteria.Hyphomicrobiales.Devosiaceae.Pelagibacterium | Genus | 1.320312 | ABX | 3.677143 | 0.03178 |
| Bacteria.Bacillota.Bacilli.Erysipelotrichales.Erysipelatoclostridiaceae | Family | 4.004459 | ABX | 3.708462 | 0.013982 |
| Bacteria.Pseudomonadota.Gammaproteobacteria.Burkholderiales.Sutterellaceae | Family | 4.199674 | ABX | 3.842637 | 0.010781 |
| Bacteria.Bacteroidota.Bacteroidia.Bacteroidales.Marinifilaceae | Family | 3.85925 | ABX+FC | 3.344349 | 0.011796 |
| Bacteria.Bacillota.Clostridia.Oscillospirales.Eubacterium_coprostanoligenes_group | Family | 4.064444 | ABX+FN | 3.752802 | 0.017774 |
| Bacteria.Actinomycetota.Actinobacteria.Bifidobacteriales.Bifidobacteriaceae | Family | 2.894959 | ABX+FC | 2.666199 | 0.003037 |
| Bacteria.Bacteroidota.Bacteroidia.Sphingobacteriales.Sphingobacteriaceae | Family | 1.741813 | ABX | 3.057047 | 0.022941 |
| Bacteria.Bacillota.Bacilli.Lactobacillales.Lactobacillaceae | Family | 4.556595 | ABX+FC | 4.197247 | 0.008148 |
| Bacteria.Actinomycetota.Coriobacteriia.Coriobacteriales.Eggerthellaceae | Family | 3.347413 | ABX+FN | 2.956705 | 0.022596 |
| Bacteria.Bacillota.Clostridia.Lachnospirales.Defluviitaleaceae | Family | 2.158815 | ABX+FN | 2.80833 | 0.010518 |
| Bacteria.Bacillota.Clostridia.Oscillospirales.Butyricicoccaceae | Family | 2.679206 | ABX+FN | 2.661221 | 0.008148 |
| Bacteria.Pseudomonadota.Alphaproteobacteria.Hyphomicrobiales.Rhizobiaceae | Family | 2.020402 | ABX | 2.784727 | 0.041432 |
| Bacteria.Bacillota.Bacilli.Lactobacillales.Streptococcaceae | Family | 1.583811 | ABX+FC | 3.053852 | 0.04695 |
| Bacteria.Actinomycetota.Actinobacteria.Micrococcales.Brevibacteriaceae | Family | 1.76607 | ABX | 2.857956 | 0.044551 |
| Bacteria.Actinomycetota.Coriobacteriia.Coriobacteriales.Atopobiaceae | Family | 2.364634 | ABX+FN | 2.473836 | 0.025767 |
| Bacteria.Fusobacteriota.Fusobacteriia.Fusobacteriales.Fusobacteriaceae | Family | 2.076261 | ABX | 3.187652 | 0.048724 |
| Bacteria.Bacillota.Clostridia.Peptococcales.Peptococcaceae | Family | 3.410475 | ABX+FC | 3.069971 | 0.008929 |
| Bacteria.Pseudomonadota.Gammaproteobacteria.Enterobacterales.Vibrionaceae | Family | 2.536134 | ABX+FC | 2.798679 | 0.025476 |
| Bacteria.Verrucomicrobiota.Verrucomicrobiia.Verrucomicrobiales.Akkermansiaceae | Family | 5.424358 | ABX | 5.115103 | 0.008652 |
| Bacteria.Bacillota.Clostridia.Oscillospirales.UCG_010 | Family | 3.088287 | ABX+FC | 2.830132 | 0.026252 |
| Bacteria.Actinomycetota.Actinobacteria.Mycobacteriales.Dietziaceae | Family | 1.12827 | ABX | 4.028121 | 0.03178 |
| Bacteria.Pseudomonadota.Alphaproteobacteria | Class | 3.012619 | ABX | 2.725032 | 0.024234 |
| Bacteria.Verrucomicrobiota.Verrucomicrobiia | Class | 5.424358 | ABX | 5.115103 | 0.008652 |
| Bacteria.Pseudomonadota.Gammaproteobacteria | Class | 4.233108 | ABX | 3.856481 | 0.007673 |
| Bacteria.Actinomycetota.Coriobacteriia | Class | 3.400009 | ABX+FN | 3.012908 | 0.015452 |
| Bacteria.Actinomycetota.Actinobacteria | Class | 3.265233 | ABX | 2.834155 | 0.045502 |

**Supplementary Table 2.** LEfSe-based analysis of fecal gut microbiota differences in mice: antibiotic cocktail treatment (ABX), ABX followed by fecal microbiota transplantation with chronic pancreatitis mouse-derived gut microbiota (ABX+FC), and ABX followed by fecal microbiota transplantation with negative control mouse-derived gut microbiota (ABX+FN).

## **Supplementary Table 3**

| **Name** | **VIP** | **p_value** | **log2FoldChange** | **Regulation** |
| --- | --- | --- | --- | --- |
| Implitapide | 2.207951495 | 0.001363357 | 5.155126389 | Up |
| NP-020292 | 1.597263492 | 0.017761381 | 3.887423082 | Up |
| ethyl 2-(2-acetyl-3,5-dihydroxyphenyl)acetate | 1.843676837 | 0.012566912 | 3.691771952 | Up |
| Ciprofibrate | 1.970244195 | 0.021925035 | 3.681793457 | Up |
| Carbaryl | 1.969229694 | 0.008595303 | 3.58907839 | Up |
| Isoquinoline | 1.723231798 | 0.018235679 | 3.337138225 | Up |
| LPE 2:0 | 1.727945524 | 0.033146313 | 3.306293868 | Up |
| 2-{(4S,5S,5aS,9aS)-4-Methoxy-6,6,9a-trimethyl-5-[(2E,4E,6E)-2,4,6-octatrienoyloxy]-1-oxo-1,3,4,5,5a,6,7,8,9,9a-decahydro-2H-benzo[E]isoindol-2-yl}pentanedioic acid | 1.915355459 | 0.019118636 | 3.302375122 | Up |
| Indoleacetic acid | 1.675123776 | 0.018140043 | 3.165871208 | Up |
| 6-Methylindole | 1.624245867 | 0.033647199 | 3.098483072 | Up |
| NP-003024 | 1.802997408 | 0.012076174 | 2.931510075 | Up |
| 3-Hydroxy-2-nitro-1H-phenalen-1-one | 1.684144789 | 0.01641088 | 2.89567953 | Up |
| (R)-2,3-Dihydroxy-isovalerate | 1.742986226 | 0.026049157 | 2.820716722 | Up |
| 15-HETE-G | 1.845631825 | 0.029554532 | 2.798816607 | Up |
| N,N-dimethyl-N'-(3-phenyl[1,2,4]triazolo[4,3-b]pyridazin-6-yl)iminoformamide | 1.951126493 | 0.003448645 | 2.798668739 | Up |
| Bilirubin | 1.608492717 | 0.006611268 | 2.734189081 | Up |
| Citrinin | 1.825014948 | 0.010291709 | 2.733951831 | Up |
| (1S,5R,9R,13R)-1,5,9-trimethyl-11,14,15,16-tetraoxatetracyclo [10.3.1.0⁴,¹¹.0⁸,¹¹] hexadecan-10-one | 1.752731404 | 0.008177146 | 2.724848227 | Up |
| Primobolan | 2.072287747 | 1.49277E-05 | 2.612207028 | Up |
| N1-[4-(3-pyrazin-2-yl-4,5-dihydro-1H-1,2,4-triazol-5-yl)phenyl]acetamide | 1.655708864 | 0.028234104 | 2.580671597 | Up |
| NP-016721 | 1.408335266 | 0.035615289 | 2.57725113 | Up |
| 1-(4-Amino-1,2,5-oxadiazol-3-yl)-5-methyl-1H-1,2,3-triazole-4-carboxylic acid | 1.84763725 | 0.003874529 | 2.563464317 | Up |
| Chlorpropamide | 1.478287552 | 0.046784819 | 2.528558012 | Up |
| RS-67,333 | 2.174558993 | 1.62606E-05 | 2.441690421 | Up |
| [3-({3-[(Cyclopropylmethyl)amino]-3-oxetanyl}methyl)-1,2-oxazol-5-yl]methanol | 1.88790197 | 0.013997766 | 2.424529388 | Up |
| 3,3',4,4'-Tetrahydroxy-5,5'-diisopropyl-2,2'-dimethylbiphenyl | 1.848280663 | 0.012182957 | 2.418663994 | Up |
| 3-(1,1,2,3,3,3-hexafluoropropyl)adamantane-1-carboxylic acid | 1.653434617 | 0.014633397 | 2.404614342 | Up |
| 7-(Ethoxycarbonyl)-2,3-dihydrothieno[3,4-b][1,4]dioxine-5-carboxylic acid | 1.613303674 | 0.029860975 | 2.34200513 | Up |
| 1-methyl-3,5-di(1-naphthylmethylidene)piperidin-4-one | 1.965123047 | 0.000490698 | 2.324357984 | Up |
| 3-Ethyl 5-methyl 2-((2-aminoethoxy)methyl)-4-(2-chlorophenyl)-6-methylpyridine-3,5-dicarboxylate | 1.992281472 | 0.000117565 | 2.324135379 | Up |
| Hydroxyphenylacetylglycine | 1.884840343 | 0.00779382 | 2.310378802 | Up |
| Glutamyl-S-(C8H17O)-cysteinylglycine | 1.466837121 | 0.025792358 | 2.310144451 | Up |
| 3-{[3,5-di(trifluoromethyl)anilino]methylidene}pentane-2,4-dione | 1.591466852 | 0.025568383 | 2.270512512 | Up |
| Midodrine | 1.996264335 | 0.000807405 | 2.248417234 | Up |
| 8-cyclopentyl-2-{[1-(ethanesulfonyl)piperidin-4-yl]amino}-6-(methylamino)pteridin-7(8H)-one | 1.32290863 | 0.020383117 | 2.247985744 | Up |
| 1-Hydroxyisoquinoline | 1.666052421 | 0.023772321 | 2.243223568 | Up |
| N'2-(2-furylcarbonyl)-3-chloro-4-methylthiophene-2-carbohydrazide | 1.936881835 | 0.019643054 | 2.239164198 | Up |
| LMST01160025 | 1.732234505 | 0.04006935 | 2.221344971 | Up |
| LysoPI(16:0/0:0) | 1.782073256 | 0.011924528 | 2.211419335 | Up |
| 4-Acetamido-N-({(1S,4S,6S)-6-isopropyl-3-methyl-4-[2-(4-methyl-1-piperazinyl)-2-oxoethyl]-2-cyclohexen-1-yl}methyl)benzamide | 1.771735638 | 0.010863582 | 2.208533103 | Up |
| N-[3-(1,4′-Bipiperidin-1′-yl)propyl]-1-(4,6-dimethyl-1,3-benzothiazol-2-yl)piperidine-4-carboxamide | 2.057843651 | 0.000193974 | 2.177155494 | Up |
| N2'-AcetylgentamicinC1a | 1.851341521 | 0.001950067 | 2.158968573 | Up |
| Cholic acid | 1.59357143 | 0.019742271 | 2.138662989 | Up |
| LMST04030214 | 1.58677607 | 0.027677737 | 2.100905188 | Up |
| Murrangatin | 1.49533228 | 0.014819657 | 2.09320523 | Up |
| 3,12-dihydroxy-4,6a,6b,11,12,14b-hexamethyl-1,2,3,4a,5,6,7,8,9,10,11,12a,14,14a-tetradecahydropicene-4,8a-dicarboxylic acid | 1.751346809 | 0.010341783 | 2.073457697 | Up |
| 5-Hydroxyconiferyl alcohol | 1.506687131 | 0.010819675 | 2.053926432 | Up |
| (4R,5S,6S,7R)-4,7-Dibenzyl-5,6-dihydroxy-1,3-bis[2-(2-methoxyethoxy)ethyl]-1,3-diazepan-2-one | 1.935181464 | 0.003497592 | 2.041340019 | Up |
| Methanetetrayltetrakis(trimethylstannane) | 1.179045916 | 0.03303298 | 2.03784222 | Up |
| methyl 2-(2,6-dihydroxy-4-methylbenzoyl)-3,5-dihydroxybenzoate | 1.790655901 | 0.032274358 | 1.990979677 | Up |
| NCGC00347703-02_C30H44O5_Lup-20(29)-ene-27,28-dioic acid, 3-oxo-, (5xi,9xi,18xi)- | 1.539084477 | 0.030078642 | 1.968134965 | Up |
| 3-(2-Oxo-2,3-dihydro-1,3-benzoxazol-3-yl)propanoic acid | 1.625764945 | 0.013015065 | 1.952616885 | Up |
| Secobarbital | 1.907335758 | 3.03513E-05 | 1.936881235 | Up |
| 5-[1,2,4a-trimethyl-5-(propanoyloxymethyl)-2,3,4,7,8,8a-hexahydronaphthalen-1-yl]-3-methylpentanoic acid | 1.701708482 | 0.022523798 | 1.934268764 | Up |
| 20 beta-Dihydrocortisol | 1.846492754 | 0.033830376 | 1.929479722 | Up |
| 4-[(Morpholin-4-ylcarbothioyl)sulfanyl]morpholine | 1.704494007 | 0.036528564 | 1.897841546 | Up |
| Norselic acid E | 1.281348665 | 0.043412555 | 1.893693626 | Up |
| 5-(4-Fluorophenyl)-2,3,5,6-tetrahydroimidazo[2,1-a]isoquinolin-5-ol | 2.085213495 | 6.03516E-05 | 1.892223225 | Up |
| VIPROSTOL | 1.812653247 | 0.017216166 | 1.851649789 | Up |
| 1H-1,2,4-triazolo[4,3-b][1,2,4]triazole-3,6-diamine | 1.612716586 | 0.000437748 | 1.842647351 | Up |
| (2S)-1-[(2S,3S)-3-Hexyl-4-oxo-2-oxetanyl]-2-tridecanyl N-formylglycinate | 1.575302972 | 0.045955798 | 1.839773125 | Up |
| L-1,2,3,4-Tetrahydro-beta-carboline-3-carboxylic acid | 2.087164426 | 0.000233555 | 1.833698566 | Up |
| N1-[2-(trifluoromethyl)phenyl]-2-[imino(2-pyridyl)methyl]hydrazine-1-carbothioamide | 1.638310344 | 0.011188109 | 1.808816966 | Up |
| 3_5-Dihydroxy-1_4-naphthoquinone | 1.89115726 | 0.002935485 | 1.786452138 | Up |
| 1-Allyl 2-pentadecyl 1,2-pyrrolidinedicarboxylate | 1.55742588 | 0.038067981 | 1.759259664 | Up |
| 3alpha,12beta-Dihydroxy-5alpha-cholan-24-oic Acid | 1.55742588 | 0.038067981 | 1.759259664 | Up |
| 4-bromo-1-methyl-1H-pyrazole-3-carbaldehyde N-(3,5-dichlorophenyl)hydrazone | 1.034951875 | 0.030080555 | 1.742145959 | Up |
| Dimethyl-5-sulfoisophthalate | 1.486396764 | 0.033086169 | 1.722841224 | Up |
| Santonin | 1.302067133 | 0.044479611 | 1.708835727 | Up |
| Tetrahydrocortisone | 2.046093789 | 0.000306764 | 1.704746511 | Up |
| Sulfolithocholylglycine | 1.596660345 | 0.008720777 | 1.697268961 | Up |
| 2,6-Di-tert-butylphenol | 1.944220677 | 0.00019428 | 1.69166358 | Up |
| LPA 9:0 | 1.638219784 | 0.00811489 | 1.687548473 | Up |
| N-({(2R,3S,4R,5S)-3,4-Dihydroxy-5-[2-(isopropylamino)-2-oxoethyl]tetrahydro-2-furanyl}methyl)-4-methoxybenzamide | 1.67645733 | 0.011747127 | 1.67944955 | Up |
| Sebuthylazine-Desethyl | 1.323644608 | 0.035535766 | 1.679194229 | Up |
| Bacillamidin D | 1.550793201 | 0.030599928 | 1.67514614 | Up |
| (2beta,3alpha,5alpha)-2-(2,2-Dimethyl-4-morpholinyl)-3-hydroxy-20-oxopregnan-21-yl acetate | 1.481391679 | 0.039903205 | 1.669465706 | Up |
| NP-006862 | 1.372481707 | 0.021506786 | 1.662784162 | Up |
| (R)-Equol | 1.351463614 | 0.008888008 | 1.660281052 | Up |
| Vorapaxar | 1.848823768 | 0.020949612 | 1.637332974 | Up |
| AIDA | 1.236115089 | 0.034896115 | 1.627282975 | Up |
| N′-hydroxy-2-{[3-(trifluoromethyl)pyridin-2-yl]thio}ethanimidamide | 1.974829744 | 1.34958E-06 | 1.607369898 | Up |
| p-Cresol sulfate | 1.418919499 | 0.022731173 | 1.600874045 | Up |
| N′-[(4-Bromophenyl)sulfonyl]-1,2,3-thiadiazole-4-carbohydrazide | 1.127270749 | 0.03837939 | 1.599663659 | Up |
| Patulin | 1.738274942 | 0.008132332 | 1.581650711 | Up |
| Indolepyruvate | 1.125255725 | 0.029349511 | 1.576136837 | Up |
| Mesoridazine | 1.706898914 | 0.001718909 | 1.564679024 | Up |
| 3-(tetradecyloxy)propylamine | 1.692944838 | 0.013327625 | 1.540422067 | Up |
| Fenamiphos-deisopropyl | 1.656556257 | 0.00535455 | 1.539786033 | Up |
| {[5-(2,4-Dichlorophenyl)-4-phenyl-4H-1,2,4-triazol-3-yl]sulfanyl}acetic acid | 1.954294928 | 8.07388E-05 | 1.53827853 | Up |
| N-[2-Amino-5-(butylcarbamoyl)-3-hydroxy-6-methylheptyl]-4-ethyl-N-isopropyl-3-(3-methoxypropoxy)benzamide | 1.469097845 | 0.008871772 | 1.53138698 | Up |
| (R)-5-((R)-2-amino-2-carboxyethyl)-4,5-dihydroisoxazole-3-carboxylic acid | 1.899127245 | 0.00163493 | 1.519648551 | Up |
| Alepric acid | 1.988823324 | 0.000363948 | 1.514854857 | Up |
| 5,7-Dihydroxyflavone | 1.482463522 | 0.028439915 | 1.511398458 | Up |
| methyl (1-{[(3S,4S)-1-(cyclohexylcarbonyl)-4-phenylpyrrolidin-3-yl]methyl}piperidin-4-yl)(cyclopentylmethyl)carbamate | 1.187644588 | 0.01859209 | 1.502607982 | Up |
| 1-(3-Cyanophenyl)-3-{[(2R,4S,5S)-5-{[4-(4-fluorophenyl)-1-piperazinyl]methyl}-1-azabicyclo[2.2.2]oct-2-yl]methyl}urea | 1.623442906 | 0.016428898 | 1.487946726 | Up |
| 1-Fluorocyclopropanecarboxylic acid | 1.600741025 | 0.010981304 | 1.477446408 | Up |
| 1-Phenylhexahydropyridazine-3,6-dione | 1.898597028 | 3.47031E-05 | 1.477225972 | Up |
| L-Dopa | 1.560766289 | 0.011762964 | 1.470552882 | Up |
| 4-Nitrobenzylphosphonic acid | 2.005333208 | 0.000547507 | 1.469786704 | Up |
| L-Glutathione (reduced) | 1.521490029 | 0.020296382 | 1.46664296 | Up |
| 2-{[5-(2-Furyl)-4-isopropyl-4H-1,2,4-triazol-3-yl]sulfanyl}-N-(5-methyl-1,3,4-thiadiazol-2-yl)propanamide | 1.492966192 | 0.049673422 | 1.465111802 | Up |
| NP-001134 | 1.631086336 | 0.007526066 | 1.462455444 | Up |
| 6-methyl-7-nitro-2,3-dihydro-1,4-benzodioxine | 1.617414999 | 0.011119687 | 1.450803213 | Up |
| Ambrosic acid | 1.691514518 | 0.005383707 | 1.426922795 | Up |
| (3aR,7aS,8S,9aR)-5,8-dimethyl-3-methylidene-2H,3H,3aH,4H,6H,7H,7aH,8H,9H,9aH-azuleno[6,5-b]furan-2,6-dione | 1.277469163 | 0.027959811 | 1.424551388 | Up |
| O-methoxycatechol-O-sulphate | 1.581518001 | 0.016407134 | 1.41676489 | Up |
| (2R,3R,4R)-2-Amino-4-hydroxy-3-methylpentanoic acid | 1.768430195 | 0.003561551 | 1.413834549 | Up |
| N'-(4-chlorophenyl)-N-methyl-N-(2-methyl-4,5,6,7-tetrahydro-2H-indazol-3-yl)urea | 1.635841529 | 0.009025512 | 1.413252984 | Up |
| NCGC00384756-01_C27H42O4_(3beta,5alpha,8xi,9xi,14xi,25S)-3-Hydroxyspirostan-12-one | 1.685920645 | 0.018853026 | 1.412360326 | Up |
| 3-Chloro-4,5-dihydroxybenzoic acid | 1.584280431 | 0.016706964 | 1.387718946 | Up |
| gamma-Glutamylalanine | 1.58762958 | 0.016995527 | 1.386076024 | Up |
| Coniferaldehyde | 1.644735692 | 0.01999119 | 1.382221882 | Up |
| Acetylpinnasterol | 1.695836576 | 0.00300539 | 1.380051722 | Up |
| N,N′-(Disulfanediyldi-2,1-ethanediyl)bis(2-iodoacetamide) | 1.063782311 | 0.047711837 | 1.366943819 | Up |
| bromocyclohexylmethane | 2.029874721 | 1.98342E-05 | 1.362607631 | Up |
| (2S)-2,4-Diammoniobutanoate | 1.561375748 | 0.039963245 | 1.360917404 | Up |
| Anilic Acid | 1.474869539 | 0.015920392 | 1.359175877 | Up |
| Isoflavanone base + 3O, O-Hex | 1.55454916 | 0.020770087 | 1.357289738 | Up |
| 2-Ethyl-2-oxazoline | 1.651688429 | 0.013329771 | 1.346749559 | Up |
| NP-020713 | 1.799154877 | 0.007771723 | 1.345278481 | Up |
| 2-(acetylamino)-3-[4-(acetylamino)phenyl]acrylic acid | 1.581542066 | 0.022120038 | 1.344612257 | Up |
| 2-Aminoacetophenone | 1.46915233 | 0.022285705 | 1.341080451 | Up |
| Isoleucyl-Glutamate | 1.737338498 | 0.001132119 | 1.335069142 | Up |
| Nebidrazine | 1.356154067 | 0.015268379 | 1.333330588 | Up |
| 1-[2-{3-[4-(4-Butylphenyl)-1H-imidazol-1-yl]propoxy}-6-(dimethylamino)phenyl]-3-pentylurea | 1.705645257 | 0.004512823 | 1.328350675 | Up |
| Type IV cyanolipid 20:2 ester | 1.743854039 | 0.006580498 | 1.320662845 | Up |
| 3-Vinylaniline | 1.980915327 | 0.000420349 | 1.310198275 | Up |
| NP-020400 | 1.540744459 | 0.034733329 | 1.300593588 | Up |
| N-[(1S,3aS,5S,7aR)-5-Hydroxy-7a-{3-[4-(2-methoxyphenyl)-1-piperazinyl]-3-oxopropyl}-3,3,5-trimethyloctahydro-1H-inden-1-yl]nicotinamide | 1.661267605 | 0.007844571 | 1.29630683 | Up |
| N-{4-[2,4-Bis(2-methyl-2-butanyl)phenoxy]butyl}-1-(2,4-dimethylphenyl)-3-(2-fluorophenyl)-1H-pyrazole-5-carboxamide | 1.453643883 | 0.016636167 | 1.293970606 | Up |
| N1-(4-fluorophenyl)-2-[(4-methylphenyl)thio]acetamide | 1.519953971 | 0.019679897 | 1.284213863 | Up |
| 3,7-Epoxycaryophyllan-6-Ol | 1.888549841 | 0.002734277 | 1.267193842 | Up |
| Methyl 4-{[1,3-dihydroxy-1-(4-hydroxy-3-methoxyphenyl)-2-propanyl]oxy}-3-methoxybenzoate | 1.75617428 | 0.001956945 | 1.255400806 | Up |
| Ethyl 4-{[3-(dimethylamino)propyl]amino}-1-methyl-1H-pyrazolo[3,4-b]pyridine-5-carboxylate | 1.435386647 | 0.035376647 | 1.254749206 | Up |
| Sarsasapogenin | 1.368819857 | 0.04613134 | 1.242537617 | Up |
| 5-(5-methoxycarbonyl-5,8a-dimethyl-2-methylidene-3,4,4a,6,7,8-hexahydro-1H-naphthalen-1-yl)-3-methylpentanoic acid | 1.47547468 | 0.025957835 | 1.238877273 | Up |
| gamma-Glutamylleucine | 1.942263218 | 0.000219698 | 1.236230314 | Up |
| Boc-Pyr-OMe | 1.942263218 | 0.000219698 | 1.236230314 | Up |
| 3-Hydroxy-2,6-bis(hydroxymethyl)-4H-pyran-4-one | 1.280506263 | 0.028995485 | 1.23254654 | Up |
| 3-Dehydrocarnitine | 1.688229332 | 0.011202316 | 1.231455356 | Up |
| Lumiracoxib | 1.993525988 | 8.16541E-06 | 1.218609199 | Up |
| 4-Hydroxy-3-(2'-hydroxy-[1,1'-biphenyl]-4-yl)-6-oxo-6,7-dihydrothieno[2,3-b]pyridine-5-carbonitrile | 1.966818172 | 0.000254684 | 1.215999044 | Up |
| Methyl 3,3,3-trifluoropyruvate | 1.417598812 | 0.046897666 | 1.196859017 | Up |
| L-γ-Glutamyl-3-(sulfosulfanyl)-L-alanylglycine | 1.351106447 | 0.035571899 | 1.187914687 | Up |
| Salvinorin B | 1.774566524 | 0.008436437 | 1.184467061 | Up |
| 7-Epi-12-hydroxyjasmonic acid glucoside | 1.783033964 | 0.007073753 | 1.172965422 | Up |
| Phenyl trifluoromethylsulfone | 1.416561497 | 0.008556248 | 1.169787574 | Up |
| 4-[4-(methoxymethyl)-6-piperidinopyrimidin-2-yl]-2-methyl-1,3-thiazole | 1.505461105 | 0.022486633 | 1.166743713 | Up |
| Tetrachloro-m-xylene | 1.365528795 | 0.042432124 | 1.16146486 | Up |
| L-Theanine | 2.011797636 | 0.00012955 | 1.157321683 | Up |
| Acetamiprid | 1.474849625 | 0.009208109 | 1.156884381 | Up |
| 3,6,9,12,15-octadecapentaenoic acid | 1.602566522 | 0.023141562 | 1.154569004 | Up |
| Pinnasterol | 1.828904835 | 0.000116347 | 1.145281454 | Up |
| N2-Acetylornithine | 1.905589044 | 0.00056625 | 1.142559337 | Up |
| (R)-2,3-Dihydroxy-3-methylvalerate | 1.276063015 | 0.027814096 | 1.140984763 | Up |
| Methionine sulfoxide | 1.683432991 | 0.011116866 | 1.134888768 | Up |
| Hippuric acid | 1.380868329 | 0.029466499 | 1.13220882 | Up |
| Cortisone | 1.870372008 | 0.000712891 | 1.124983307 | Up |
| 2,7-Bis(methylthio)-1,3,4-thiadiazolo[3,2-a](1,3,5)triazine-5-one | 1.316381962 | 0.023936088 | 1.119397082 | Up |
| Triethylene glycol dimethacrylate | 1.373157363 | 0.014389984 | 1.118715055 | Up |
| tolufazepam | 1.748862157 | 0.001102218 | 1.104689611 | Up |
| 4-Methyl-N-[6-(methylsulfonyl)-1,3-benzothiazol-2-yl]benzenesulfonamide | 1.193880717 | 0.030577503 | 1.104659345 | Up |
| (2R,4S,5S,7S)-5-Amino-N-(3-cyanopropyl)-4-hydroxy-7-[4-methoxy-3-(3-methoxypropoxy)benzyl]-2,8-dimethylnonanamide | 1.74522101 | 0.00636798 | 1.103036326 | Up |
| Aspartame | 1.863549742 | 0.000935198 | 1.097067583 | Up |
| N-[(7-Hydroxy-4-methyl-2-oxo-2H-chromen-8-yl)methyl]-N-methylglycine | 1.863549742 | 0.000935198 | 1.097067583 | Up |
| 6-(tert-Butylsulfonyl)-2-methylpyrazolo[1,5-a]pyrimidin-7-amine | 1.811640614 | 0.001375575 | 1.094700908 | Up |
| N,N-Dimethyltetradecylamine-N-oxide | 1.86055992 | 0.002061194 | 1.094046338 | Up |
| IDP | 1.181659872 | 0.031392469 | 1.091830223 | Up |
| Thianthrene oxide | 1.430799357 | 0.040148892 | 1.086855561 | Up |
| Acetylcholine | 1.758930872 | 0.002444193 | 1.075167088 | Up |
| 7-(3-Bromophenyl)-2-[(2-chlorobenzyl)sulfanyl]-5-methyl-1,7-dihydro[1,2,4]triazolo[1,5-a]pyrimidine-6-carboxamide | 1.687823763 | 0.000497801 | 1.068854615 | Up |
| 4-(Trifluoromethyl)benzylsulfonyl chloride | 1.432700557 | 0.048055838 | 1.065220677 | Up |
| Benzothiazol-2-ylacetonitrile | 1.685290339 | 0.002963162 | 1.06469566 | Up |
| 1-(3,5-Dihydroxyphenyl)-12-hydroxy-2-tridecanyl acetate | 1.20373963 | 0.049413454 | 1.052402454 | Up |
| chlorovulone I | 1.449862508 | 0.047713613 | 1.042776159 | Up |
| 2-Cyano-3-phenylquinoxaline | 1.43816237 | 0.025239653 | 1.040745188 | Up |
| MG(0:0/PGE2/0:0) | 1.755459866 | 0.000824038 | 1.026921233 | Up |
| 2,5-Furandicarboxylic acid | 1.561319571 | 0.013222966 | 1.02152069 | Up |
| Midazolam | 1.563452089 | 0.007377842 | 1.02136926 | Up |
| N-Benzyl-N-hexyl-N~2~,N~6~-bis{[(2-methyl-2-propanyl)oxy]carbonyl}lysinamide | 1.782928139 | 0.001929034 | 1.018906758 | Up |
| N-Benzoyl-4-O-methoxyanthranilate | 1.913281974 | 0.000230756 | 1.017716362 | Up |
| Nor-9-carboxy-δ9-THC | 1.754902816 | 0.018727664 | 1.00873342 | Up |
| 5-[5,6-Dichloro-1-(2,4-dichlorobenzyl)-1H-benzimidazol-2-yl]pyridin-2(1H)-one | 1.379456789 | 0.013679145 | 1.007083434 | Up |
| Lotaustralin | 1.465008226 | 0.023722739 | 1.000336564 | Up |
| FA 16:3 | 1.92917171 | 0.000426345 | -1.001054383 | Down |
| Melafolone | 1.643493663 | 0.005029982 | -1.001879755 | Down |
| Lysyltryptophyllysine | 1.098959836 | 0.045687901 | -1.004638157 | Down |
| Fenfluramine | 1.250196988 | 0.025777708 | -1.00862629 | Down |
| 7-(2-hydroxypropan-2-yl)-1,4a-dimethyl-2,3,4,5,6,7,8,8a-octahydronaphthalen-1-ol | 1.626090611 | 0.039369475 | -1.013445376 | Down |
| 6-Chloroapigenin | 1.715637365 | 0.002031956 | -1.022311578 | Down |
| 3-Hydroxymethylglutaric acid | 1.538024805 | 0.008832665 | -1.025528903 | Down |
| Didanosine | 1.867515704 | 0.000129141 | -1.026726071 | Down |
| 3-hydroxy-C10-homoserine lactone | 1.701468553 | 0.000871242 | -1.027919987 | Down |
| 1,2-Cyclohexanediol, 1-methyl-4-(1-methylethenyl)- | 1.46068045 | 0.020904806 | -1.030883256 | Down |
| Penicillic acid | 1.69470436 | 0.007143136 | -1.034565342 | Down |
| NP-006122 | 1.4813161 | 0.016968762 | -1.041660829 | Down |
| X-GlcNAc | 1.371057117 | 0.033746458 | -1.048352928 | Down |
| NP-021701 | 1.590398054 | 0.008640176 | -1.049685243 | Down |
| 2-Aminooctanedioic acid | 1.622778427 | 0.011555409 | -1.05411521 | Down |
| Benzocaine | 1.108894167 | 0.044035378 | -1.060290316 | Down |
| 2-hydroxy pelargonic acid | 1.440605646 | 0.017763642 | -1.066724446 | Down |
| Pestalotin | 1.210814017 | 0.025234988 | -1.06747103 | Down |
| NCGC00180037-02!2-hydroxy-4-methoxy-3-(3-methylbut-2-enyl)-6-pentylbenzoic acid | 1.430203683 | 0.014906913 | -1.068700784 | Down |
| Acetylglycine | 2.116394366 | 0.000121422 | -1.07200285 | Down |
| Pinacidil | 1.522428445 | 0.00861476 | -1.083319027 | Down |
| Sulfanilic acid | 1.660881168 | 0.005141918 | -1.085469159 | Down |
| Erucic amide | 1.486150755 | 0.034753774 | -1.087938322 | Down |
| Diphenyl sulfide | 1.315867302 | 0.041054129 | -1.088360289 | Down |
| N-(4-Methoxybenzyl)-3-{[5-(2-methyl-2-propanyl)-1,2-oxazol-3-yl]methyl}-3-oxetanamine | 1.406311185 | 0.037028731 | -1.092815912 | Down |
| 1-Phenyl-1H-pyrazolo[3,4-d]pyrimidin-4-amine | 1.300296493 | 0.029626162 | -1.095818331 | Down |
| Xanthosine | 1.598625579 | 0.013426538 | -1.09810444 | Down |
| Ethylamine, N,N-dioctyl-2-phenylthio | 1.780375949 | 0.001119216 | -1.100834439 | Down |
| Gabapentin | 1.344397134 | 0.02092862 | -1.103176801 | Down |
| 11-Hydroxy-9-tridecenoic acid | 1.379766266 | 0.033690168 | -1.104295561 | Down |
| 2,3,5-Trichloro-4,6-dicyanophenolate | 1.987862743 | 0.000116588 | -1.108845534 | Down |
| Porfiromycin | 1.321258086 | 0.049078692 | -1.113185981 | Down |
| 6-Chloro-5-fluoro-1H-1,2,3-benzotriazole | 1.655007894 | 0.0013814 | -1.116585601 | Down |
| 13(S)-HOTrE | 1.801883838 | 0.002579881 | -1.120073074 | Down |
| acetamide, 2-[(5-mercapto-1,3,4-thiadiazol-2-yl)thio]-N,N-dimethyl- | 1.366007891 | 0.002080351 | -1.123729909 | Down |
| 1,3-dimethyl-6-(trifluoromethyl)-1H-pyrazolo[3,4-b]pyridin-4-ol | 2.043704204 | 0.000173927 | -1.137186529 | Down |
| N2-Acetyl-L-aminoadipate | 1.724101955 | 0.005984246 | -1.138377431 | Down |
| 3-tert-Butyladipic acid | 1.503635291 | 0.017481477 | -1.143841242 | Down |
| Hemin | 1.400429692 | 0.029638316 | -1.149714117 | Down |
| N-(9-oxodecyl)acetamide | 1.506541862 | 0.021087218 | -1.151081249 | Down |
| 5-Chloro-1-methyl-3-[[(4-methylphenyl)thio]methyl]-1H-pyrazole-4-carboxaldehyde | 1.714878533 | 0.006921332 | -1.154840662 | Down |
| [{4-[(1E)-3-Amino-2-cyano-3-oxo-1-propen-1-yl]phenyl}(hydroxy)methyl]phosphonic acid | 1.898861811 | 0.000164894 | -1.161174022 | Down |
| N-[(5-Chloro-1,2,3-thiadiazol-4-yl)methyl]-4-(trifluoromethyl)aniline | 1.894767195 | 0.000448681 | -1.172995247 | Down |
| Dihydrocapsaicin | 1.62068634 | 0.0074319 | -1.176763069 | Down |
| 6,8-Dibromo-3-(1H-tetrazol-5-yl)-2H-chromen-2-one | 1.537790506 | 0.016553423 | -1.18159018 | Down |
| [2,3-Bis(3,4-dimethoxyphenyl)-6-quinoxalinyl][4-(4-methoxyphenyl)-1-piperazinyl]methanone | 1.277692383 | 0.019864805 | -1.188053821 | Down |
| PC 18:2 | 1.252609605 | 0.007590744 | -1.188569229 | Down |
| 6-(Trifluoromethyl)-1H-benzotriazol-1-ol | 1.750477724 | 0.002780102 | -1.195471072 | Down |
| 4-methylpyridine-3-sulfonic acid | 1.753034146 | 0.000769045 | -1.200888433 | Down |
| 2-Aminomuconic acid | 1.572512799 | 0.010969176 | -1.20248179 | Down |
| DIETHYL (BOC-AMINO)MALONATE | 1.287659933 | 0.047942033 | -1.218830591 | Down |
| NP-016437 | 1.786527409 | 0.006623452 | -1.219193598 | Down |
| Aza-18-crown-6 | 1.552822023 | 0.034561723 | -1.224844632 | Down |
| Temozolomide | 1.638211112 | 0.005866524 | -1.234903292 | Down |
| 4-(Prop-2-ynyl)thiomorpholine 1,1-dioxide | 1.719461718 | 0.00124209 | -1.236401988 | Down |
| clodanolene | 1.863081292 | 0.004507021 | -1.237375207 | Down |
| Stearidonic acid | 1.734192705 | 0.000739256 | -1.23780929 | Down |
| 3-heptynoic acid | 1.796118302 | 0.003042771 | -1.243645177 | Down |
| 8-Amino-7-oxononanoic acid | 1.707768624 | 0.004547617 | -1.243844852 | Down |
| Ureidoisobutyric acid | 1.320562904 | 0.027722699 | -1.245022123 | Down |
| Boscalid | 1.592677522 | 0.012217775 | -1.246809943 | Down |
| 3-oxo-C12 homoserine lactone | 1.935712294 | 3.3762E-05 | -1.252225626 | Down |
| [1,4-Bis(3,4,5-trimethoxybenzoyl)-2-piperazinyl]methyl (3-methylbutyl)carbamate | 1.601500005 | 0.035776203 | -1.255091871 | Down |
| 6-(2,5-Dioxo-2,5-dihydro-1H-pyrrol-1-yl)hexanoic acid | 1.345981636 | 0.019734343 | -1.258076905 | Down |
| N-Acetyl-L-tyrosine | 1.403756699 | 0.026417848 | -1.258831429 | Down |
| 4-Amino-1-ethyl-N-[(1-methyl-1H-pyrazol-4-yl)methyl]-1H-pyrazole-3-carboxamide | 1.382950929 | 0.048065261 | -1.271781389 | Down |
| Cyphenothrin | 1.605821295 | 0.022961172 | -1.272249452 | Down |
| 2,2′-disulfanediylbis(N-{4-[(6-chloropyridazin-3-yl)sulfamoyl]phenyl}benzamide) | 1.934219766 | 0.000289685 | -1.30332716 | Down |
| 4-Acetamidobutanoic acid | 1.984391688 | 0.000373188 | -1.305101563 | Down |
| (R)-3-((R)-3-Hydroxybutanoyloxy)butanoate | 1.567466506 | 0.03226777 | -1.30669079 | Down |
| 2-(4-bromoanilino)-1-phenylethanone | 2.104569118 | 1.79034E-05 | -1.308467953 | Down |
| (8aR,12S,12aR)-12-hydroxy-4-methyl-4,5,6,7,8,8a,12,12a-octahydro-1H-3-benzoxecine-2,9-dione | 1.87889147 | 7.95967E-05 | -1.315196016 | Down |
| N-[(3s)-2-Oxotetrahydrofuran-3-Yl]butanamide | 1.791755412 | 0.003839885 | -1.320008904 | Down |
| Isobutyrylglycine | 1.889594586 | 0.001477089 | -1.324399039 | Down |
| 4'-Methoxy-alpha-ethylaminovalerophenone | 1.688376169 | 0.00215391 | -1.338064019 | Down |
| Quassin | 1.428754878 | 0.026011046 | -1.340509952 | Down |
| 1-Methyl-2-nitro-3-((tetrahydrofuran-3-yl)methyl)guanidine | 1.603638426 | 0.030264783 | -1.346687188 | Down |
| 4,4'-Thiobisbenzenethiol | 1.669188277 | 0.010522336 | -1.359517137 | Down |
| 1,3-Dimethylimidazolidin-2-imine | 1.509582701 | 0.036003079 | -1.36078853 | Down |
| N-Acetyl-L-methionine | 1.881521214 | 0.000753122 | -1.371020875 | Down |
| 4-Chloro-5-(4-fluorophenyl)thieno[2,3-d]pyrimidine | 1.543004946 | 0.008021111 | -1.372262659 | Down |
| 1,2,4-triazin-5-ol, 3-mercapto-6-phenyl- | 1.147459544 | 0.008055431 | -1.374982062 | Down |
| Mesotrione | 2.116432842 | 1.42475E-05 | -1.377011013 | Down |
| 5-Methyl-3-{[2-(4-nitrophenyl)-2-oxoethyl]sulfanyl}-1,2-thiazole-4-carbonitrile | 1.674693814 | 0.006600616 | -1.379906615 | Down |
| N-(dodecanoyl)-homoserine lactone | 1.877010497 | 3.15657E-05 | -1.381313699 | Down |
| But-2-enoic acid | 1.721754908 | 0.001147985 | -1.391949177 | Down |
| 4-(5-Cyano-1H-1,2,3-triazol-4-yl)phenyl 3,5-dimethylisoxazole-4-carboxylate | 1.811778433 | 0.002727218 | -1.392267368 | Down |
| NP-006255 | 1.692048489 | 0.002074713 | -1.394357293 | Down |
| Haloxyfop-P | 2.150686661 | 1.03625E-05 | -1.407166182 | Down |
| Phe-leu-arg-phe | 1.770493322 | 0.002107377 | -1.409011782 | Down |
| (2R,4S,5S)-5-{[(2S)-2-{(3R)-3-Benzyl-4-[(4-methyl-1-piperazinyl)carbonyl]-2-oxo-1-piperazinyl}hexanoyl]amino}-N-butyl-6-cyclohexyl-4-hydroxy-2-isopropylhexanamide | 1.65201557 | 0.011759706 | -1.417113278 | Down |
| methyl 3-(methylamino)-2-[(4-methyl-1,2,3-thiadiazol-5-yl)carbonyl]but-2-enoate | 1.223840525 | 0.023256301 | -1.425302049 | Down |
| N-(Cyclopentylmethyl)-N-{(2S)-2-hydroxy-3-[{[(1S,2R)-2-hydroxy-2,3-dihydro-1H-inden-1-yl]carbamoyl}(2-phenylethyl)amino]propyl}-4-methoxybenzenesulfonamide | 1.736638007 | 0.004976717 | -1.426852489 | Down |
| 1-[Acetyl(octyl)amino]-1-deoxypentitol | 1.865923821 | 0.001056163 | -1.427646771 | Down |
| N-Methyl-5-{5-[(2S)-1-(4-nitrobenzyl)-2-pyrrolidinyl]-1,2,4-oxadiazol-3-yl}-2-pyridinamine | 1.533519671 | 0.021187574 | -1.43368342 | Down |
| Epigoitrin | 1.913502953 | 0.000284863 | -1.444028218 | Down |
| N-(3-aminopropyl)-N,N′-bis(3-phenylpropyl)-N′-{3-[(3-phenylpropyl)amino]propyl}butane-1,4-diamine | 1.789751187 | 0.001602 | -1.444090905 | Down |
| 3,4-Dihydro-6-hydroxy-2,5,7,8-tetramethyl-2H-1-benzopyran-2-carboxylic acid | 1.843184856 | 0.007326286 | -1.446014766 | Down |
| NP-000124 | 1.766099409 | 0.016912401 | -1.446154124 | Down |
| 4-(2-Chloro-6-fluorophenyl)-1,3-thiazol-2-amine | 1.878462557 | 0.023912049 | -1.454803655 | Down |
| Hexanoylglycine | 2.1605419 | 6.01161E-05 | -1.475964963 | Down |
| 4-Trimethylammoniobutanal | 2.148159048 | 2.73412E-05 | -1.47622317 | Down |
| {1-[(3,3-Dimethylbutyl)sulfonyl]-4-piperidinyl}(4-methoxyphenyl)methanone | 1.429871493 | 0.042443368 | -1.477912651 | Down |
| (plus_minus)9-HpODE | 1.659458377 | 0.045411257 | -1.498094531 | Down |
| 1,1′-(1,10-Dioxo-1,10-decanediyl)bis[3-(2-methoxyphenyl)-2-thioxodihydro-4,6(1H,5H)-pyrimidinedione] | 1.413221475 | 0.012463015 | -1.502064237 | Down |
| 2-[5-[2-[2-[5-(2-hydroxypropyl)oxolan-2-yl]propanoyloxy]propyl]oxolan-2-yl]propanoic acid | 1.835845648 | 0.001218444 | -1.503613327 | Down |
| 2C-D | 1.337401483 | 0.039264544 | -1.523937029 | Down |
| 4-(Mesitylsulfonyl)-N-methyl-N-(4-methylphenyl)-1-piperazinecarboxamide | 1.301994151 | 0.034530692 | -1.529870097 | Down |
| NP-012972 | 1.695918246 | 0.00214084 | -1.534181403 | Down |
| Adipic acid | 1.395734979 | 0.036983165 | -1.536766998 | Down |
| 2-(3-chlorophenyl)-5-[4-(trifluoromethyl)-3-pyridyl]-1,3,4-oxadiazole | 1.732869637 | 0.009995176 | -1.544185178 | Down |
| Folcisteine | 1.669733841 | 0.01342219 | -1.55056883 | Down |
| Trimethadione | 1.775694058 | 0.00265992 | -1.556910606 | Down |
| p-Chlorobenzoic anhydride | 2.087823261 | 8.01102E-05 | -1.579142243 | Down |
| 10-Hydroxydecanoic acid | 1.798439304 | 0.006323299 | -1.586857058 | Down |
| Isopropyl {3-chloro-5-[1-({6-[2-(5-ethyl-4-methyl-1,3-thiazol-2-yl)ethyl]-4-(4-morpholinyl)-2-pyridinyl}amino)ethyl]phenyl}carbamate | 1.470760576 | 0.040719265 | -1.589160926 | Down |
| [Similar to: Dinotefuran | 1.485277308 | 0.02730639 | -1.598906508 | Down |
| O-(1-Carboxyvinyl) phosphorothioate | 1.317968082 | 0.011373058 | -1.603591663 | Down |
| NP-012888 | 1.48301786 | 0.016920821 | -1.618770307 | Down |
| 1-(1,3-Benzothiazol-2-yl)-2,2,2-trifluoro-1,1-ethanediol | 1.038518538 | 0.005548798 | -1.633270678 | Down |
| Methyl (5E,13E)-16,16-dimethyl-9,11,15-tris[(trimethylsilyl)oxy]prosta-5,13-dien-1-oate | 1.895115304 | 0.001719387 | -1.642041559 | Down |
| Sulforidazine | 1.787937291 | 0.004531487 | -1.64685062 | Down |
| Cladosporester C | 1.689782923 | 0.011143096 | -1.65344226 | Down |
| (4E)-5-(4-Chlorophenyl)-2-(1,3-dithietan-2-ylidene)-3-oxo-4-pentenamide | 2.175991651 | 2.90874E-07 | -1.663050707 | Down |
| 2-oxosuberate | 1.650218007 | 0.011653081 | -1.67053346 | Down |
| Tedatioxetine | 1.599669398 | 0.017870855 | -1.689940264 | Down |
| 1-Hexadecyl-3-propylurea | 1.514891013 | 0.043967053 | -1.707939994 | Down |
| 4-methoxynaphthalen-1-yl-(1-pentyl-2-methylindol-3-yl)methanone | 1.873442115 | 0.006168117 | -1.715084503 | Down |
| (2S)-2-{[1-(R)-Carboxyethyl]amino}pentanoate | 2.043786028 | 1.70111E-05 | -1.726684737 | Down |
| 2-(2-(4-(2-Methoxyphenyl)piperazin-1-yl)ethyl)isoindoline-1,3-dione | 1.859719362 | 0.002765503 | -1.74066856 | Down |
| 1,4a-Dimethyl-6-methylene-5-[2-(2-oxo-2,5-dihydro-3-furanyl)ethyl]decahydro-1-naphthalenecarboxylic acid | 1.479940954 | 0.023852672 | -1.741825879 | Down |
| Farnesyltriphosphate | 1.825636484 | 0.002635334 | -1.743396667 | Down |
| 7-(4-Chlorophenyl)-2-(2,6-difluorophenyl)[1,2,4]triazolo[1,5-a]pyridine-8-carbonitrile | 1.294815013 | 0.044045614 | -1.757543158 | Down |
| Cypridina luciferin | 1.823219349 | 0.005075391 | -1.764410797 | Down |
| Dalcetrapib | 1.681338112 | 0.017105968 | -1.765256621 | Down |
| Enviradene | 1.428570506 | 0.019977005 | -1.770450473 | Down |
| dimethyl 2-phenyl-6-(2-thienylcarbonyl)-2,3-dihydro-3,4-pyridazinedicarboxylate | 1.768551341 | 0.011679282 | -1.770818343 | Down |
| 3,6-Dihydroxy-2,7-naphthalenedisulfonic acid | 2.006318084 | 0.000536324 | -1.77770477 | Down |
| Dialuric acid | 1.493098646 | 0.034342091 | -1.784544923 | Down |
| N1-phenethylbenzene-1-carbothioamide | 2.224748568 | 7.94683E-06 | -1.784585388 | Down |
| Ethyl 4-(4-biphenylyl)-2,7,7-trimethyl-5-oxo-1,4,5,6,7,8-hexahydro-3-quinolinecarboxylate | 1.932747171 | 0.00127458 | -1.795241979 | Down |
| Anandamide (20:1, n-9) | 1.53377226 | 0.01811324 | -1.805899942 | Down |
| Capryloylglycine | 1.742623484 | 0.00748472 | -1.821461781 | Down |
| 4-(4-hydroxy-2,2,6-trimethyl-6-{[(2S,3R,4S,5S,6R)-3,4,5-trihydroxy-6-(hydroxymethyl)oxan-2-yl]oxy}cyclohexylidene)but-3-en-2-one | 1.773262087 | 0.010162777 | -1.823631034 | Down |
| Maltol | 1.695551347 | 0.002352882 | -1.838019524 | Down |
| NP-019483 | 1.826911697 | 0.004662027 | -1.84600359 | Down |
| 7-{[(2E)-3,7-Dimethyl-2,6-octadien-1-yl]oxy}-1,3-dihydroxy-9H-xanthen-9-one | 1.626769805 | 0.020480869 | -1.846113123 | Down |
| Diazene, bis(2,4,6-trinitrophenyl)- | 1.933231156 | 0.001325561 | -1.854244401 | Down |
| Ofloxacin ethyl ester | 1.707627881 | 0.004163293 | -1.857669246 | Down |
| Satranidazole | 2.183325129 | 2.64676E-05 | -1.860378836 | Down |
| Trospium | 1.871907545 | 0.00348869 | -1.904137138 | Down |
| 9,10-Bis(chloromethyl)anthracene | 1.949854851 | 0.00478977 | -1.905024495 | Down |
| 6-Bromohexanenitrile | 2.056299599 | 2.08947E-05 | -1.907015798 | Down |
| 5-(2-Ethoxyethyl)-1,3,4-thiadiazol-2-amine | 2.009260984 | 0.000805829 | -1.915509056 | Down |
| N-Dodecylacrylamide | 1.455959715 | 0.043935727 | -1.930489606 | Down |
| 17-Hydroxyandrostane-3-glucuronide | 1.234892567 | 0.045279786 | -1.947498557 | Down |
| (2S,3S,4R,5R,6R)-2-[2,4-Dihydroxy-6-(hydroxymethyl)phenyl]-5-{[(2S,3R,4S,5R,6R)-4,5-dihydroxy-6-methyl-3-{[(2R,3R,4S,5R,6S)-3,4,5,6-tetrahydroxytetrahydro-2H-pyran-2-yl]oxy}tetrahydro-2H-pyran-2-yl]ox y}-3-hydroxy-6-(hydroxymethyl)tetrahydro-2H-pyran-4-yl (2E,4E,7S,8E,10E)-7-hydroxy-2,4,8,10-hexadecatetraenoate | 1.331724081 | 0.047683588 | -1.949139298 | Down |
| 2-(6-Hydroxyhexyl)-3-methylenesuccinic acid | 1.730361355 | 0.006514024 | -1.965268734 | Down |
| 4-Bromo-N-[2-(1-pyrrolidinyl)ethyl]benzenesulfonamide | 1.507392408 | 0.013840708 | -1.980001565 | Down |
| [4-(2,2-Dicyanovinyl)benzyl]phosphonic acid | 1.973588167 | 0.002286269 | -1.989314338 | Down |
| 1-Dodecyl-3-(2,2,3,3-tetramethylbutyl)urea | 1.524110706 | 0.038962784 | -1.998083026 | Down |
| Bucillamine | 1.885683325 | 0.002475041 | -2.013685803 | Down |
| 6-Bromo-3'-methylflavone | 1.900066169 | 3.19666E-05 | -2.019083773 | Down |
| 1-(acetyloxy)-3-hydroxy-6,8a-dimethyl-7-oxo-3-(propan-2-yl)-1,2,3,3a,4,7,8,8a-octahydroazulen-4-yl 4-hydroxybenzoate | 1.650916602 | 0.014608756 | -2.019149087 | Down |
| (5-oxo-2-sulfo-2,5-dihydrofuran-2-yl)acetic acid | 1.711888821 | 0.000159318 | -2.020005871 | Down |
| Sulfametrole | 2.028674431 | 0.000494932 | -2.025611621 | Down |
| N-[2-(Adamantan-1-yloxy)ethyl]-2,4,6-trimethylbenzenesulfonamide | 1.424605292 | 0.033512109 | -2.0349828 | Down |
| 2-Caffeoylisocitrate | 1.841885383 | 0.003453364 | -2.075840608 | Down |
| cis-4-[Methyl(cis-4-{[3-(3,4,5-trimethoxyphenyl)-2-propynoyl]oxy}cyclohexyl)amino]cyclohexyl 9H-fluorene-9-carboxylate | 1.759529412 | 0.01210197 | -2.10345438 | Down |
| 2-{1-[4-(trifluoromethyl)pyrimidin-2-yl]piperidin-4-yl}-2-azaspiro[4.4]nonane-1,3-dione | 1.54818155 | 0.018104844 | -2.122700711 | Down |
| S-{(3E)-4-[(5R,8S,11S)-8-Isopropyl-5-methyl-6,9,13-trioxo-3,17-dithia-14,19,20-triazatricyclo[14.2.1.1~2,5~]icosa-1(18),2(20),16(19)-trien-11-yl]-3-buten-1-yl} octanethioate | 1.67097869 | 0.013890846 | -2.124421465 | Down |
| 3,8,9-trihydroxy-10-propyl-3,4,5,8,9,10-hexahydro-2H-oxecin-2-one | 1.910712399 | 0.007856393 | -2.144413069 | Down |
| MMV676380 | 1.560757273 | 0.014595267 | -2.148904799 | Down |
| 4-Hydroxymytiloxanthin | 1.793243156 | 0.004302491 | -2.154295068 | Down |
| Triamcinolone Benetonide | 2.009431273 | 0.011284999 | -2.161628088 | Down |
| 2,3-Dichloro-N-(1,5-dimethyl-3-oxo-2-phenyl-2,3-dihydro-1H-pyrazol-4-yl)-4-methoxybenzenesulfonamide | 1.320247047 | 0.046110671 | -2.208734511 | Down |
| 3-(3-Chloro-phenyl)-2-mercapto-3H-quinazolin-4-one | 1.908183396 | 0.00054525 | -2.223913193 | Down |
| Dimethyl 3,4-dihydroxy-2,5-thiophenedicarboxylate | 1.628277951 | 0.005943172 | -2.23246737 | Down |
| 3-(2-{3-[4-(trifluoromethyl)phenyl]-1,2,4-oxadiazol-5-yl}ethyl)-2,3-dihydro-1,3-benzoxazol-2-one | 1.004829137 | 0.016689694 | -2.238377632 | Down |
| 5-(4-Acetoxy-1-butynyl)-2,2'-bithiophene | 2.105717005 | 0.000806132 | -2.240498134 | Down |
| ethyl 2-{2-[(phenylsulfonyl)amino]-1,3-thiazol-4-yl}acetate | 1.365965414 | 0.049832985 | -2.243490393 | Down |
| Nigrescin | 1.253542138 | 0.041390131 | -2.251654649 | Down |
| Clofencet-potassium | 1.440564073 | 0.00747379 | -2.254710585 | Down |
| 2-methyl-5-oxocyclopent-1-enyl 2,4-dichloro-5-methylbenzene-1-sulfonate | 1.802297786 | 0.002657113 | -2.257810493 | Down |
| Adenosine phosphosulfate | 1.384666568 | 0.037318537 | -2.261255929 | Down |
| 2,3′,4′-TRICHLOROBENZANILIDE | 2.107633037 | 0.000163892 | -2.298446872 | Down |
| PURPUROGALLIN-4-CARBOXYLIC ACID | 1.248863745 | 0.011719239 | -2.298687589 | Down |
| Methyl 3-{[4-(trifluoromethyl)-2-pyrimidinyl]oxy}-2-thiophenecarboxylate | 1.983324519 | 0.000511526 | -2.299596752 | Down |
| Darunavir | 1.852050856 | 0.006254419 | -2.302669788 | Down |
| 2-Methoxy-5-[(5,6,7-trimethoxy-4-oxo-3,4-dihydro-2H-chromen-3-yl)methyl]phenyl N-{[(2-methyl-2-propanyl)oxy]carbonyl}-L-phenylalaninate | 1.624727794 | 0.017734449 | -2.309378273 | Down |
| lup-20(29)-en-3beta-(1-(2S-hydroxypropionate)-benzoic acid | 1.832472896 | 0.008211106 | -2.320443914 | Down |
| carocainide | 1.768669352 | 0.00632233 | -2.326420798 | Down |
| N-(4-{(1S,3S,7S,10R,11S,12S,16R)-7,11-Dihydroxy-8,8,12,16-tetramethyl-3-[(1E)-1-(2-methyl-1,3-thiazol-4-yl)-1-propen-2-yl]-5,9-dioxo-4,17-dioxabicyclo[14.1.0]heptadec-10-yl}butyl)-3-(2-methoxyethoxy)p ropanamide | 1.442630821 | 0.047986511 | -2.33747371 | Down |
| (2-anilino-4-methyl-1,3-thiazol-5-yl)(1-benzothiophen-2-yl)methanone | 1.961014567 | 0.001878287 | -2.349792469 | Down |
| TRIETHYLENE GLYCOL DITOSYLATE | 1.496095847 | 0.012187677 | -2.375058133 | Down |
| D-γ-Glutamyl-S-[(4S,5R,6E,8E,10Z,13Z,16Z)-1-carboxy-4-hydroxy-6,8,10,13,16-nonadecapentaen-5-yl]-L-cysteinylglycine | 1.878978593 | 0.004255822 | -2.376354286 | Down |
| (2R,3R,4S,5R)-2-[6-Amino-2-(dimethylamino)-9H-purin-9-yl]-5-(2-ethyl-2H-tetrazol-5-yl)tetrahydro-3,4-furandiol | 1.899740611 | 0.003133064 | -2.380033261 | Down |
| (E)-N-(2-Amino-4-fluorophenyl)-3-(1-cinnamyl-1H-pyrazol-4-yl)acrylamide | 1.90687209 | 0.003754161 | -2.389444423 | Down |
| (Iodomethyl)trimethylsilane | 2.117890522 | 0.000147788 | -2.395533349 | Down |
| 5-Sulfosalicylic acid | 1.922767061 | 0.001655345 | -2.396367711 | Down |
| Methyl 3-(2,6-dichlorophenyl)-5-({2-[(methylamino)carbonyl]hydrazino}carbonyl)isoxazole-4-carboxylate | 1.364137124 | 0.042001737 | -2.402205172 | Down |
| 3-[(Carboxymethyl)sulfanyl]-2-oxopropanoic acid | 1.781020227 | 0.000465615 | -2.404596864 | Down |
| ethyl 2-cyano-2-[3-(2,6-dimethylphenyl)-4-phenyl-2,3-dihydro-1,3-thiazol-2-yliden]acetate | 1.634958537 | 0.012146232 | -2.407484749 | Down |
| 1,3-Bis(4-nitrophenyl)urea | 1.328849719 | 0.0425447 | -2.459911922 | Down |
| 5-Sulfoxymethylfurfural | 1.626645876 | 0.003973778 | -2.468758735 | Down |
| 4-[2-(5-bromo-2-thienyl)-4-pyrimidinyl]benzamide | 2.175577283 | 6.67517E-06 | -2.470409381 | Down |
| Fludara | 1.828564901 | 0.00527532 | -2.47490002 | Down |
| 20-Dihydro 6alpha-methylprednisone | 1.84418187 | 0.007285089 | -2.517311434 | Down |
| Ethyl 4-({1-(3,4-dimethoxyphenyl)-2-oxo-2-[(tetrahydro-2-furanylmethyl)amino]ethyl}[N-(2-furoyl)glycyl]amino)benzoate | 1.917820209 | 0.005029812 | -2.532511932 | Down |
| Millettocalyxin A | 1.646524101 | 0.00933922 | -2.540539657 | Down |
| Cilastatin | 1.721983977 | 0.007856788 | -2.55468616 | Down |
| 4-[({[5-(Benzyloxy)-1-(3-carbamimidoylbenzyl)-1H-indol-2-yl]carbonyl}amino)methyl]-N,N,N-trimethylanilinium | 1.838341112 | 0.016257892 | -2.575212371 | Down |
| N′5-(4-chlorobenzoyl)-4-methyl-2-[(2-pyridylsulfonyl)methyl]-1,3-thiazole-5-carbohydrazide | 1.461916989 | 0.022275286 | -2.608237095 | Down |
| 2-Phenyl-N-[2,2,2-trichloro-1-(1-naphthylamino)ethyl]acetamide | 1.924959287 | 0.001386512 | -2.67830417 | Down |
| Carbonyldi-4,1-phenylene bis(2,2-dimethylpropanoate) | 1.705984544 | 0.011454514 | -2.690758773 | Down |
| 1,1′-(1,2-Ethanediyl)bis(1-nitrosourea) | 2.000445148 | 0.009195511 | -2.697780729 | Down |
| 2-[(4-chlorophenyl)sulfonyl]-3-[(2-furylmethyl)amino]-3-(methylthio)acrylonitrile | 1.476188591 | 0.048236795 | -2.711539923 | Down |
| 4-{5-[(3S)-1-(3,4-Difluorobenzyl)-3-pyrrolidinyl]-1,3,4-oxadiazol-2-yl}-N,N-dimethylaniline | 1.690479744 | 0.011565312 | -2.721003321 | Down |
| methyl 2-(6-hydroxy-3-oxo-3H-xanthen-9-yl)benzoate | 1.571993987 | 0.016145777 | -2.741298225 | Down |
| 2-Chloro-1-(5-{(E)-[(2,4-dinitrophenyl)hydrazono]methyl}-1H-pyrrol-3-yl)ethanone | 1.851779594 | 0.001094847 | -2.749827266 | Down |
| PS 36:2 | 1.448605466 | 0.036325699 | -2.757599741 | Down |
| Itraconazole | 1.343452651 | 0.016547571 | -2.774554906 | Down |
| Isouvaretin | 1.538925101 | 0.04192218 | -2.822203828 | Down |
| 1-[2-hydroxy-4-(3-hydroxy-5-methylphenoxy)-6-methylphenyl]-3-methylbutane-2,3-diol | 1.828222683 | 0.009186498 | -2.849132393 | Down |
| (4E)-2-(acetyloxy)-7-hydroxy-6-methoxy-7-(6-oxo-3,6-dihydro-2H-pyran-2-yl)hept-4-en-3-yl acetate | 1.596009953 | 0.024548496 | -2.873710526 | Down |
| 6-bromo-docosa-5E,9Z-dienoic acid | 1.93113191 | 0.018172105 | -2.87802607 | Down |
| 2-Chlorobenzaldehyde 1-[4-(trifluoromethyl)-1,3-thiazol-2-yl]hydrazone | 1.803132706 | 0.00334487 | -2.878398871 | Down |
| 1,4:3,6-Dianhydro-2-deoxy-5-O-(2-methoxy-2-oxoethyl)-2-({[3-(trifluoromethyl)phenyl]carbamoyl}amino)-D-glucitol | 1.66943917 | 0.011322653 | -2.955149001 | Down |
| Dihydroxy(3-nitrophenyl)stibine oxide | 1.700199681 | 0.011254466 | -3.012795337 | Down |
| 2-(1,3-Benzothiazol-2-yl)-3-(4-chlorophenyl)acrylonitrile | 2.066293803 | 0.001025296 | -3.070286228 | Down |
| (2S,3S)-3,5,7-Trihydroxy-6-methyl-2-(3,4,5-trihydroxyphenyl)-2,3-dihydro-4H-chromen-4-one | 1.963703731 | 0.002374407 | -3.082382436 | Down |
| 2,2′,2″,2‴-[(4,8-Diaminopyrimido[5,4-d]pyrimidine-2,6-diyl)dinitrilo]tetraethanol | 2.20174953 | 3.60091E-05 | -3.112114886 | Down |
| Xanthine | 1.595702701 | 0.030893473 | -3.113421688 | Down |
| LysoPI(18:0/0:0) | 1.455490791 | 0.049129373 | -3.208053416 | Down |
| Barbamide | 1.440077537 | 0.048218531 | -3.245911684 | Down |
| 1-(4-chlorophenyl)-3-hydroxy-1,2-dihydroquinoxalin-2-one | 1.817206883 | 0.004793867 | -3.257402559 | Down |
| L-Olivosyl-oleandolide | 2.173413706 | 0.001785028 | -3.265760864 | Down |
| 2- (1,3-Benzodioxol-5-yl) -5-hydroxy-6,8-dimethoxy-4H-1-benzopyran-4-one | 1.415225264 | 0.02165052 | -3.270727547 | Down |
| Crufomate | 1.709118052 | 0.005241353 | -3.306097014 | Down |
| N-{1-[(2-Methoxyphenyl)acetyl]-5,10-dioxododecahydrodipyrrolo[1,2-a:3',2'-E][1,4]diazepin-7-yl}cyclobutanecarboxamide | 1.40810695 | 0.012261799 | -3.318280262 | Down |
| 2-Amino-4-(5-bromo-2-thienyl)-7-methyl-5-oxo-4H,5H-pyrano[4,3-b]pyran-3-carbonitrile | 1.717261689 | 0.010226357 | -3.318472173 | Down |
| 3-Methyl-6-oxo-1-phenyl-4-(3-pyridinyl)-6,7-dihydro-1H-pyrazolo[3,4-b]pyridine-5-carbonitrile | 1.934539885 | 0.006984407 | -3.324131443 | Down |
| _120254 | 1.82659757 | 0.012614123 | -3.388506912 | Down |
| Dioxathion | 1.63322754 | 0.015670823 | -3.402099754 | Down |
| (+/-)-threo-3,4-Dichloromethylphenidate | 1.647743481 | 0.008555662 | -3.420998435 | Down |
| 1-(16-Acetyl-1,4,10,13-tetraoxa-7,16-diaza-cyclooctadec-7-yl)-ethanone | 2.225816536 | 4.48328E-05 | -3.463740403 | Down |
| Tolcapone | 1.655181577 | 0.005345824 | -3.495218839 | Down |
| Adefovir | 1.554216112 | 0.011157646 | -3.498996361 | Down |
| Deoxyloganin tetraacetate | 1.411220996 | 0.010671393 | -3.550454097 | Down |
| N1-(1,3,5-trimethyl-1H-pyrazol-4-yl)-2-{[(2,5-dichloro-3-thienyl)sulfonyl]amino}benzamide | 1.643772931 | 0.014093722 | -3.567931018 | Down |
| NP-006274 | 2.084982029 | 0.001685044 | -3.579027986 | Down |
| 4-(Palmitoylamino)-1-naphthalenesulfonic acid | 1.297778843 | 0.036515056 | -3.587836561 | Down |
| glu-ile-ser | 2.256700439 | 7.9146E-05 | -3.680098711 | Down |
| {(2R,4S,5R)-5-[1-Methyl-3-(2-thienyl)-1H-pyrazol-5-yl]-1-azabicyclo[2.2.2]oct-2-yl}methyl isopropylcarbamate | 2.128305815 | 0.005685478 | -3.81525212 | Down |
| Aurantio-obtusin | 1.02578585 | 0.012238207 | -3.890983959 | Down |
| 6-O-Feruloylcatalpol | 1.318486011 | 0.029625537 | -3.896923464 | Down |
| Taurocholic acid | 1.121670371 | 0.046755094 | -3.920630117 | Down |
| 3-{4-[(4-Fluorophenyl)sulfonyl]piperazino}-4H-chromen-4-one | 1.252590786 | 0.012463641 | -3.943511196 | Down |
| 17-dimethylarsinoyl-9Z-heptadecenoic acid | 2.17935527 | 0.003873382 | -3.949463725 | Down |
| L-alpha-Glutamyl-L-valyl-L-leucyl-L-phenylalanyl-D-asparagine | 2.007126247 | 0.037553482 | -4.457418924 | Down |
| (8S,11R,12S)-N~12~-Hydroxy-11-isobutyl-N~8~-[2-(4-morpholinyl)-2-oxoethyl]-2,10-dioxo-1-oxa-3,9-diazacyclopentadecane-8,12-dicarboxamide | 2.118701503 | 0.028281848 | -4.475362658 | Down |
| Pyrrhoxanthin | 2.171827603 | 0.046808088 | -6.359856098 | Down |
| (6S,9S,12S,15S)-12-(Carboxymethyl)-15-isobutyl-6-isopropyl-2-methyl-4,7,10,13-tetraoxo-9-[2-oxo-2-(1-pyrrolidinyl)ethyl]-3,5,8,11,14-pentaazahexadecan-16-oic acid | 2.220317968 | 0.037758179 | -7.115068705 | Down |

**Supplementary Table 3.** Differential metabolites in the serum of chronic pancreatitis (CP) and negative control (NC) mice.

## Supplementary Table 4

| id | Symbol | CP | NC | Log2FC | P value |
| --- | --- | --- | --- | --- | --- |
| ENSMUSG00000000120 | Ngfr | 1.957 | 4.0507 | -1.049516 | 0.047863 |
| ENSMUSG00000000263 | Glra1 | 0.479 | 1.6643 | -1.796847 | 0.043992 |
| ENSMUSG00000000782 | Tcf7 | 3.6193 | 5.845 | -0.691479 | 0.00608 |
| ENSMUSG00000002633 | Shh | 1.8493 | 2.8623 | -0.630186 | 0.046003 |
| ENSMUSG00000004341 | Gpx6 | 0.2257 | 0.9277 | -2.039413 | 0.047364 |
| ENSMUSG00000004347 | Pde1c | 3.473 | 6.7423 | -0.957066 | 0.016093 |
| ENSMUSG00000004894 | Hapln2 | 13.764 | 20.6597 | -0.585917 | 0.021909 |
| ENSMUSG00000004952 | Rasa4 | 2.376 | 4.572 | -0.944291 | 0.02829 |
| ENSMUSG00000006014 | Prg4 | 17.7137 | 7.2723 | 1.284373 | 0.001053 |
| ENSMUSG00000006574 | Slc4a1 | 0.2613 | 0.038 | 2.78182 | 0.0361 |
| ENSMUSG00000009075 | Cabp7 | 45.309 | 24.2 | 0.904791 | 0.008563 |
| ENSMUSG00000010797 | Wnt2 | 2.5713 | 4.9347 | -0.940436 | 0.005979 |
| ENSMUSG00000014158 | Trpv4 | 3.9397 | 1.9723 | 0.99817 | 0.012511 |
| ENSMUSG00000014301 | Pam16 | 25.577 | 15.0483 | 0.765243 | 0.046948 |
| ENSMUSG00000016496 | Cd274 | 0.8073 | 1.5093 | -0.902675 | 0.044412 |
| ENSMUSG00000017146 | Brca1 | 0.4937 | 1.4467 | -1.551123 | 0.04573 |
| ENSMUSG00000017405 | Nek8 | 1.1093 | 1.836 | -0.726873 | 0.044113 |
| ENSMUSG00000017491 | Rarb | 10.6973 | 20.921 | -0.967701 | 0.000089 |
| ENSMUSG00000017830 | Dhx58 | 0.654 | 1.4283 | -1.12697 | 0.031277 |
| ENSMUSG00000019122 | Ccl9 | 0.778 | 1.398 | -0.845522 | 0.040118 |
| ENSMUSG00000019990 | Pde7b | 30.0627 | 48.4503 | -0.688534 | 0.000024 |
| ENSMUSG00000020599 | Rgs9 | 43.6373 | 95.2713 | -1.126479 | 0.000037 |
| ENSMUSG00000020641 | Rsad2 | 0.6943 | 1.3363 | -0.94458 | 0.040673 |
| ENSMUSG00000020681 | Ace | 32.59 | 18.971 | 0.780634 | 0.045213 |
| ENSMUSG00000020774 | Aspa | 14.3807 | 23.4123 | -0.703138 | 0.004105 |
| ENSMUSG00000020807 | 4933427D14Rik | 2.159 | 6.096 | -1.4975 | 0.000204 |
| ENSMUSG00000021108 | Prkch | 6.256 | 10.511 | -0.748588 | 0.001579 |
| ENSMUSG00000021223 | Papln | 0.7713 | 1.3497 | -0.807177 | 0.032117 |
| ENSMUSG00000021478 | Drd1 | 20.1603 | 35.7483 | -0.826356 | 0.001269 |
| ENSMUSG00000021919 | Chat | 3.4387 | 6.9257 | -1.010104 | 0.001842 |
| ENSMUSG00000022061 | Nkx3-1 | 0.837 | 1.6557 | -0.984113 | 0.032796 |
| ENSMUSG00000022176 | Rem2 | 12.085 | 18.6753 | -0.627917 | 0.004208 |
| ENSMUSG00000022996 | Wnt10b | 5.639 | 3.3147 | 0.766576 | 0.037818 |
| ENSMUSG00000023868 | Pde10a | 60.9573 | 104.8767 | -0.782822 | 0.00055 |
| ENSMUSG00000023908 | Pkmyt1 | 2.2027 | 3.93 | -0.835278 | 0.01078 |
| ENSMUSG00000023945 | Slc5a7 | 2.1797 | 5.3337 | -1.29102 | 0.000336 |
| ENSMUSG00000024397 | Aif1 | 5.061 | 7.6853 | -0.602685 | 0.043843 |
| ENSMUSG00000024401 | Tnf | 0.069 | 0.4663 | -2.756693 | 0.048267 |
| ENSMUSG00000024810 | Il33 | 28.612 | 45.4543 | -0.667797 | 6.35E-06 |
| ENSMUSG00000025128 | Bhlhe22 | 27.7387 | 17.734 | 0.64538 | 0.01637 |
| ENSMUSG00000025154 | Arhgap19 | 2.387 | 3.692 | -0.629204 | 0.022449 |
| ENSMUSG00000025270 | Alas2 | 8.1743 | 4.1697 | 0.971169 | 0.019475 |
| ENSMUSG00000025498 | Irf7 | 2.0577 | 5.9497 | -1.5318 | 0.000213 |
| ENSMUSG00000026051 | Ecrg4 | 21.189 | 8.8193 | 1.264574 | 0.047745 |
| ENSMUSG00000026162 | Nhej1 | 1.086 | 2.3343 | -1.103986 | 0.033924 |
| ENSMUSG00000026333 | Gin1 | 4.071 | 6.411 | -0.655166 | 0.021295 |
| ENSMUSG00000026354 | Lct | 1.5043 | 0.6243 | 1.268736 | 0.034008 |
| ENSMUSG00000026822 | Lcn2 | 5.857 | 2.2513 | 1.379382 | 0.040597 |
| ENSMUSG00000026826 | Nr4a2 | 33.51 | 19.8097 | 0.758387 | 0.007816 |
| ENSMUSG00000026830 | Ermn | 23.0633 | 35.2523 | -0.612118 | 0.002897 |
| ENSMUSG00000026834 | Acvr1c | 3.7927 | 6.5443 | -0.787034 | 0.002358 |
| ENSMUSG00000026896 | Ifih1 | 1.2663 | 2.513 | -0.988753 | 0.005739 |
| ENSMUSG00000027009 | Itga4 | 5.023 | 2.7763 | 0.855368 | 0.014184 |
| ENSMUSG00000027356 | Fermt1 | 0.398 | 0.0433 | 3.199219 | 0.011223 |
| ENSMUSG00000027568 | Ntsr1 | 7.3203 | 4.304 | 0.766231 | 0.016251 |
| ENSMUSG00000027861 | Casq2 | 1.29 | 2.7687 | -1.10182 | 0.002685 |
| ENSMUSG00000028072 | Ntrk1 | 1.355 | 3.1817 | -1.23149 | 0.014957 |
| ENSMUSG00000028268 | Gbp3 | 1.7723 | 4.2963 | -1.277456 | 0.003243 |
| ENSMUSG00000028871 | Rspo1 | 4.5 | 7.092 | -0.656268 | 0.016544 |
| ENSMUSG00000029136 | Rbks | 1.305 | 2.7513 | -1.076081 | 0.038816 |
| ENSMUSG00000029219 | Slc10a4 | 4.9227 | 8.7307 | -0.826652 | 0.004774 |
| ENSMUSG00000029561 | Oasl2 | 2.786 | 5.5953 | -1.006029 | 0.005062 |
| ENSMUSG00000030043 | Tacr1 | 3.2587 | 6.1847 | -0.924414 | 0.008413 |
| ENSMUSG00000030107 | Usp18 | 0.968 | 2.3447 | -1.276304 | 0.02118 |
| ENSMUSG00000030680 | Pagr1a | 0.8533 | 4.494 | -2.396819 | 0.000031 |
| ENSMUSG00000031139 | Mcf2 | 2.811 | 4.9297 | -0.810407 | 0.00567 |
| ENSMUSG00000031340 | Gabre | 0.4187 | 0.885 | -1.079875 | 0.041752 |
| ENSMUSG00000031351 | Zfp185 | 2.0867 | 0.9313 | 1.163831 | 0.018275 |
| ENSMUSG00000031425 | Plp1 | 506.8337 | 795.2453 | -0.649888 | 0.00125 |
| ENSMUSG00000031551 | Ido1 | 2.0143 | 4.9667 | -1.301975 | 0.000924 |
| ENSMUSG00000031928 | Mre11a | 5.9683 | 11.034 | -0.886556 | 0.009504 |
| ENSMUSG00000032343 | Impg1 | 0.4813 | 1.2847 | -1.416286 | 0.002836 |
| ENSMUSG00000032484 | Ngp | 1.878 | 0.1817 | 3.369831 | 0.007353 |
| ENSMUSG00000032690 | Oas2 | 0.131 | 0.503 | -1.940992 | 0.032879 |
| ENSMUSG00000032841 | Prr5l | 9.743 | 15.545 | -0.674013 | 0.006529 |
| ENSMUSG00000032854 | Ugt8a | 12.715 | 19.2713 | -0.599925 | 0.000119 |
| ENSMUSG00000033355 | Rtp4 | 3.6297 | 7.3423 | -1.016402 | 0.009601 |
| ENSMUSG00000033491 | Prss35 | 1.6263 | 2.4417 | -0.586243 | 0.035808 |
| ENSMUSG00000034459 | Ifit1 | 1.617 | 3.7207 | -1.202241 | 0.011477 |
| ENSMUSG00000034641 | Cd300ld | 0 | 0.2277 | -7.830779 | 0.015956 |
| ENSMUSG00000034701 | Neurod1 | 10.5527 | 6.1933 | 0.76882 | 0.00888 |
| ENSMUSG00000035208 | Slfn8 | 0.3603 | 1.0937 | -1.601769 | 0.045533 |
| ENSMUSG00000035299 | Mid1 | 19.453 | 8.8617 | 1.134343 | 0.000109 |
| ENSMUSG00000035606 | Ky | 1.014 | 1.5403 | -0.603185 | 0.036559 |
| ENSMUSG00000035683 | Melk | 0.502 | 1.19 | -1.245202 | 0.013223 |
| ENSMUSG00000036181 | H1f2 | 21.3563 | 33.694 | -0.657828 | 0.012319 |
| ENSMUSG00000036545 | Adamts2 | 7.5107 | 4.7653 | 0.656364 | 0.020704 |
| ENSMUSG00000036907 | C1ql2 | 8.43 | 3.349 | 1.331802 | 0.02016 |
| ENSMUSG00000037139 | Myom3 | 0.9763 | 1.5287 | -0.646828 | 0.018905 |
| ENSMUSG00000037321 | Tap1 | 1.8973 | 3.3997 | -0.84142 | 0.001516 |
| ENSMUSG00000037362 | Ccn3 | 111.242 | 67.7267 | 0.715906 | 0.049818 |
| ENSMUSG00000038550 | Ciart | 38.3867 | 23.8957 | 0.683856 | 0.022419 |
| ENSMUSG00000038567 | Cyp24a1 | 0.1627 | 0 | 7.345775 | 0.027444 |
| ENSMUSG00000038805 | Six3 | 6.756 | 12.7337 | -0.914407 | 0.003264 |
| ENSMUSG00000039103 | Nexn | 2.535 | 8.2553 | -1.703341 | 3.81E-06 |
| ENSMUSG00000040170 | Fmo2 | 3.656 | 2.261 | 0.693305 | 0.010339 |
| ENSMUSG00000040483 | Xaf1 | 4.5547 | 8.47 | -0.895016 | 0.001965 |
| ENSMUSG00000041538 | H2-Ob | 0.1237 | 0.5827 | -2.236214 | 0.024427 |
| ENSMUSG00000041669 | Prima1 | 3.4747 | 6.7873 | -0.96597 | 0.013937 |
| ENSMUSG00000042258 | Isl1 | 1.5567 | 2.913 | -0.904046 | 0.011836 |
| ENSMUSG00000044006 | Cilp2 | 0.6343 | 0.2227 | 1.510356 | 0.047969 |
| ENSMUSG00000044121 | 5430402E10Rik | 0 | 1.223 | -10.256209 | 0.026518 |
| ENSMUSG00000044177 | Wfikkn2 | 4.1483 | 1.68 | 1.304071 | 0.017706 |
| ENSMUSG00000045322 | Tlr9 | 1.0273 | 1.7693 | -0.784302 | 0.021577 |
| ENSMUSG00000045441 | Gprin3 | 2.7493 | 4.515 | -0.715644 | 0.010154 |
| ENSMUSG00000045573 | Penk | 323.4327 | 561.3427 | -0.795416 | 0.040143 |
| ENSMUSG00000045868 | Gvin1 | 0.4027 | 1.0803 | -1.423819 | 0.015175 |
| ENSMUSG00000045991 | Onecut2 | 0.2727 | 0.5583 | -1.033988 | 0.004739 |
| ENSMUSG00000046056 | Sbsn | 2.722 | 5.61 | -1.043334 | 0.048634 |
| ENSMUSG00000046561 | Arsj | 2.2267 | 1.0617 | 1.068555 | 0.013562 |
| ENSMUSG00000046922 | Gpr6 | 12.2783 | 22.075 | -0.846299 | 0.011346 |
| ENSMUSG00000047109 | Cldn14 | 0.8063 | 2.0083 | -1.31655 | 0.040846 |
| ENSMUSG00000047443 | Erfe | 0.463 | 0.877 | -0.921565 | 0.027397 |
| ENSMUSG00000047875 | Gpr157 | 0.977 | 1.6323 | -0.740505 | 0.015452 |
| ENSMUSG00000047907 | Tshz2 | 42.158 | 24.611 | 0.776503 | 0.000508 |
| ENSMUSG00000048038 | Ccdc187 | 1.0823 | 1.6843 | -0.638033 | 0.006349 |
| ENSMUSG00000048240 | Gng7 | 209.3057 | 318.4287 | -0.605359 | 0.004217 |
| ENSMUSG00000048292 | Or10v5 | 0.0523 | 0.2903 | -2.471908 | 0.026465 |
| ENSMUSG00000049252 | Lrp1b | 8.3617 | 13.7047 | -0.712805 | 0.016341 |
| ENSMUSG00000049511 | Htr1b | 7.643 | 12.2227 | -0.677348 | 0.04014 |
| ENSMUSG00000049928 | Glp2r | 3.3997 | 1.6413 | 1.050525 | 0.011745 |
| ENSMUSG00000050121 | Opalin | 15.2693 | 25.2063 | -0.723149 | 0.000379 |
| ENSMUSG00000050473 | Slc35d3 | 3.776 | 6.6803 | -0.823061 | 0.01502 |
| ENSMUSG00000050700 | Emilin3 | 0 | 0.1653 | -7.369234 | 0.020458 |
| ENSMUSG00000050854 | Tmem125 | 5.459 | 8.986 | -0.719042 | 0.014627 |
| ENSMUSG00000052776 | Oas1a | 0.406 | 1.1213 | -1.465664 | 0.039219 |
| ENSMUSG00000053007 | Creb5 | 1.3033 | 2.0563 | -0.657868 | 0.039454 |
| ENSMUSG00000053063 | Clec12a | 0.302 | 1.5597 | -2.368617 | 0.001275 |
| ENSMUSG00000054360 | Bsx | 0 | 0.1927 | -7.589963 | 0.027336 |
| ENSMUSG00000054667 | Irs4 | 0.952 | 1.9087 | -1.003532 | 0.011479 |
| ENSMUSG00000055639 | Dach1 | 3.7733 | 6.561 | -0.798076 | 0.004544 |
| ENSMUSG00000056174 | Col8a2 | 4.6147 | 2.2883 | 1.011929 | 0.036634 |
| ENSMUSG00000057137 | Tmem140 | 1.18 | 2.322 | -0.976581 | 0.044207 |
| ENSMUSG00000057337 | Chst3 | 0.8383 | 1.435 | -0.775455 | 0.015844 |
| ENSMUSG00000057440 | Mpp7 | 2.4467 | 4.5947 | -0.909143 | 0.028956 |
| ENSMUSG00000057719 | Sh3rf2 | 3.618 | 7.074 | -0.967334 | 0.00597 |
| ENSMUSG00000058260 | Serpina9 | 3.1173 | 7.7687 | -1.317355 | 0.000423 |
| ENSMUSG00000059089 | Fcgr4 | 0.197 | 0.8577 | -2.122221 | 0.042897 |
| ENSMUSG00000060962 | Dmkn | 3.4593 | 7.5297 | -1.122092 | 0.011071 |
| ENSMUSG00000061762 | Tac1 | 70.1103 | 115.1167 | -0.715398 | 0.001155 |
| ENSMUSG00000062151 | Unc13c | 11.48 | 22.8747 | -0.994628 | 0.028768 |
| ENSMUSG00000062393 | Dgkk | 0.578 | 1.1063 | -0.936645 | 0.013437 |
| ENSMUSG00000062488 | Ifit3b | 3.2717 | 6.748 | -1.044434 | 0.000372 |
| ENSMUSG00000063895 | Nup58 | 0.18 | 0 | 7.491853 | 0.043142 |
| ENSMUSG00000064179 | Tnnt1 | 10.8097 | 25.8953 | -1.26037 | 0.004504 |
| ENSMUSG00000064215 | Ifi27 | 36.541 | 61.6913 | -0.755552 | 0.009252 |
| ENSMUSG00000067684 | Obp1a | 0 | 1.0513 | -10.038004 | 0.043026 |
| ENSMUSG00000067724 | Gbx1 | 0.348 | 0.9173 | -1.398359 | 0.035266 |
| ENSMUSG00000068428 | Gmnc | 0.7673 | 0.187 | 2.036815 | 0.04785 |
| ENSMUSG00000069917 | Hba-a2 | 353.8453 | 217.6957 | 0.700806 | 0.039985 |
| ENSMUSG00000069919 | Hba-a1 | 270.9397 | 169.176 | 0.679447 | 0.02652 |
| ENSMUSG00000071234 | Syndig1l | 34.2963 | 55.5497 | -0.695724 | 0.002277 |
| ENSMUSG00000071547 | Nt5dc2 | 13.592 | 8.1327 | 0.740957 | 0.020148 |
| ENSMUSG00000073489 | Ifi204 | 0.2347 | 0.9543 | -2.02388 | 0.011085 |
| ENSMUSG00000074771 | Ankef1 | 0.2617 | 0.689 | -1.396774 | 0.040053 |
| ENSMUSG00000074896 | Ifit3 | 10.5167 | 19.556 | -0.894934 | 0.003028 |
| ENSMUSG00000078921 | Tgtp2 | 1.0573 | 1.987 | -0.910162 | 0.048526 |
| ENSMUSG00000083649 | Rasl2-9 | 0 | 0.5613 | -9.132714 | 0.022481 |
| ENSMUSG00000096225 | Lhx8 | 1.0483 | 4.9627 | -2.243018 | 0.000033 |
| ENSMUSG00000096549 | Prickle4 | 1.489 | 0.199 | 2.903503 | 0.041213 |
| ENSMUSG00000098112 | Bin2 | 2.1913 | 3.8813 | -0.824743 | 0.034503 |
| ENSMUSG00000103421 | Golt1a | 0.7687 | 0.038 | 4.338287 | 0.023581 |
| ENSMUSG00000103442 | Pcdha1 | 1.4383 | 2.2017 | -0.614198 | 0.021555 |
| ENSMUSG00000109061 | Gm49320 | 0.365 | 1.0617 | -1.540363 | 0.00905 |
| ENSMUSG00000116024 | Gm49527 | 0.546 | 1.823 | -1.739342 | 0.015061 |
| ENSMUSG00000116876 | Gm49721 | 0 | 2.1127 | -11.044849 | 2.85E-09 |
| ENSMUSG00000118401 | Gpr52 | 10.6797 | 16.2967 | -0.60971 | 0.027487 |
| ENSMUSG00000121570 | - | 0 | 5.358 | -12.387479 | 8.18E-07 |

**Supplementary Table 4.** Transcriptomic analysis of differentially expressed genes in the cerebral cortex of mice between negative control (NC) and chronic pancreatitis (CP) groups.
